# Probing the target search of DNA-binding proteins in mammalian cells using TetR as model searcher

**DOI:** 10.1038/ncomms8357

**Published:** 2015-07-07

**Authors:** Davide Normanno, Lydia Boudarène, Claire Dugast-Darzacq, Jiji Chen, Christian Richter, Florence Proux, Olivier Bénichou, Raphaël Voituriez, Xavier Darzacq, Maxime Dahan

**Affiliations:** 1 Laboratoire Kastler Brossel, CNRS UMR 8552, École normale supérieure, Université Pierre et Marie Curie, Paris 6, 46 rue d'Ulm, 75005 Paris, France; 2 Functional Imaging of Transcription, CNRS UMR 8197, École normale supérieure, Institut de Biologie de l'ENS, IBENS, 46 rue d'Ulm, 75005 Paris, France; 3 Transcription Imaging Consortium, Janelia Research Campus, Howard Hughes Medical Institute, 19700 Helix Drive, Ashburn, Virginia 20147, USA; 4 Physico-Chimie Curie, Institut Curie, CNRS UMR 168, Université Pierre et Marie Curie, Paris 6, 26 rue d'Ulm, 75005 Paris, France; 5 Université Paris-Diderot, Paris 7, 5 rue Thomas Mann, 75013 Paris, France; 6 Laboratoire de Physique Théorique de la Matière Condensée, CNRS UMR 7600, Université Pierre et Marie Curie, Paris 6, 4 place Jussieu, 75005 Paris, France

## Abstract

Many cellular functions rely on DNA-binding proteins finding and associating to specific sites in the genome. Yet the mechanisms underlying the target search remain poorly understood, especially in the case of the highly organized mammalian cell nucleus. Using as a model Tet repressors (TetRs) searching for a multi-array locus, we quantitatively analyse the search process in human cells with single-molecule tracking and single-cell protein–DNA association measurements. We find that TetRs explore the nucleus and reach their target by 3D diffusion interspersed with transient interactions with non-cognate sites, consistent with the facilitated diffusion model. Remarkably, nonspecific binding times are broadly distributed, underlining a lack of clear delimitation between specific and nonspecific interactions. However, the search kinetics is not determined by diffusive transport but by the low association rate to nonspecific sites. Altogether, our results provide a comprehensive view of the recruitment dynamics of proteins at specific loci in mammalian cells.

## Abstract (web summary)

During transcription, replication and repair, DNA-binding proteins must find specific interaction sites hidden within a vast excess of genomic DNA. Here the authors use single-molecule tracking to quantitatively determine the contributions of the different processes that underlie target search in human cells.

## Graphical abstract

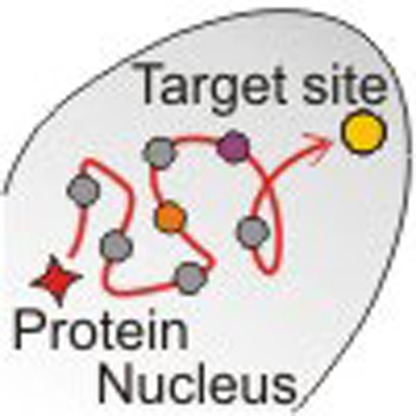

Key cellular functions, such as transcription, replication and repair, are governed by the association of DNA-binding proteins (DBPs) to specific DNA sequences in the genome. Understanding the mechanisms by which DBPs find their target sites (∼10 bp long) within genomes made of millions to billions of base pairs, and quantifying the associated search kinetics, is pivotal to analyse biochemical reactions and their regulation in living cells^1^,^2^. The search process can be schematically divided into two steps: transport through the nucleus followed by biochemical association to the target. Historically, much attention has been focused on the kinetics of the transport mechanisms of DBPs, trying to explain the ‘faster-than-diffusion' association rate reported for the LacI^3^. The predominant target search (TS) model^4^, called facilitated diffusion (FD), postulates that the search results from one-dimensional (1D) sliding events (with duration *τ*_1D_), during which proteins diffuse along nonspecific DNA sequences, interspersed with phases (with duration *τ*_3D_) of three-dimensional (3D) diffusion^5^,^6^. The TS mechanisms have been the subject of controversies^7^ but single-molecule (SM) experiments have recently provided supporting evidence for the FD model *in vitro*^8^,^9^,^10^,^11^ and in *E. coli* cells^12^,^13^. In eukaryotic cells, where DNA is packaged into chromatin fibres and the nucleus is highly compartmentalized and organized^14^,^15^, DBPs have to identify their targets among a much larger number of non-cognate sites than in prokaryotes^16^. While recent experiments have pointed to the role of nonspecific interactions in the dynamics of nuclear factors in mammalian nuclei^17^,^18^, the role of FD remains ill understood^19^. Moreover, the binding efficiency once the DBPs reach the specific site is often not considered although it is essential to determine which step, diffusive transport or binding, is limiting the association kinetics in the nucleus of mammalian cells.

Here we quantitatively investigate the TS of DBPs in human cells. We use Tet repressors (TetRs) as searchers in the nucleus of human cells in which a gene array serves as target locus. With this model system, we can unequivocally distinguish the role of specific and nonspecific interactions. Thereby, we identify the role of FD in the TS and measure transport and binding parameters underlying the search kinetics. We find that nonspecific binding times are broadly distributed, with no clear delimitation between specific and nonspecific interactions. In addition, we measure at the single-cell level the association kinetics to the target locus. Importantly, the association rate is not limited by transport but, instead, by the binding to nonspecific DNA sequences. Altogether, our results provide a quantitative description of the TS dynamics and shed a new light on the factors controlling the search kinetics.

## Results

### A single-molecule TS assay in human cells

Since the initial observations of the rapid mobility of nuclear factors^20^, the dynamics of proteins in the nucleus has been addressed using a variety of experimental techniques, either at the population or, more recently, at the single-molecule level (see ref. 21 for a review). Here we chose to probe the search dynamics of DBPs in human cells by means of a single-molecule assay^17^,^18^,^22^, because SM experiments are able to quantify the stochasticity and heterogeneity of molecular interactions with high spatial and temporal resolution.

**Figure 1:**
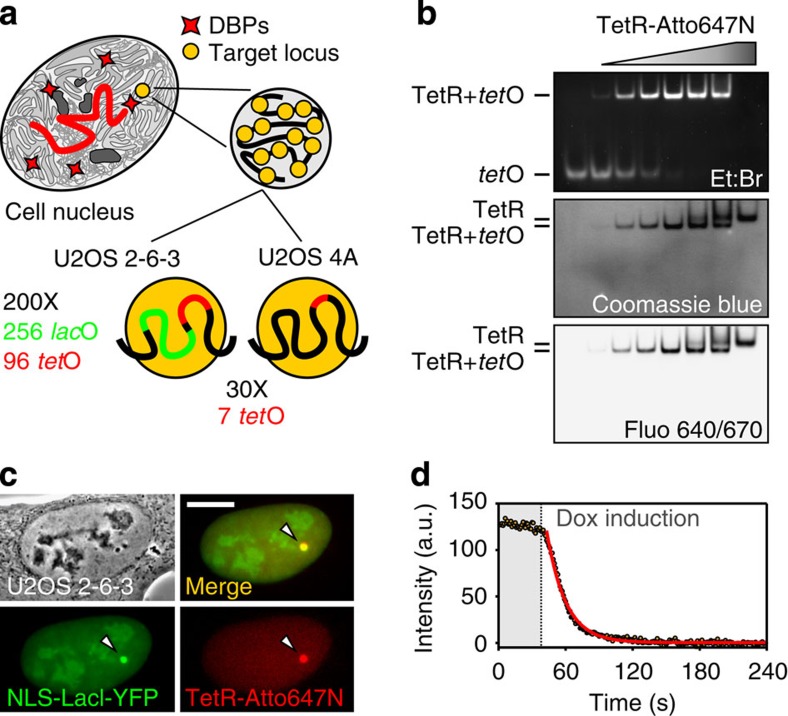
TS assay. (**a**) Schematic drawing of the cellular system based on U2OS cells containing at a single locus in the genome a target site consisting in either 200 inserts, each containing also 256 *lac*O and 96 *tet*O binding sites (U2OS 2-6-3), or 30 insertions of 7 *tet*O sites (U2OS 4A). (**b**) Native-conditions gel-shift assay showing TetR-Atto647N capability to bind to the *tet*O *in vitro*. (**c**) Phase image of the nucleus of a U2OS 2-6-3 cell (grey scale), fluorescence images of NLS-LacI-YFP (green) and of TetR-Atto647N (red); the merge of the two channels (yellow) shows that both proteins are correctly recruited at the target site (white arrows). Scale bar, 5 μm. (**d**) Dox-induced release kinetics of TetR-Atto647N from the target site. Brown circles represent the fluorescence intensity of the target site during Dox treatment (Dox final concentration 2.5 μg ml^−1^). Red curve is a monoexponential fit of the data yielding a dissociation decay time *τ*_Dox_ of the order of 16 s for the cell in the example.

Most endogenous DBPs have multiple specific binding sites, scattered all over the genome. Besides particular cases, such as transcriptionally productive domains (ref. 23), imaging the specific sites and locating them exhaustively remain challenging. Therefore, we used an engineered cellular system in which sites of specific and nonspecific interactions could be unambiguously distinguished. As model searcher, we chose the bacterial TetR protein^24^, a DBP that recognizes a 19bp long specific DNA sequence (*tet*O) with high affinity (the TetR-*tet*O binding constant measured at physiologically relevant ionic strength—160 nM NaCl—is 2 × 10^11^ M^−1^, ref. 25), and that is widely used for transcriptional control in eukaryotic systems^26^. Importantly, TetR has no specific binding sites in the human genome and its affinity for DNA can be tightly controlled with an allosteric inducer, the doxycycline (Dox), which decreases TetR affinity for *tet*O by nine orders of magnitude^27^. We probed TetR TS dynamics in the nucleus of human osteosarcoma cells (U2OS) carrying, at a single locus in the genome (target), repeated insertions of *tet*O binding sites (Supplementary Note 1). The use of repeated insertions was necessary for detecting TetRs bound at specific sites and discriminating them from molecules unbound or associated to non-cognate sites. In practice, we used U2OS 2-6-3 cells^28^ with 200 inserts of a gene cassette, each containing also 256 *lac*O and 96 *tet*O repeats, and U2OS 4A cells with 30 insertions of 7 *tet*O sites (Fig. 1a and Supplementary Fig. 1). TetR proteins were purified and site-specifically labelled with Atto647N, a bright organic dye (Supplementary Note 2). The labelled proteins maintained their functionality *in vitro* (Fig. 1b) and, once microinjected in the nucleoplasm of U2OS 2-6-3 cells (Supplementary Movie 1), they were able to specifically bind to the target locus (identifiable with fluorescent LacI proteins^29^, Fig. 1c). Moreover, on addition of Dox (Fig. 1d and Supplementary Movie 2), the fluorescent TetRs dissociated from the locus in ∼10 s. Similar results for the binding and the release kinetics were obtained in the case of cells expressing TetR fused to green fluorescent protein (GFP; Supplementary Note 3).

### TetR mobility analysis by single-particle tracking

**Figure 2:**
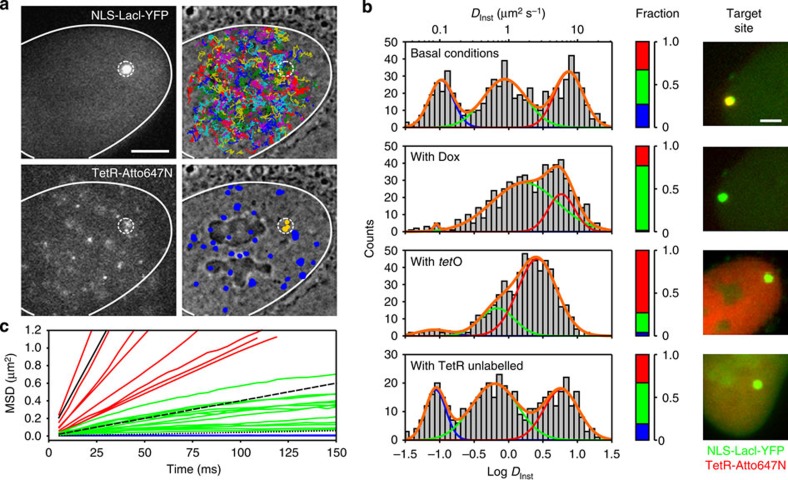
Single-molecule analysis of TetR-Atto647N nuclear exploration. (**a**) Top Left: fluorescence image of NLS-LacI-YFP showing the binding locus position (white dashed circle) in the cell nucleus (the white continuous line indicates the nuclear envelope contour). Bottom left: individual fluorescent spots of microinjected TetR-Atto647N molecules. Top right: representation of individual TetR-Atto647N trajectories superimposed to the bright field image of the cell nucleus. Bottom right: positions of the quasi-immobile molecules at nonspecific sites (blue spots) and at the target locus (yellow spots). Scale bar, 5 μm. (**b**) From top to bottom: histograms of the instantaneous diffusion coefficient *D*_Inst_ for TetR-Atto647N in basal conditions (without Dox, *N*=10 cells, *n*=682 trajectories); in the presence of 2.5 μg ml^−1^ Dox (*N*=8 cells, *n*=623 trajectories); when TetR-Atto647N was co-injected with 10 × molar excess of *tet*O oligos (*N*=4 cells, *n*=572 trajectories); and on co-injection with 1,000 × molar excess of unlabelled TetRs (*N*=3 cells, *n*=460 trajectories). The colour bars indicate the fraction of proteins in the fast (red), intermediate (green) and slow (blue) population, numerical quantification is reported in Supplementary Note 4. The images on the right show the occupancy of the target locus by NLS-LacI-YFP (green) and TetR-Atto647N (red). Scale bar, 2 μm. (**c**) Individual MSD versus time curves for TetR-Atto647N. Red curves correspond to rapidly free diffusing proteins, green curves to proteins belonging to the intermediate population and blue curves to quasi-immobile molecules. Black lines are guide-to-eye representing the MSD for a diffusion coefficient of 10 (continuous line), 1 (dashed line) and 0.1 μm^2^ s^−1^ (dotted line).

To investigate TetR-Atto647N dynamics, we injected them at low concentration (10–500 molecules per nucleus) and tracked single proteins at 197 frames per second with ∼25 nm localization accuracy (Fig. 2a, Supplementary Note 4 and Supplementary Movie 3). In individual trajectories away from the specific locus (Supplementary Movie 4), we determined the instantaneous diffusion coefficients (*D*_Inst_) from a linear fit of the initial points of the mean square displacement (MSD). The broad distribution of *D*_Inst_ could be sorted into three different mobility categories (Fig. 2b): a fast population (average diffusion coefficient *D*_1_∼8 μm^2^ s^−1^, fraction of the molecules *f*_1_∼33%) of freely diffusing proteins characterized by linear MSD curves (Fig. 2c, red lines), an intermediate one (*D*_2_∼1 μm^2^ s^−1^, *f*_2_∼43%) with sublinear (and in ∼20% of the cases confined, Supplementary Fig. 2) MSDs (Fig. 2c, green lines) and a third population (*D*_3_∼0.1 μm^2^ s^−1^, *f*_3_∼24%) characterized by a greatly reduced mobility and flat MSD curves (Fig. 2c, blue lines). Similar results were also found when tracking TetR labelled with other organic dyes with different chemical structure and electrical charge (Supplementary Note 4). To further validate our findings on the mobility of TetR, we performed single-particle tracking PALM (sptPALM) experiments^30^,^31^ on endogenously expressed TetRs fused to the photoconvertible protein Dendra2 (ref. 32, Supplementary Movie 5). Importantly, sptPALM data returned comparable values for the TetR diffusion coefficients and similar partitioning between the three different populations (Supplementary Note 5) than in the case of TetR ectopically microinjected. Furthermore, despite the less detailed level of information achievable, fluorescence recovery after photobleaching (FRAP) experiments on TetR-GFP (Supplementary Movie 6) confirmed the observed partitioning between nonspecifically bound (∼20%) and diffusing (∼80%) proteins (Supplementary Note 6). Thus, we concluded that tracking measurements reported on the intrinsic mobility and partitioning between the three different populations of TetR proteins.

The diffusive properties of quasi-immobile molecules (characterized by the diffusion coefficient *D*_3_) closely matched those of chromatin itself, as observed by tracking chromatin-bound markers such as histones H2B^22^,^31^. Also, the sites of immobilization did not show a preferential localization in the nucleus (blue points in bottom right image of Fig. 2a), ruling out that they correspond to a particular locus. The quasi-immobile molecules, as well as proteins bound at the target locus (yellow points in bottom right image of Fig. 2a), could be fully eliminated on incubation of the cells with Dox (at 2.5 μg ml^−1^) or when saturating the TetR DNA binding domain (DBD)–before injection— with short double-stranded DNA fragments containing the specific *tet*O sequence (Fig. 2b, second and third panel). Structural and electrostatic considerations support the view that specific and nonspecific DNA interactions occur via the DNA-binding helix-turn-helix motif of the TetR protein, which is the only positively charged domain in this protein. Thus, both types of interactions are expected to be modulated by Dox or by loading the DBD with *tet*O-containing oligos. Therefore, in the following, we considered that the population of quasi-immobile molecules corresponded to proteins nonspecifically bound to chromatin. In contrast, when TetR-Atto647Ns were co-injected with a 1,000-fold excess of unlabelled TetR, only the association to the specific locus was reduced (Fig. 2b, bottom panel), while the diffusion properties of the fluorescent proteins were unchanged and the quasi-immobile population preserved. This indicates that the nonspecific binding sites, apart from being scattered all over the nucleus, could not be saturated. This observation is consistent with the expected high abundance of nonspecific sites (the concentration *c*_DNA_ of base pairs of genomic DNA, accessible or not, in a 500 μm^3^ nucleus of human cells is ∼10^−2^ M). It should be noted that on Dox treatment or co-injection with *tet*O oligos, the diffusion coefficients of the two mobile populations (red and green, in Fig. 2b,c) were mostly unchanged. In the case of Dox treatment, we observed an increase of the relative abundance of the intermediate population (green coloured, characterized by *D*_2_). This might be due to some residual very rapid TetR–DNA interactions via the protein DBD, which are instead completely abolished by steric hindrance in the case of *tet*O co-injection. Nevertheless, the fact that in this latter case the intermediate population is still present indicates that the subdiffusive as well as the confined motion observed for the intermediate population is not only mediated by DNA interactions via the DBD of TetR but instead might depend on other factors such as protein–protein interactions (including crowding effects^33^) or the local nuclear architecture^34^,^35^. Indeed, recent reports have pointed out that the nuclear milieu has a role *per se* in controlling the diffusivity even of inert tracers^36^,^37^. DBPs have been shown to diffuse slower in heterochromatin regions^38^ and fast diffusing proteins tend to be excluded from H2B histone-enriched nuclear regions^39^.

Overall, we concluded that the molecules partitioned between those diffusing in the nucleoplasm (fast and intermediate populations) and those interacting nonspecifically with DNA (population of quasi-immobile proteins). The total fraction (*f*_1_+*f*_2_) ∼75% of diffusing proteins (consistent with sptPALM and FRAP measurements, Supplementary Notes 5 and 6) is equal to *τ*_3D_/(*τ*_1D_+*τ*_3D_). This leads to *τ*_3D_∼3*τ*_1D_ and places a constraint on the relative values of the dissociation rate (1/*τ*_1D_) and of the association rate (1/*τ*_3D_). Note that with current single-molecule tracking techniques, no direct evidence can be obtained by imaging on local sliding movement (or absence thereof) of the protein during nonspecific binding events.

### Kinetics of TetR interactions with non-cognate DNA

**Figure 3:**
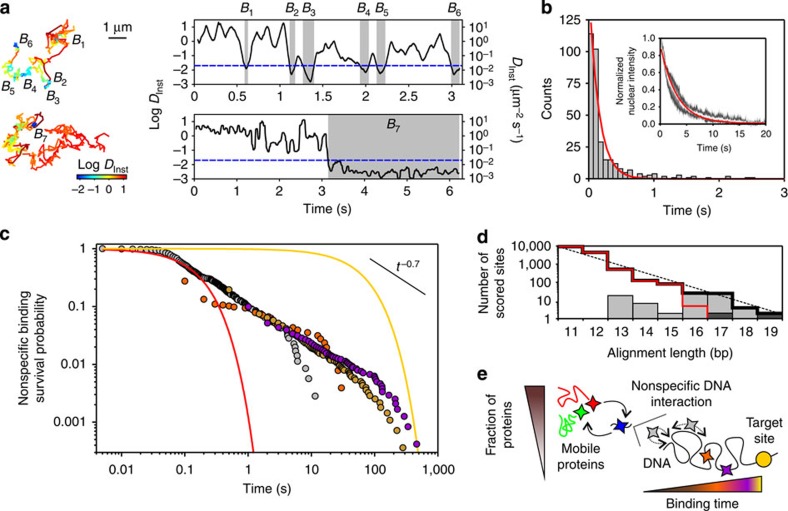
Nonspecific DNA-binding kinetics. (**a**) Two examples of trajectories showing transitions from diffusive state to a DNA-bound state and the corresponding time course of *D*_Inst_ computed over a running window of 80 ms and colour-coded according to the *D*_Inst_ value. DNA-binding events, labelled as *B*_i_, are identified via threshold analysis of the curve *D*_Inst_ versus time. Scale bar, 1 μm. (**b**) Distribution of the duration of the binding events obtained with the running window analysis. The red line corresponds to a monoexponential fit with a decay rate of 6.7 s^−1^. The inset shows the ensemble bleaching behaviour of TetR-Atto647N (white line)±one s.d. (grey area) for *N*=5 cells. The red line is a monoexponential fit with a decay rate of 0.34 s^−1^. (**c**) SP of the nonspecific binding events for continuous imaging (grey circles) and time-lapse experiments (*τ*_TL_=0.1 s orange circles, 0.5 s brown circles and 1 s purple circles). The red line represents the exponential decay fit shown in **b** and the yellow line the exponential decay corresponding to the dissociation time (*τ*_SPE_∼60 s) from the specific *tet*O sequence; the black line is a guide-to-eye corresponding to a power law *t*^γ^ with *γ*=−0.7. (**d**) The thick black line represents the total number of sites scored in the human genome using the BLAST algorithm, with the 19bplong canonical *tet*O sequence as query, as a function of the alignment length. The red line indicates the number of alignments with contiguous pairing to the *tet*O, dark grey rectangles represent sites with two mismatches and light grey ones sites with one mismatch. The dashed black line is an exponential fit to the total number of scored sites. (**e**) Schematic drawing of the nuclear dynamics of TetR-Atto647N. The vertical axis represents the abundance of the observed behaviour and the horizontal one the interaction time with non-cognate DNA sites (that is, the affinity for DNA): most of the protein (∼75%) are in a mobile state from which they can transition to a nonspecific DNA interaction state. The broad distribution of binding times on non-cognate DNA possibly reflects the hierarchy of binding affinity associated to the variability of nonspecific sequences, from completely random sequences to quasi-specific sites.

To closely examine the kinetics of nonspecific interactions and estimate *τ*_1D_, we analysed in individual trajectories (longer than 0.5 s) the time course of the instantaneous diffusion coefficient computed over an 80ms running window (Supplementary Note 7). In ∼50% of these trajectories, we could identify events in which the protein switched from fast or intermediate diffusion dynamics to a much slower motion (Fig. 3a, upper panel, and Supplementary Movie 7). These events, which were not observed in the presence of Dox or for co-injection with *tet*O oligos (Supplementary Fig. 3), likely correspond to molecules transitioning between 3D diffusion and nonspecific interactions with DNA. The distribution *P* of their duration was approximately monoexponential with a decay rate of 6.7 s^−1^ (Fig. 3b). After correction for the photobleaching rate (∼0.34 s^−1^ in our imaging conditions, Fig. 3b inset), the corresponding binding time is ∼158 ms. This value is intermediate between the short nonspecific binding times of LacI in *E. coli* (<5 ms, ref. 12) and the longer ones reported for p53 (∼1.7 s, ref. 17), Sox2 (∼0.8 s, ref. 18) and dimeric GR receptors (∼1.5 s, but with no distinction between specific and nonspecific binding, ref. 22) in mammalian cells.

Besides the rapid nonspecific dissociation processes, we also noted the occurrence of less frequent but longer nonspecific binding events with duration often exceeding several seconds (Fig. 3a, bottom panel). Yet with continuous imaging, estimating their duration was delicate due to the difficulty of discriminating between dissociation and photobleaching. We thus recorded time-lapse movies in which 5 ms exposure time images were interspersed with dark periods of duration *τ*_TL_ equal to 0.1, 0.5 or 1 s. In time-lapse movies, TetR proteins were considered bound when they did not move by more than 1 pixel (160 nm) over at least two consecutive images. For each data set, we computed the survival probability (SP): 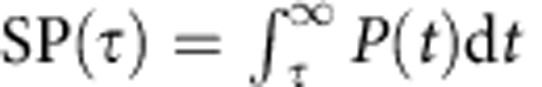, that is, the probability to stay bound for a time longer than *τ* (namely the complementary cumulative probability). The full SP, that includes the long binding events along the short ones, was obtained by renormalizing the SPs measured in time-lapse movies with the value of the SP obtained for continuous imaging at 1 s (Supplementary Note 7). Beyond ∼0.1 s, the SP curve markedly deviated from the exponential behaviour (Fig. 3c). Up to ∼100 s, a time comparable to the specific binding time (*τ*_SPE_∼60 s) of TetR to *tet*O as measured *in vitro* (Supplementary Fig. 4), it decreased as a power law *t*^*γ*^ with *γ*∼−0.7.

We first wondered whether the observed power law behaviour in the dissociation times could arise from microscopic hopping events where proteins unbind, with a single off rate, from nonspecific sites but quickly reattach to the same or to a closely located site, and which are misinterpreted as long events due to the limited spatial (25 nm) and temporal (10 ms) resolution of our imaging system. Yet, while hopping events possibly occur, they cannot account for the broad distribution of nonspecific binding times (Supplementary Note 7). On the contrary, our results strongly suggest a broad heterogeneity in nonspecific TetR–DNA interactions. Indeed, a well identified source of heterogeneity in the cell nucleus is the variability of DNA sequences—and of the corresponding binding energies^6^—and a power law distribution of binding times is expected in the case of exponentially distributed binding energies^40^,^41^.

Whereas most nonspecific binding events are short, denoting low affinity interactions with non-cognate sites, we used the Basic Local Alignment Search Tool (BLAST) algorithm^42^ to align the *tet*O sequence to the human genome and assess the possible existence and abundance of quasi-consensus sites for TetR. While the full length recognition sequence is not present in the human genome, we found thousands of sites that only partially differ from the *tet*O sequence (Supplementary Data 1). For instance, we found two sites (in chromosomes 4 and X) differing by two mismatches from the canonical 19bplong *tet*O sequence and, respectively, 4 and 23 sites with 18 and 17 bp similarity to *tet*O and a single mismatch. Overall, the number of scored sites increased exponentially for shorter alignment lengths (Fig. 3d). Even though the degree of similarity between a sequence and *tet*O does not directly relate to the TetR binding affinity, these quasi-consensus sequences are definitively potential candidates to act as stable off-target (decoy) sites. Furthermore, not only quasi-consensus sequences could behave as decoy sites but also certain repeat symmetries (outside of the specific binding sites) can control DNA-binding preferences, as recently shown by high throughput protein-DNA binding analysis^43^. We thus propose that the power law kinetics results from a broad heterogeneity of nonspecific binding sites (and binding energies) encountered by TetR in the human genome, ranging from very short residence time on completely random sequences (*τ*_RS_∼158 ms) to stable binding on sites that only partly differ from the 19bplong *tet*O specific sequence (Fig. 3e).

To evaluate the mean nonspecific binding time *τ*_1D_, we numerically integrated the survival probability SP distribution and found *τ*_1D_ to be ∼2 s, consistent with FRAP observations (Supplementary Note 6). Given that *τ*_3D_∼3*τ*_1D_, the mean 3D diffusion time is ∼6 s, meaning that the nonspecific sites are visited at a rate 1/(*τ*_1D_+*τ*_3D_)∼0.12 s^−1^. Importantly, *τ*_3D_ is much longer than 1/*Dac*_DNA_ ∼10^−5^ s, the time estimated for a diffusion-limited nonspecific association in the nucleus (with *a*=1 nm and the diffusion coefficient *D* on the order of *D*_1_). The high value of *τ*_3D_—or equivalently, the low association rate 1/*τ*_3D_—is possibly due to the inaccessibility of a large fraction of genomic DNA, to the inefficient binding of TetR to non-cognate sequences or to a combination thereof.

### Association kinetics at the target locus

**Figure 4:**
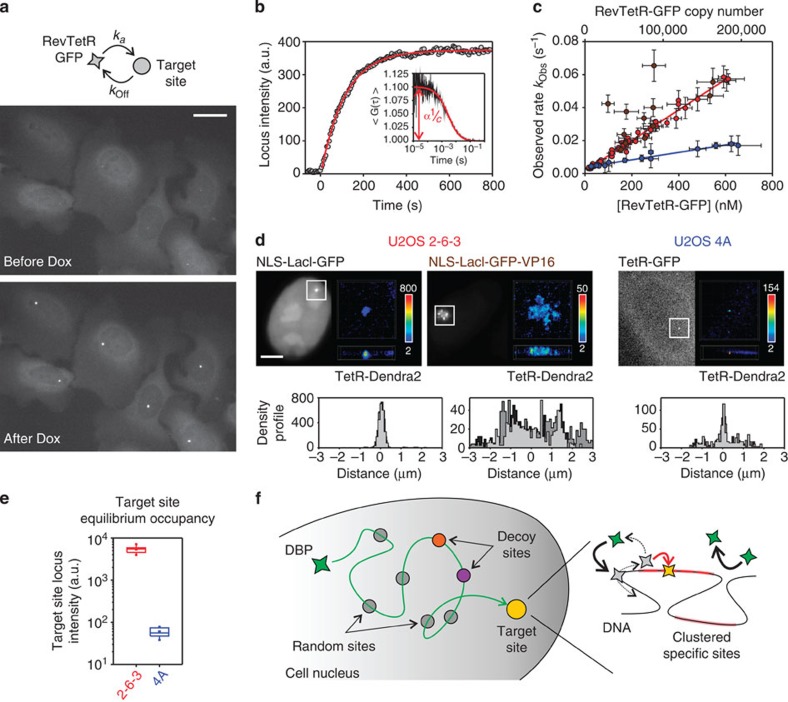
Association kinetics at the target site. (**a**) Fluorescence images of U2OS 2-6-3 cells expressing RevTetR-GFP before and after Dox addition (2.5 μg ml^−1^ final concentration). Scale bar, 5 μm. (**b**) Time course of the RevTetR-GFP fluorescence intensity at the binding locus. Inset: RevTetR-GFP two-photon FCS measurement. The amplitude of the autocorrelation function at short times yields the concentration *c* of RevTetR-GFP in the nucleus. (**c**) Observed rate *k*_Obs_ versus *c* for U2OS 2-6-3 cells (red), in the case of transcription activation using NLS-LacI-mCherry-VP16 (brown), and for U2OS 4A cells (blue). The slope of the linear fit (solid lines) provides an estimate of the association rate constant *k*_a_. The intercept at *c*=0 yields *k*_Off_∼0.001 s^−1^. Error bars represent s.d. (**d**) Conventional and super-resolution images of the binding locus using TetR-Dendra2 in combination with NLS-LacI-GFP and NLS-LacI-GFP-VP16 in U2OS 2-6-3 cells and TetR-GFP in U2OS 4A cells. Scale bar, 5 μm. Super-resolution images show a frontal view and a transversal section of a 5 μm × 5 μm × 1.2 μm nuclear region around the binding locus, colour code and colour bars indicate neighbours' density within a 75nm radius sphere. Bottom: density profiles of the binding locus for the three different conditions tested, obtained from the super-resolution images. In each case, we plot three representative profiles. (**e**) Fluorescence intensity of RevTetR-GFP at the binding locus long after Dox induction for the U2OS 2-6-3 (*I*_Locus_=5450±350 a.u., mean±s.d., *N*=9 cells) and U2OS 4A (*I*_Locus_=60±5 a.u., mean±s.d., *N*=9 cells) cell lines. (**f**) Schematic drawing of the search process: DBPs explore the nucleus by alternating between 3D diffusion and association to off-target sites (which in some case behave as decoy sites) until they associate in the vicinity of the target and finally slide along the DNA (dotted arrow) and bind to the specific binding site. During the TS process, the rate limiting step is the nonspecific association to DNA (black arrows), while once engaged on the DNA they can and effectively associate to the specific site (red arrow).

Having obtained a detailed view of the mechanisms governing protein mobility away from the specific sites, we next aimed to directly measure the search rate or, equivalently, the association rate constant of DBPs to the target locus in living cells. To this end, we used the Reverse TetR (RevTetR), a TetR point mutant for which DNA binding is inversely regulated by Dox^44^,^45^ and which has diffusive properties similar to that of TetR (in reversed Dox conditions, Supplementary Note 5). Importantly, RevTetR permits a more accurate temporal control on the association process than TetR (Supplementary Note 8). After Dox addition (at 2.5 μg ml^−1^) to U2OS 2-6-3 cells stably expressing RevTetR-GFP, we observed an accumulation of RevTetR-GFPs at the target locus (Fig. 4a and Supplementary Movie 8). The onset and increase of the fluorescence signal was independent of Dox concentration (over the range 2.5 to 125 μg ml^−1^, Supplementary Fig. 5), ruling out any possible influence of membrane permeability to Dox or RevTetR-GFP affinity for Dox on the association kinetics. The fluorescence time course at the locus was well fitted by a single exponential with an observed rate *k*_Obs_ (Fig. 4b). In each measured nucleus, we correlated *k*_Obs_ with the concentration *c* of RevTetR-GFP, measured with two-photon fluorescence correlation spectroscopy (FCS)^46^ (Fig. 4b inset and Supplementary Note 8) before RevTetR-GFP induction by Dox (when ∼90% of proteins is mobile in the nucleoplasm, Supplementary Notes 5 and 8). Over 1–600 nM, the concentration range measured in our cell line, *k*_Obs_ scaled linearly with *c* (Fig. 4c, red circles) with a slope corresponding to the association rate constant *k*_a_ equal to (9.2±0.2) × 10^4^ M^−1^ s^−1^. Thus, the average time for a single individual TetR protein to bind to the array in a nuclear volume of ∼500 μm^3^ is on the order of 3 × 10^6^ s (that is, ∼35 days), during which it visits ∼10^5^ nonspecific sites.

### Modelling the search rate *k*_a_

Given the unexpectedly low value measured for the association rate constant, we next focused on modelling in more details the search kinetics. In our experiments, the target is composed of *N* binding sites that appear clustered within an approximately spherical locus with radius *r*_t_∼350 nm, as determined by super-resolution microscopy imaging (Fig. 4d, Supplementary Movie 9 and Supplementary Note 9). To model the association rate constant *k*_a_, we first hypothesized that the searchers perform an effective 3D diffusive motion, with a coefficient *D*=*D*_1_·*τ*_3D_/(*τ*_1D_+*τ*_3D_)=6 μm^2^ s^−1^ that takes into account the slowdown due to intermittent nonspecific binding away from the locus. For simplicity, we assumed that the binding sites (each of radius *a*=1 nm) are uniformly distributed within the sphere. The association rate constant can then be written:


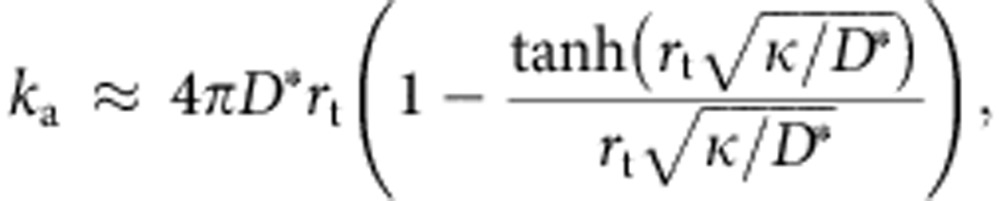


where *κ* is the probability per unit time to bind to a target sequence when the searcher is within the locus (see Supplementary Note 10). In the limit *N* ≫1, *κ* can be evaluated similarly to *k*_a_ by computing the inverse mean search time to find a target sequence of size *a* in the elementary volume 
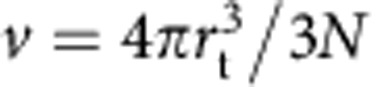
 (that is, the mean volume per target within the spherical locus), yielding:


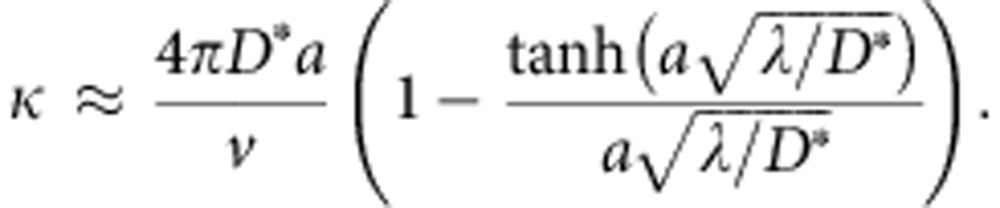


Here *λ*, which accounts for the binding efficiency, is the probability per unit time that binding occurs if a protein is within a distance *a* of a target sequence. For perfect association (that is, binding occurs at the first encounter of the protein with its target sequence), *λ* is very large (≫ *D*/*a*^2^) such that *κ* is equal to 4*πDa*/*v*. Since 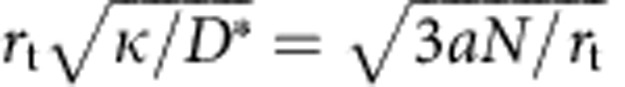
≫1, the rate *k*_a_ reduces to 4*πDr*_t_, the value for an absorbing sphere of radius *r*_t_. Given the measured values of *D* and *r*_t_, *k*_a_ should be ∼2 × 10^9^ M^−1^ s^−1^, more than four orders of magnitude larger than experimentally observed. This suggests that the binding is very inefficient at the target sites (consistently with our measurement of the nonspecific association rate 1/*τ*_3D_) and that the proteins need many encounters before stably associating to DNA. Importantly, in the low binding efficiency regime (*λ*<<*D*/*a*^2^), 
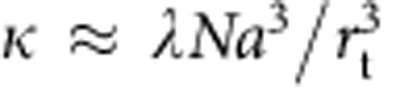
 and the association kinetics *k*_a_≈(4*π*/3)*λNa*^3^ is no longer determined by the transport properties.

To further verify that the association kinetics is reaction limited, we expressed the LacI fused to the transactivator domain VP16 (ref. 47) in U2OS 2-6-3 cells. This leads to a decondensation of the binding locus and the expression of the reporter gene CFP-SKL (Supplementary Fig. 6). The extension of the target locus increased to about 3 μm (Fig. 4d, Supplementary Movie 9 and Supplementary Note 9). Yet, this massive decompaction—and the associated modification in the large-scale organization of chromatin—had only a minimal effect on the association kinetics, which remained unchanged in most cells (Fig. 4c, brown circles). In other words, the search kinetics is largely independent of the 3D organization of the binding sites even for highly compacted conformations, which rules out a TS kinetics limited by diffusive transport in the nucleus.

The observation of nonspecific binding events in individual trajectories raised the possibility that the searchers did not reach the target site solely via 3D motion but also using a local 1D exploration at the vicinity of the *tet*O sites. To probe the potential role of 1D motion, we measured the search kinetics in the case of a different organization of the target locus using U2OS 4A cells. In this case, the target locus appeared as a ∼200nm diameter spot (Fig. 4d, Supplementary Movie 9 and Supplementary Note 9) and we measured an association rate constant equal to (2.2±0.1) × 10^4^ M^−1^ s^−1^ (Fig. 4c, blue circles), only a factor ∼4.2 lower than in U2OS 2-6-3. However, the locus occupancy at equilibrium (that is, long after Dox induction) diminished by a factor ∼90, scaling with the total number of *tet*O sites (Fig. 4e and Supplementary Note 8). In other words, the search kinetics approximately scaled with *N*_i_ the number of inserts (200 versus 30) rather than *N* the number of sites (19,200 versus 210) as would be expected for a reaction limited by the specific binding efficiency to the target. This observation means that the search process cannot be purely 3D and suggests that prior to association to specific sites, TetRs slide along the DNA flanking regions before reaching their target. As reported for LacI proteins in *E. coli*^13^, multiple binding sites behave as a single target when they are located within a genomic distance inferior to the sliding length. Our experiments indicate that the TetR sliding length exceeds the distance between *tet*O sites in each insert (23 bp both in U2OS 2-6-3 and U2OS 4A cell lines) while remaining well below the distance between inserts (∼16.5 kbp in U2OS 2-6-3 cells and ∼4 kbp in U2OS 4A cells). To evaluate the plausibility of this scenario, we estimated the sliding length 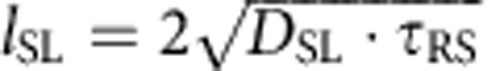 by considering a nonspecific interaction time *τ*_RS_=158 ms and assuming a 1D diffusion coefficient *D*_SL_∼10^5^–10^6^ bp^2^ s^−1^, similar to the values reported for several different DBPs diffusing along naked B-DNA^9^,^48^ and on a chromatin lattice^49^. This leads to a value of the sliding length in the range 250–750 bp, thus compatible with the experimental boundaries determined above.

To take sliding into account, we adapted the standard FD models^5^,^6^. Following our above analysis, we assumed that the limiting step of the reaction is the nonspecific binding to DNA, which is here accounted for by *τ*_3D_. Once nonspecifically attached to DNA, we hypothesized that the protein can locally scan neighbouring sequences, and eventually bind to a specific site with probability *p* if the sliding excursion overlaps with an insert. Indeed, as recently shown, proteins do not necessarily associate perfectly as they slide over their specific sites^13^. The association rate constant *k*_a_ can then be written as (ref. 6 and Supplementary Note 10):


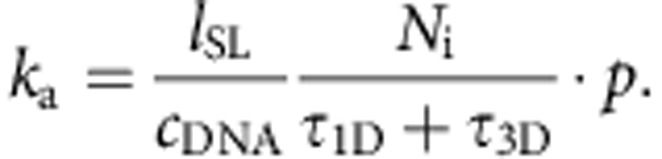


Making use of the full concentration of genomic DNA, whose limited accessibility is implicitly accounted for in *τ*_3D_, the observed value of *k*_a_ is recovered provided that *p* is close to unity (Supplementary Note 10). This suggests that, once nonspecifically attached to DNA, the protein can efficiently bind to a specific site.

## Discussion

Our experiments provide direct and quantitative insights into the search dynamics of DBPs in mammalian cells. The emerging picture for TetR is that the association to the target site proceeds in two steps (Fig. 4f). First, proteins explore the nucleus by alternating between 3D diffusion and transient association to nonspecific DNA. Yet, the nonspecific association is very inefficient possibly due to competition with nucleosomes or with other DBPs specifically or nonspecifically attached to DNA. During the transport phase, proteins spend more time diffusing in solution than engaged on nonspecific DNA interactions. This behaviour appears common for many DBPs in eukaryotic cells^17^,^18^,^22^,^50^ and contrasts with what has been reported for bacteria^12^. To further explore the generality of such a behaviour, we have investigated the behaviour of LacI proteins in mammalian cells given that, in bacteria, the LacI is predominantly bound to non-cognate DNA (87% of bound proteins) and with very short residence times^12^. FRAP experiments on NLS-LacI-GFP in U2OS 2-6-3 cells showed a fast full recovery, well described by an action-diffusion model^51^, with no stably bound molecules (Supplementary Note 11, Supplementary Movie 10). Similarly, single-particle tracking experiments on NLS-LacI-HaloTag in U2OS 2-6-3 cells showed predominantly diffusing proteins (∼80%) and a small fraction (∼20%) of proteins transiently bound to nonspecific DNA sites and with broadly distributed binding times (Supplementary Note 11, Supplementary Movie 10). The fact that the LacI in U2OS 2-6-3 cells behaves similarly to what has been reported for other DBPs (including TetR) strengthens the view that the nuclear environment of mammalian cells is crucial to control biochemical reactions. The case that most DBPs are not bound to the DNA might be the consequence of the fact that in mammalian cells DNA is decorated by a myriad of different proteins specifically or nonspecifically bound, which drastically decreases its accessibility and consequently the protein binding efficiency. Such a scenario would also explain why many transcription factors (TFs) bind to significantly fewer sites in the genome with respect to predictions based on the presence of their respective consensus motifs^52^,^53^.

At the target site, TetR proteins need to revisit the locus many times before associating to a nonspecific site in proximity of an insert. Then, in a second step, the TetR searcher locally slides until it associates to a *tet*O site. Contrarily to what happens for the LacI in bacteria, where the TS is diffusion limited^12^, for TetR in human cells the limiting rate in the search process is the association to nonspecific sites and not the diffusive transport or the sliding phase^27^.

An important—and still open—question is to determine the exact nature of the sliding movement in the local exploration phase. Because of the complex conformation of nuclear DNA and the many obstacles that could be encountered, it is possible that, during the sliding phase, the protein does not keep constant contact with DNA. Instead, its motion could involve stepping or hopping along the chromatin fibre^49^. We emphasize that movement along a low dimensional structure, even if not purely 1D, has been shown to preserve most of the kinetic properties of FD^19^. In particular, it can induce important geometric effects^54^, so that for example neighbouring target sites effectively behave as a single target, as we observe.

A significant finding is the lack of clear delimitation between specific and nonspecific binding and, instead, the observation of a continuum of association times possibly due to the variability of DNA sequences encountered in the human genome. The existence of many stable, non-functional off-targets, which might be even more pronounced for endogenous eukaryotic TFs usually having a shorter recognition sequence than TetR^16^, places constraints on the minimal number of DBPs required for overcoming the sequestration by decoy genomic sites and achieve a timely association to the target sites. Unfortunately, despite the need of having a comprehensive census of the human TFs^55^ to quantitatively study and model regulatory networks^56^, there are still very few reports on the absolute abundance of TFs in human cells. Nevertheless, multiplexed targeted proteomics has recently pointed to a huge variability in the TFs copy number (ranging from a few hundreds copies of Pias3 and ARID3a to more than 300,000 copies for NFIB) and an up to fivefold variation during cellular differentiation^57^. Indeed, such a high variability in TFs copy number might be fundamental to control the temporal response of different TFs and it will be of interest to examine in future studies to what extent the TFs copy number is a proxy of the importance of a rapid response for certain genes.

Finally, the association rate to a particular sequence can be controlled by the local arrangement of chromatin, the conformation of the protein or the association to molecular partners. A low binding efficiency, seemingly far from optimal, might become beneficial for regulatory processes. In this regard, sliding is surely advantageous compared with direct 3D association, since it transiently maintains the DBP in the vicinity of its target site, thus increasing the time available for the formation of stable regulatory complexes^18^.

## Methods

### Cellular system and culture

Experiments were conducted on human osteosarcoma (U2OS)-derived cells containing at a single locus in the genome different inserts of specific target sites. In particular, we employed U2OS 2-6-3 cells^28^, which contain 200 cassettes of 256 *lac*O and 96 *tet*O binding sites upstream of a minimal CMV promoter controlling a reporter gene (CFP-SKL) and 24 MS2 stem loops. We also used U2OS 4A cells^58^, containing 30 insertions of 7 *tet*O binding sites (Supplementary Fig. 1). In both cell lines, the 19-mers *tet*O binding sites are spaced by 23 bp, while the genomic distance between contiguous insertions is of the order of several kbp. Cells were cultured at 37 °C in the presence of 5% CO_2_ in 1 gl^−1^ glucose phenol red-free DMEM medium (11054, GIBCO, Life Technologies, USA) with 10% (v/v) fetal bovine serum (10270, GIBCO, Life Technologies, USA), 1% Pen/Strep (15140, GIBCO, Life Technologies, USA) and 1% GlutaMAX (35050, GIBCO, Life Technologies, USA). When required, cells were transiently transfected by lipid vesicles fusion (using FuGENE6, 11814443001, Roche, Swiss) 12–24 h before experiments (Supplementary Note 1, all the plasmids used in the study are available on addgene).

### Single-particle tracking experiments

TetR-Atto647N proteins diluted in PBS were microinjected in the nucleus of living cells at 1 μM in the injection needle for ensemble measurements and at 50 nM for single-particle tracking experiments. Images of TetR-Atto647N proteins have been collected using a × 150 N.A. 1.45 objective lens (UAPON 150XOTIRF, Olympus, France) and acquired with a back-illuminated Electron-Multiplied Charge-Couple-Device (EM CCD) camera (iXonEM DV860DCS-BV, Andor, Ireland) with 5 ms exposure time at a rate of 197 frames s^−1^. Typical recording were 50s long under wide-field laser illumination (Cube 640–100C, Coherent, USA) at about 0.1 kW cm^−2^. Single-molecule trajectories were generated with the MatLab (MatLab 7.0, Mathworks Inc., USA) script SLIMfast, which implements the Multiple-Target-Tracing algorithm^59^ and analysed using the script evalSPT. Data analysis routines are available on request. The localization precision achieved in the experiments was on the order of 25 nm and the longest traces recorded ranged up to few seconds (∼1,000 frames). In our experimental conditions, the measured ensemble bleaching decay time constant for TetR-Atto647N was (2.9±0.7) s or (580±140) frames (mean±s.d., *N*=5 cells). We calculated the MSD for each trajectory longer than eight frames and obtained the instantaneous diffusion coefficients *D*_Inst_ by unconstrained linear fit of the MSD curves between time lag 2 and 5 (ref. 60). The distributions of the logarithm of the instantaneous diffusion coefficient obtained for the different conditions have been fitted with a triple Gaussian function with the only purpose of estimating the characteristic value of *D*_Inst_ for the different populations and their relative abundance *f* (Supplementary Note 4).

### Nonspecific interactions analysis

We quantified the transient nonspecific interactions using a running window analysis of individual traces obtained with continuous imaging. To estimate the long nonspecific binding events, we performed time-lapse experiments acquiring snapshots (with 5 ms exposure time) with different inter-frame times (*τ*_TL_=0.1, 0.5 and 1 s) to extend the observation window to longer times while preventing photobleaching to mask the long events. We localized particle positions as for continuous imaging and we considered as immobile the proteins that remained within 1 pixel for at least two frames. Next we calculated the survival probability SP(*τ*), that is, the probability of staying bound for time longer than *τ*. The numerical integration of the SP data yielded an estimate of *τ*_1D_∼2 s. (Supplementary Note 7).

### Bioinformatics analysis

BLAST algorithm^42^ was run using the GRCh37 assembly of the 24 human chromosomes and the canonical 19bp long *tet*O sequence (TCC CTA TCA GTG ATA GAG A). To exhaustively score all sites, we increased the Expect value (E-value) until the total number of scores found by the algorithm plateaued. With an E-value of 50,000, we obtained 14,482 sites with 11 bp or longer similarity to the *tet*O sequence (Supplementary Data 1). The density of the alignments scored was approximately uniform in all chromosomes, with a mean value of almost five sites per Mbp, even though longer chromosome showed a higher density of scored sites (Supplementary Fig. 7).

### Measurement of the association rate constant *in situ*

The concentration *c* of RevTetR-GFP in each cell has been measured employing a two-photon FCS microscope^61^,^62^. In brief, we used an inverted Olympus IX81 microscope and we created an observation volume of ∼0.5 fl within the cell nucleus by focusing a tunable, mode-locked Ti:Sa laser (Chameleon Ultra II, Coherent, USA), operated at 940 nm, with a × 60 N.A. 1.2 NIR water immersion objective (UplanSApo 60XW, Olympus, Japan). The fluorescence signal was detected with a fibre-coupled (100 μm core, multi-mode fibre, AFS105/125Y, Thorlabs, USA) avalanche photo-diode (SPCM-AQRH-14-FC, Perkin-Elmer, Canada) and fed to an external digital correlator (Flex03LQ-01, Correlator.com, USA). The signal autocorrelation *G(τ)* was fitted with a purely diffusive model to determine the mean number of molecules in the excitation volume given by <*N*_Mol_>=1/*G*_(*τ*→0)_ (ref. 46). Three FCS measurements (each composed by three runs of 30s long acquisitions) were performed in three different nuclear locations to estimate the intranuclear heterogeneity of protein concentration (*x* error bars in Fig. 4c), avoiding to point on the binding site locus and on nucleoli and before inducing protein association. Association of RevTetR-GFP after Dox induction was monitored acquiring 3D stacks (usually five planes with 1μm separation) every 5 or 10 s with an intensified CCD (HQ2 CoolSNAP, Roper Scientific, Germany) with 100 ms exposure time under synchronized blue LED (M470L2, Thorlabs, USA) illumination. Binding site intensities have been quantified using ImageJ for U2OS 2-6-3 cells, and a 3D localization and intensity measuring MatLab routine (FISHquant, ref. 63) in the case of U2OS 4A cells (Supplementary Note 8).

### Super-resolution imaging of the target site

Super-resolution experiments have been conducted on an inverted microscope equipped with a perfect focus (Ti Eclipse, Nikon, France) and an adaptive optical system (MicAOTM, Imagine Optics, France) placed in the detection pathway of the microscope^64^. We used the deformable mirror (MirAO 52-e, Imagine Optics, France) to introduce an astigmatic deformation of the point spread function to obtain information on the 3D position of molecules within the focal depth of the microscope (∼600 nm, refs 64, 65). 3D PALM images have been recorded using a 561nm imaging laser (Genesis MX 561-2000 MTM, Coherent, USA) and a 405nm activation laser (Cube 405–100C, Coherent, USA) focused in the back focal plane of a × 100 N.A. 1.49 oil immersion objective (CFI Apo TIRF 100X, Nikon, France) and a 512 × 512 EM CCD (Ixon3 DU897, Andor, Ireland). Fluorescent beads (200 μm diameter, TetraspeckTM T7280, Molecular Probes, Invitrogen, USA), added on top of the cells, have been used as fiduciary markers to correct for drift during imaging. 3D super-resolution images were generated using between 80,000 and 200,000 frames acquired with an exposure time of 25 ms under continuous illumination with a density of energy of ∼4.5 kW cm^−2^ for the 561nm imaging laser and between 0 up to ∼10^2^ kW cm^−2^ for the 405nm activation laser (Supplementary Note 9). Super-resolution images have been reconstructed with dedicated MatLab routines (available on request), while images rendering and quantification (Fig. 4d and Supplementary Movie 9) have been done using the software ViSP^66^.

## Additional information

**How to cite this article:** Normanno, D. *et al*. Probing the target search of DNA-binding proteins in mammalian cells using TetR as model searcher. *Nat. Commun.* 6:7357 doi: 10.1038/ncomms8357 (2015).

## Supplementary Material

Supplementary Information Supplementary Figures 1-35, Supplementary Tables 1-13, Supplementary Notes 1-11 and Supplementary References.

Supplementary Movie 1 TetR-Atto647N micro-injection and binding to the target site

Supplementary Movie 2 Dox-induced release from the target site

Supplementary Movie 3 Single-Particle-Tracking experiments

Supplementary Movie 4 Individual trajectories analysis

Supplementary Movie 5 sptPALM experiments

Supplementary Movie 6 FRAP experiments

Supplementary Movie 7 Transient nonspecific binding events in individual trajectories

Supplementary Movie 8 RevTetR-GFP induction experiments

Supplementary Movie 9 Super-resolution images of the target sites

Supplementary Movie 10 LacI behavior in mammalian cells

Supplementary Data 1 BLAST alignment of tetO to human genome results list
