# Supplementary material for: Probing the target search of DNA-binding proteins in mammalian cells using TetR as model searcher
Source: Nat Commun. 2015 Jul 7;6:7357. doi: 10.1038/ncomms8357 (PMC4507003; doi:10.1038/ncomms8357)
Supplement: Supplementary Information — Supplementary Figures 1-35, Supplementary Tables 1-13, Supplementary Notes 1-11 and Supplementary References. [file ncomms8357-s1.pdf]

Supplementary Information for

Probing the target search of DNA-binding proteins in mammalian cells using TetR as model searcher

by

Davide Normanno, Lydia Boudarène, Claire Dugast-Darzacq, Jiji Chen, Christian Richter, Florence Proux, Olivier Bénichou, Raphaël Voituriez, Xavier Darzacq, and Maxime Dahan

correspondence to:

davide.normanno@inserm.fr, darzacq@berkeley.edu, maxime.dahan@curie.fr

This PDF file includes:

Supplementary Figures 1 to 7

Supplementary Notes 1 to 11

Supplementary References 1 to 56

Other on-line Supplementary Information for this manuscript includes:

Supplementary Movies 1 to 10

Supplementary Data 1

Content

Supplementary Figures

Supplementary Fig. 1: Binding site loci architecture... 3
Supplementary Fig. 2: Confined diffusion analysis.. 4
Supplementary Fig. 3: Running windows analysis in the different experimental conditions. 6
Supplementary Fig. 4: Unlabeled TetR and TetR-Atto647N association and dissociation rates *in vitro*.. 7
Supplementary Fig. 5: RevTetR-GFP association kinetics at different Dox concentrations.. 8
Supplementary Fig. 6: Transcription activation of the artificial gene array with NLS-LacI-mCherry-VP16...9
Supplementary Fig. 7: BLAST alignment of *tet*O to the human genome..................... 10

Supplementary Notes

Supplementary Note 1: Protein constructs and cell lines... 11
Supplementary Note 2: TetR purification, characterization, and micro-injection................ 15
Supplementary Note 3: TetR-GFP assay...26
Supplementary Note 4: Single-Particle-Tracking experiments and analysis......................27
Supplementary Note 5: TetR/Rev-TetR mobility analysis by sptPALM...........................35
Supplementary Note 6: TetR-GFP FRAP experiments...39
Supplementary Note 7: Analysis of transient interactions with nonspecific DNA................ 43
Supplementary Note 8: Measurement of the association rate constant *in situ*..................53
Supplementary Note 9: Super-resolution imaging of the target site..............................58
Supplementary Note 10: Modeling of the search kinetics... 60
Supplementary Note 11: LacI behavior in mammalian cells... 64

Supplementary References.. 70

Supplementary Fig. 1

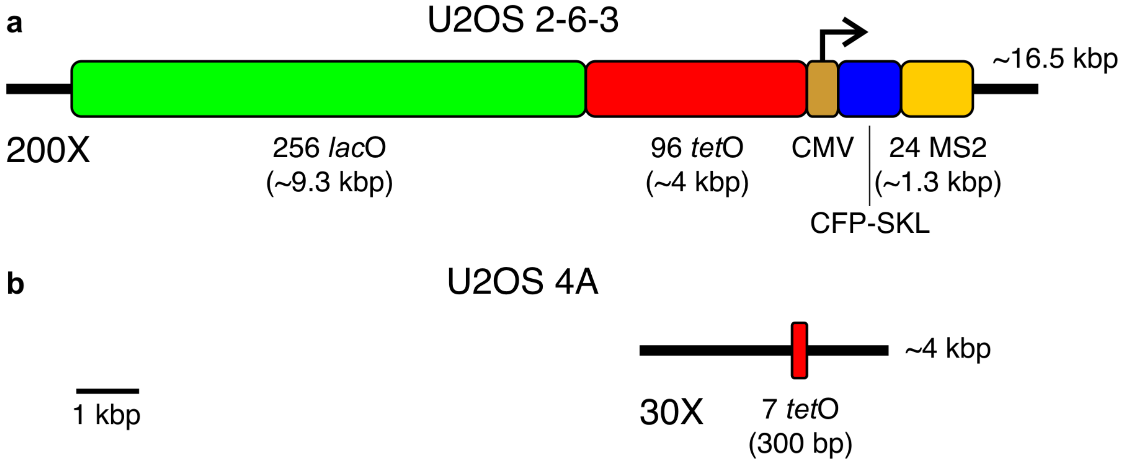

Supplementary Fig. 1 – Binding site loci architecture

(**a**) Binding site locus of the U2OS 2-6-3 cell line (1). It consists of 200 insertions at a unique genomic site of a gene array containing 256 *lacO* binding sites, 96 *tetO* binding sites, a minimal CMV promoter, a reporter gene encoding for CFP-SKL, and 24 MS2 stem loops (16.5 kpb). (**b**) Binding site locus of the U2OS 4A cell line (2,3) containing at a single locus 30 insertions of 7 *tet*O binding sites inserted in a 4 kbp-long cassette. Scale bar 1 kpb, drawing to scale.

Supplementary Fig. 2

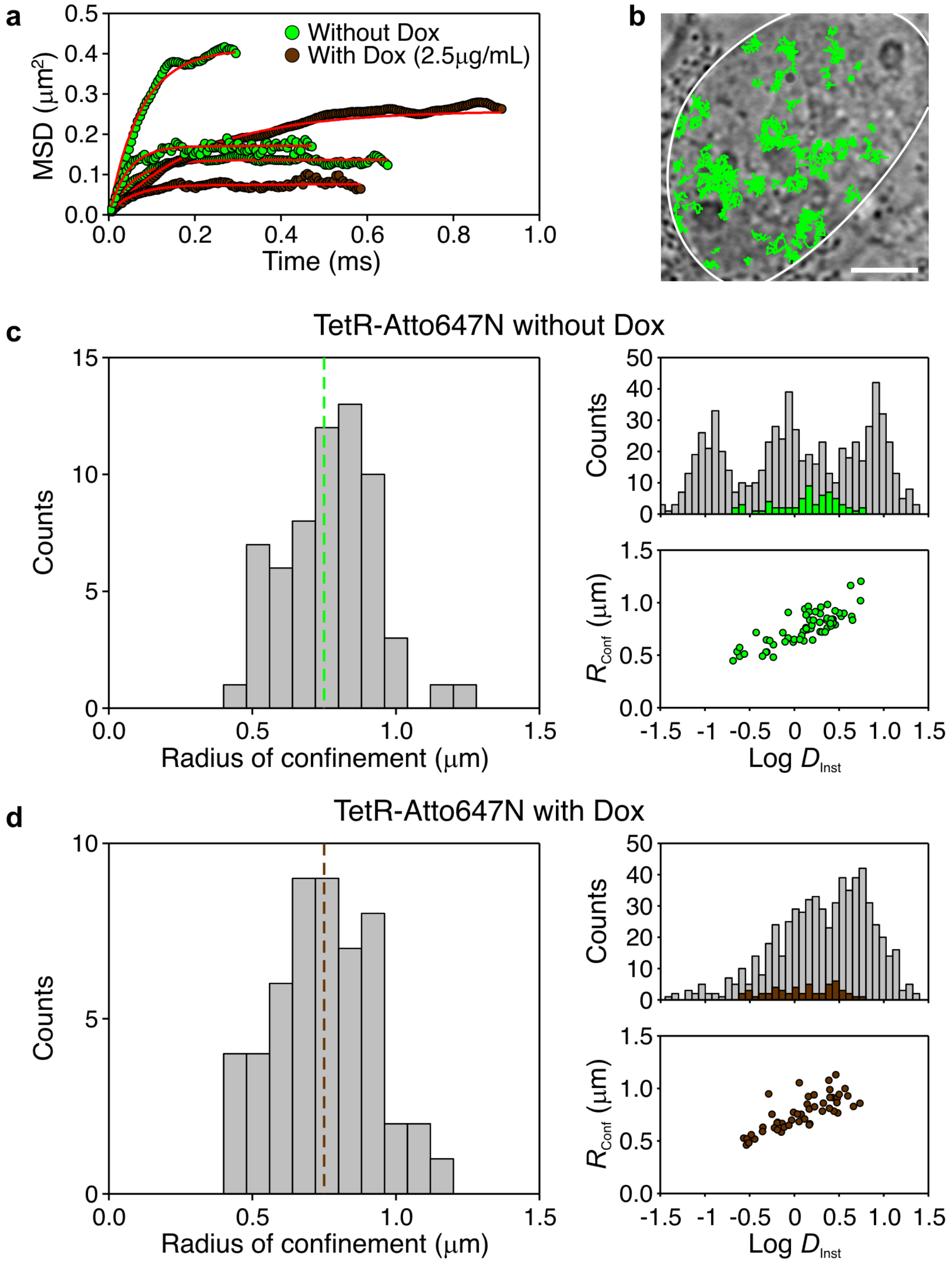

Supplementary Fig. 2 – Confined diffusion analysis

(**a**) MSD *vs.* time curves showing confined diffusion of proteins belonging to the intermediate population in the absence (green circles) and in the presence (brown circles) of Dox (2.5 μg per ml). Red lines are best fits to the data with a confined diffusion model (Equation 1, Supplementary Note 4). (**b**) Example of confined trajectories (in the absence of Dox) superimposed to the transmission image

of the nucleus of the cell. Scale bar 5 µm. (**c**) Distribution of the radius of confinement R_{Conf} in the absence of Dox (mean ± s.d. = 0.77 ± 0.02 µm). The subset of trajectories (belonging to the intermediate population) and showing confined diffusion is shown in green (n = 62) over the total population (grey bars, n = 682, N = 10 cells)) in the top right histogram, the correlation between R_{Conf} and D_{Inst} is shown in the scatter plot at the bottom right. (**d**) Distribution of the radius of confinement R_{Conf} in the presence of Dox (2.5 µg per ml) of Dox (mean ± s.d. = 0.76 ± 0.02 µm). The subset of trajectories (belonging to the intermediate population) and showing confined diffusion is shown in brown (n = 49) over the total population (grey bars, n = 623, N = 8 cells) in the top right histogram, the correlation between R_{Conf} and D_{Inst} is shown in the scatter plot at the bottom right.

Supplementary Fig. 3

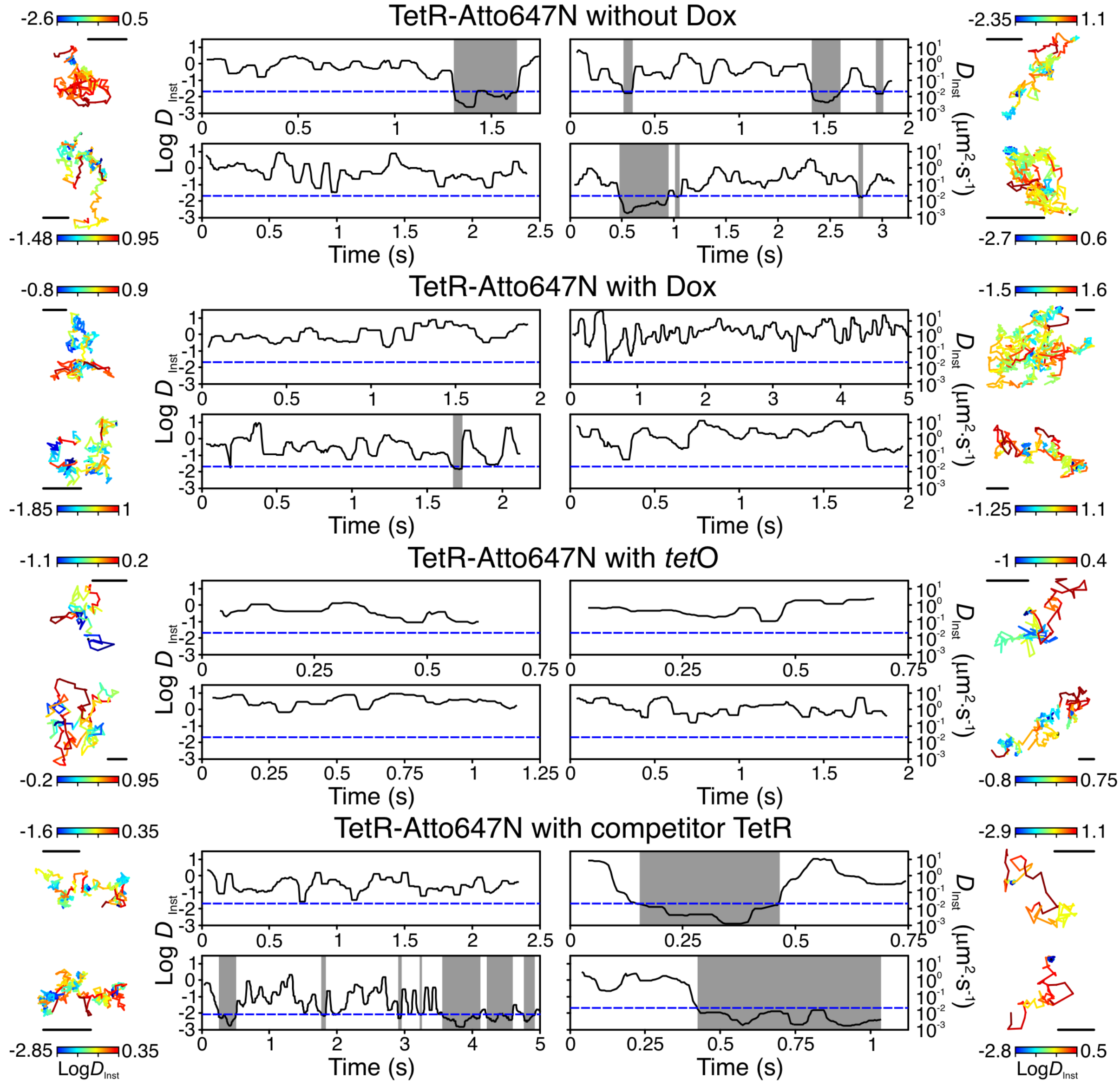

Supplementary Fig. 3 – Running window analysis in the different experimental conditions

The figure shows the results of the running window analysis for 4 different individual TetR-Atto647N trajectories in each of the different experimental conditions tested. From top to bottom: in the absence of Dox - basal conditions; in the presence of Dox at 2.5 µg per ml final concentration; upon *tet*O (10X molar excess) co-injection; and in the case of 1000 folds excess of competitor unlabeled TetR proteins. The time course of D_{Inst} has been computed with a running window of 80 ms (16 frames) and is displayed in logarithmic scale (Log D_{Inst}), grey boxes indicate non-specific binding events detected with threshold (blue dashed line) analysis (Supplementary Note 7). Individual trajectories (colored lines) have been color-coded according to the Log D_{Inst} values (color bars). Scale bars 1 µm.

Supplementary Fig. 4

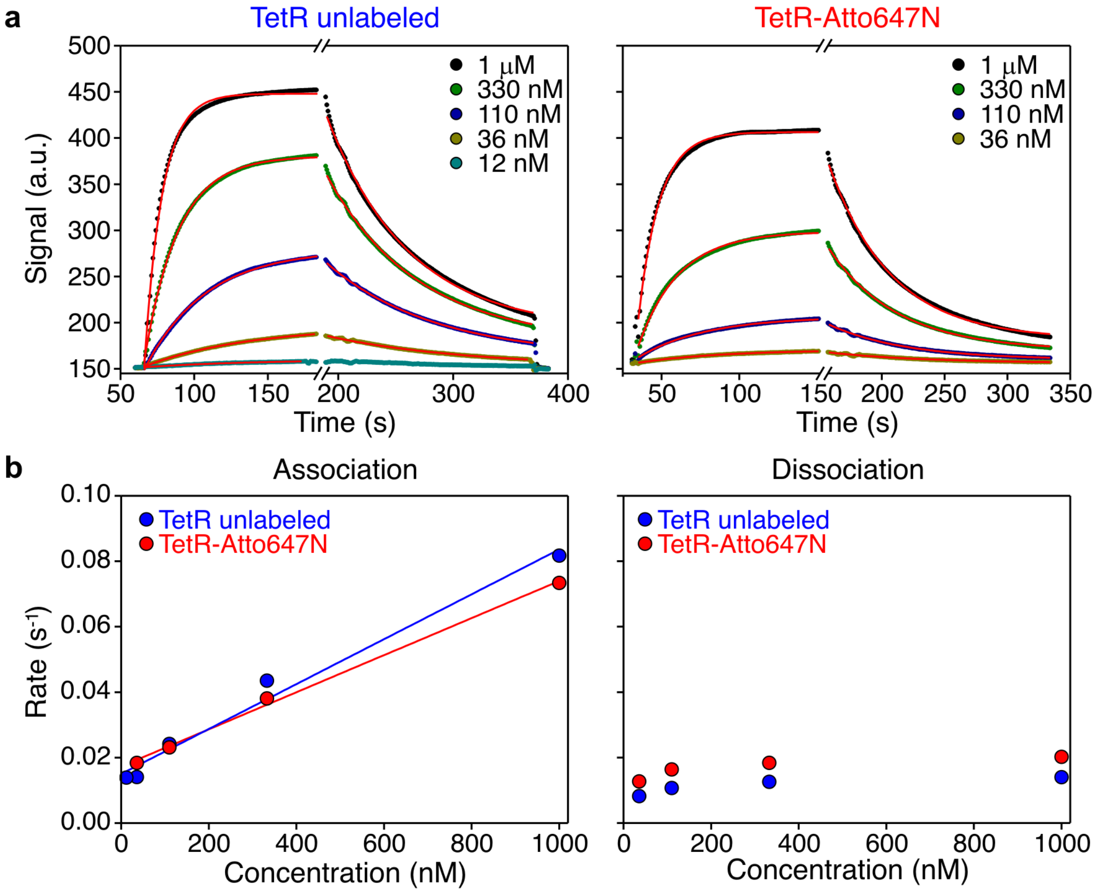

Supplementary Fig. 4 – Unlabeled TetR and TetR-Atto647N association and dissociation rates *in vitro*

(**a**) Sensorgrams *vs.* time recorded with a Biacore assay. Left, unlabeled TetR; and right, TetR-Atto647N. (**b**) Left, association; and right, dissociation rate as a function of protein concentration for the unlabeled TetR (blue circles) and for the TetR-Atto647N (red circles). Error bars fall within symbol dimensions. Linear fits (blue and red lines in the left panel) of the association rate values furnished an association rate constant of $(6.8 \pm 0.8)\cdot 10^4$ $M^{-1}\cdot s^{-1}$ for TetR unlabeled and of $(5.7 \pm 0.4)\cdot 10^4$ $M^{-1}\cdot s^{-1}$ for TetR-Atto647N. The estimated dissociation rates are (0.015 ± 0.002) s^{-1} for TetR unlabeled and (0.017 ± 0.001) s^{-1} for TetR-Atto647N, which corresponds to a characteristic binding time of TetRs to *tet*O (τ_{SPE}) of about 60s.

Supplementary Fig. 5

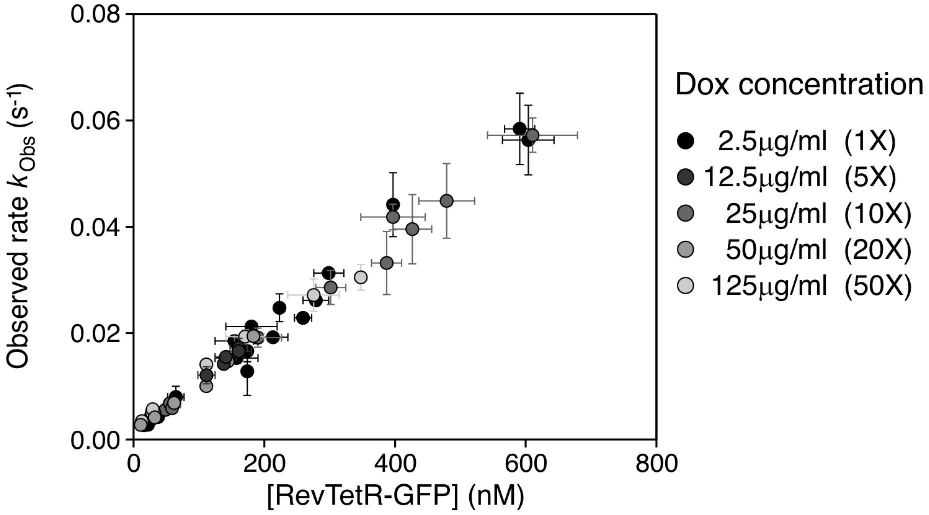

Supplementary Fig. 5 – RevTetR-GFP association kinetics at different Dox concentrations

The figure shows the observed association rate for different U2OS 2-6-3 cells as a function of the concentration of RevTetR-GFP in the nucleus for experiments performed at different concentration of Dox (from 2.5 up to 125 µg per ml final concentration). All the data display the same behavior, which suggests that Dox permeation with the cell nucleus is not playing a role in the association kinetics studied. Error bars represent s.d. and when not visible fall within symbol dimensions.

Supplementary Fig. 6

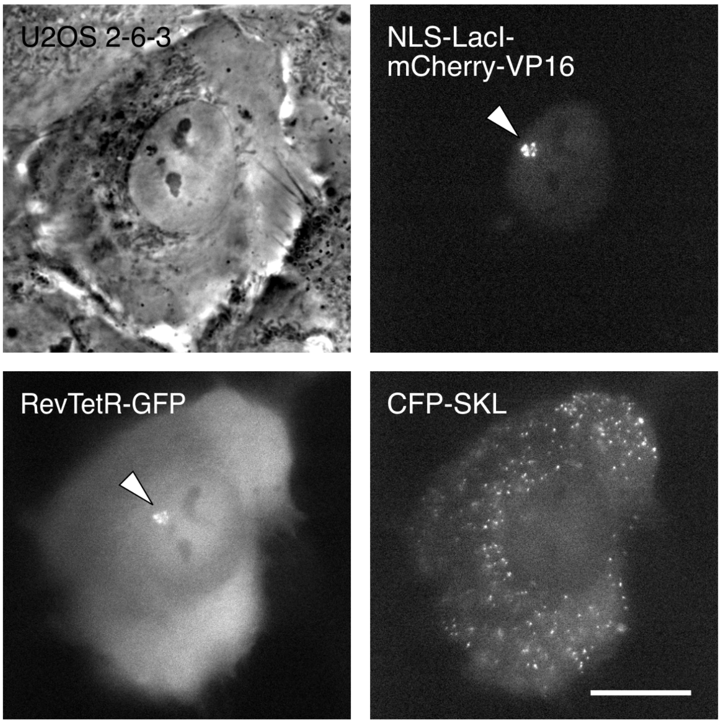

Supplementary Fig. 6 – Transcription activation of the artificial gene array with NLS-LacI-mCherry-VP16

The figure shows how transcription of the artificial gene array of the U2OS 2-6-3 cells can be activated using the VP16 trans-activator domain (4) fused to the LacI protein. The top left panel shows a phase image of a U2OS 2-6-3 cell stably expressing RevTetR-GFP. After Dox induction (2.5 μg per ml final concentration), RevTetR-GFP is recruited at the gene array (white arrow, bottom left panel), where also the NLS-LacI-mCherry-VP16 construct is accumulated (white arrow, top right panel) activating the transcription of the CFP-SKL reporter gene (bottom right panel) placed downstream the binding sites (Ref. 1, see also Supplementary Fig. 1). Scale bar 10 μm.

Supplementary Fig. 7

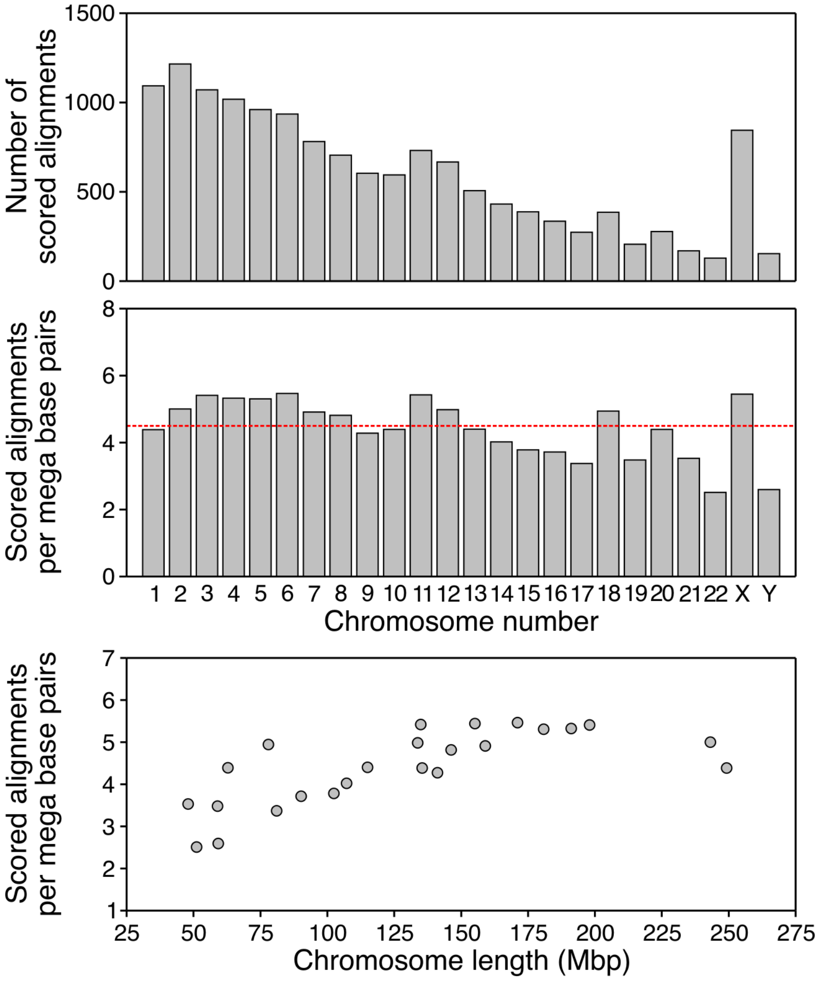

Supplementary Fig. 7 – BLAST alignment of *tet*O to the human genome

The top panel shows the total number of alignments, with 11 bp or longer similarity to the *tet*O sequence, found in the 24 human chromosomes (GRCh37 assembly) using the BLAST algorithm with an E-value of 50,000 (Supplementary Data 1). The middle panel represents the density of scored alignments (number of alignments with 11 bp or longer similarity to *tet*O per mega base pairs) for the 24 human chromosomes. The dashed red line indicates the mean value (4.7 alignments per mega base pairs). The bottom panel displays the correlation plot (grey circles) between the density of scored alignments and the chromosome length. Longer chromosomes have higher densities of alignments with 11 bp or longer similarity to the *tet*O sequence (Pearson correlation coefficient: 0.70).

Supplementary Note 1

Protein constructs and cell lines

TetR and RevTetR coding constructs and expression vectors

All experiments of this study have been conducted on engineered proteins derived from two variants of the bacterial tetracycline repressor class B (5) (TetR(B), Genebank accession number J01830.1, kindly provided by Prof. Wolfgang Hillen, Friedrich-Alexander-Universität Erlangen-Nürnberg, Erlangen, Germany) and are summarized in Supplementary Table 1.

TetR(B) recognizes and specifically binds to a 19bp-long DNA sequence, named *tet* operator or *tet*O (TCC CTA TCA GTG ATA GAG A) (6). TetR affinity for *tet*O is strongly controlled by tetracycline (Tc) and several Tc analogs such as doxycycline (Dox) (7). In the presence of saturating Dox, TetR(B) affinity for *tet*O drops by 9 orders of magnitude (8). The first TetR(B) variant we used contains a single cysteine for site-specific labeling (we refer to this variant as TetR). In particular, we kept cysteine C203 and we performed the following substitution: C68S, C88N, C121T, C144S, and C195S, with amino acids either with the same chemical properties or found at the same residues in other TetRs variants. Notably, a variant of TetR without all the six natural cysteines was already shown to be functional and to present similar features in its activity to TetR(B) when its crystal structure had been solved (9). The second variant used in this study has 5 point mutations (S12G, E19G, A56P, D148E, and H179R) compared to the single cysteine TetR. It presents low affinity for DNA in the absence of Tc or other Tc analogs, and high affinity for DNA in the presence of Tc, thus it is named reverse *tet* repressor (RevTetR) (10,11). An ACC codon was added in 5' immediately before the translation initiation codon to establish a Kozak sequence needed for correct expression in eukaryotic cells. A TEV cleavage site and a HIS tag (*i.e.* a stretch of six histidines) were added at the end of the coding sequence for protein purification purposes. The modified versions of TetR and RevTetR cDNA sequences described earlier have been synthesized (GenScript Co., USA) and cloned in the pUC57 vector. Both TetR and RevTetR cDNA have been cloned by NheI/KpnI digestion/ligation process in the mammalian expression vectors pE-GFP-N1 (6085-1, Clontech, USA) and pDendra2-N1 (12). The NheI is located in 5' of the Kozak sequence to ensure its transfer into the mammalian expression vectors. The KpnI site is located in 5' of the TEV and HIS sequences in order not to bring the TEV site and the HIS tag in the mammalian expression vector and KpnI is also positioned in frame with the GFP or the Dendra2 coding sequence in their respective cloning vectors to ensure in frame cloning of the constructs with GFP or Dendra2. TetR has been cloned also in the bacterial expression vector pWH610 (kindly provided by Prof. Wolfgang Hillen, Friedrich-Alexander-Universität Erlangen-Nürnberg, Erlangen, Germany) via XbaI/BstEI digestion/ligation process in order to properly transfer the cDNA from the TCT second translated codon to the end of the HIS tag (after the Stop translation codon), being the translation initiation ATG codon already present in the pWH610 vector.

LacI constructs

In addition to the different TetR constructs, we used also different fusions of the LacI protein (Supplementary Table 1) in order to visualize and/or manipulate the genomic locus containing the binding sites. In particular we used NLS-LacI-GFP, NLS-LacI-YFP, and NLS-LacI-RFP (a generous gift of Prof. Robert H. Singer, Albert Einstein College of Medicine, New York City, USA) (13), and NLS-LacI-GFP-VP16 (kindly provided by Prof. Andrew S. Belmont, University of Illinois at Urbana-Champaign, USA) (4). This latter construct was also sub-cloned replacing the GFP with mCherry in order to obtain the NLS-LacI-mCherry-VP16 construct.

TetR constructs	LacI constructs
TetR*	NLS-LacI-GFP
TetR-GFP	NLS-LacI-YFP
TetR-Dendra2	NLS-LacI-RFP
RevTetR-GFP	NLS-LacI-HaloTag
Rev-TetR-Dendra2	NLS-LacI-mCherry-VP16

Supplementary Table 1 – TetR and LacI constructs

The table shows the list of the different protein-coding plasmids used in the study.

*For bacterial expression

Cell lines and the genomic insertions of specific binding sites

Different derivates of the human osteosarcoma cell line (U2OS) have been used for experiments. In particular, we employed the U2OS 2-6-3 cell line (1), which contains 200 insertions at a unique genomic site of 256 *lacO* binding sites, 96 *tetO* binding sites, a minimal CMV promoter, a reporter gene encoding for CFP-SKL, and 24 MS2 stem loops (Supplementary Fig. 1a). We also used U2OS 4A cells (2,3), containing at a single locus 30 insertions of 7 *tet*O binding sites (Supplementary Fig. 1b). For both cell lines, the *tet*O binding sites have been cloned from the plasmid pTRE2 (Clontech, USA, Ref. 14). The Tet Response Element (TRE) of the pTRE2 plasmid is 294 bp long and contains seven *tetO* 19-mers located at positions 15-33, 57-75, 99-117, 141-159, 183-201, 225-243, and 267-285 with an intervening distance between contiguous *tet*O sites of 23bp (see Supplementary Table 2). In the U2OS 2-6-3 cell line, 16 cassettes of the first six *tet*O repeats of the TRE have been tandemly joined to obtain the 96 *tet*O cassette (15). The genomic distance between contiguous insertions (200 for the U2OS 2-6-3 and 30 for the U2OS 4A) is respectively of the order of 16.5 and 4 kbp.

**0**	CTCGAGTTTA	CCAC**TCCCTA**	**TCAGTGATAG**	**AGA**AAAGTGA	AAGTCGAGTT	**50**
**51**	TACCAC**TCCC**	**TATCAGTGAT**	**AGAGA**AAAGT	GAAAGTCGAG	TTTACCAC**TC**	**100**
**101**	**CCTATCAGTG**	**ATAGAGA**AAA	GTGAAAGTCG	AGTTTACCAC	**TCCCTATCAG**	**150**
**151**	**TGATAGAGA**A	AAGTGAAAGT	CGAGTTTACCA	C**TCCCTATCA**	**GTGATAGAGA**	**200**
**201**	AAAGTGAAAG	TCGAGTTTAC	CAC**TCCCTAT**	**CAGTGATAGA**	**GA**AAAGTGAA	**250**
**251**	AGTCGAGTTT	ACCAC**TCCCT**	**ATCAGTGATA**	**GAGA**AAAGTG	AAAGTCGAGC	**300**

Supplementary Table 2 – Tet Response Element

The table reports the first 300 bases of the pTRE2 plasmid (Clontech, CA) (14). The 7 *tet*O sites are shown in bold.

Cell culturing

Cells were cultured at 37°C in a humidity saturated environment in the presence of 5% CO_2 in low-glucose (1 g per L) phenol red–free DMEM medium (11054 GIBCO, Life Technologies, USA), supplemented with 10% (v/v) FBS (10270 GIBCO, Life Technologies, USA), 1% Pen/Strep (15140 GIBCO, Life Technologies, USA), and 1% GlutaMAX™ (35050 GIBCO, Life Technologies, USA). Every 2 to 3 days, cells were detached using Trypsin EDTA (15400 GIBCO, Life Technologies, USA) and diluted from 80% to 20% confluence, also cells were regularly tested for mycoplasma contaminations with the MycoScope PCR Detection Kit (MY01050, Genlantis, USA).

Cell transfection

When required for experiments, cells were plated on coverslips over-night and then transfected for 12-24 h by lipid vesicles fusion (using FuGENE6, purchased either from Roche, Swiss - 11814443001, or from Promega, France - E2693) according to the manufacturer protocol. In particular, per each 35 mm well, we used 0.1-0.2 μg of the desired expression vector and 0.8-0.9 μg of a carrier plasmid without eukaryotic promoters (pSP64 Poly(A) Vector, P1241 Promega, France), which was used to increase transfection efficiency while keeping a moderate expression level of the desired protein.

Stable cell lines

From the U2OS 2-6-3 cell line, we derived clonal sub-cell lines stably expressing TetR-Dendra2, Rev-TetR-Dendra2, and Rev-TetR-GFP. To obtain the stable sub-cell lines, we transfected 80% confluent U2OS 2-6-3 cells (plated in a 10 cm Petri dish) with 1 to 6 μg of the desired plasmids using FuGENE6 (11814443001, Roche, Swiss). Two days after transfection, we started selecting cells for two weeks with the antibiotic Geneticin (1 mg per ml) (10131027 GIBCO, Life Technologies, USA). Single clone colonies have been then detached with Accutase (L11-007, PAA, USA) using glass cylinders and amplified in plates of increasing size (from 48-well plates to 10 cm Petri dishes) always under Geneticin selection. TetR-Dendra2- and Rev-TetR-Dendra2-expressing cells, given the requirement of a very low expression level, were selected by FACS sorting (ARIA III, BD Bioscience, USA).

Live-cell experiments

Live-cell experiments were performed at 37°C on post-mitotic cells plated on plasma-cleaned glass coverslips (ϕ = 25 mm, #1, Menzel-Gläser, Germany), or on MatTek dishes (P35G-0.17-14-C-Case, MatTek Corporation, USA), in pH-stabilized phenol red–free Leibovitz's (L15) medium (21083 GIBCO, Life Technologies, USA) containing 10% (v/v) FBS and 1% Pen/Strep. In sptPALM experiment, cells were plated on plasma-cleaned coverslips coated with type I collagen (A 10483-01, GIBCO, Life Technologies, USA). Experiments involving TetR and RevTetR mobility in the presence of Dox (D9891, Sigma-Aldrich, USA) were conducted after incubating cells for at least 10 minutes with L15 medium containing freshly added Dox at a final concentration of 2.5 μg per ml. TetR-GFP release from the binding site locus and RevTetR-GFP association to the binding site locus experiments have been performed by rapidly adding 1 ml of warm L15 medium containing freshly added Dox at the double of the desired final concentration into 1 ml of cell medium.

Supplementary Note 2

TetR purification, characterization and micro-injection

TetR expression and purification

We used thermal shock to transform RB791 *E. coli* cells (kindly provided by Prof. Wolfgang Hillen, Friedrich-Alexander-Universität Erlangen-Nürnberg, Erlangen, Germany) with the pWH610 vector containing the single cysteine TetR with the TEV site and the HIS tag at the C terminus of the protein. Starting from a single bacterial colony, we grew competent *E. coli* cells at 37°C in LB under Ampicillin selection (50 μg per ml). At optical density ~ 0.6, we induced TetR over-expression for 180 minutes with IPTG (0.5 mM). Afterwards, we harvested cells via centrifugation and froze the cell pellet overnight prior to lysis. Next, cell were re-suspended in LBB buffer at 4°C and then lysed via sonication (Ultrasonic Processor, model GE600, 600W 20kHz, probe #V1A, Sonics & Materials, USA) in 4 rounds (20% power, duty cycle 90%, 30 seconds per round, 5 minutes between rounds, sample kept in wet ice). Subsequently, the cell lysate have been ultra-centrifugated for 20 min at 10,000 rpm, at 4°C to clear cell debris and the supernatant kept for purification. For a first purification step, we used Ni-NTA resin column (Ni-NTA Agarose R901-15, Invitrogen) to capture TetR via the HIS tag inserted at the N terminal domain of the protein. We first equilibrated the resin by a few round of washing (by centrifugation) with LBB buffer, and then we incubated the resin together with the cleared cell lysate for 60 minutes at 10 rpm at 4°C. Next, we loaded the resin incubated with the protein on a column, we waited for the resin settling down (15 minutes), we extensively washed the resin with 200 ml of LBB buffer, and then we eluted the TetR by washing the resin with 25 ml of elution buffer. The flow-through has been collected in 1 ml fractions then analyzed on Wattman paper via Coomassie Blue staining. The most concentrated fractions have been mixed together and kept for further purification steps and labeling. Next, in order to remove the HIS tag used for the first round of purification, we used a TEV cleavage site inserted between the HIS tag and the core of the protein. First, TetR were dialyzed overnight in 10 kDa membranes against TEV Cleavage Buffer. Following, TetR were digested with TEV protease (AcTEV Protease 12575, Invitrogen, USA) for 180 minutes, 350 rpm, at 16°C. Next, we used a new Ni-NTA resin column to capture the HIS tag cleaved out from the TetR and the TEV protease, which also carries a HIS tag for removal purposes. This time, after washing and equilibrating the resin, we incubated it with the TEV digestion product for 60 minutes, at 10 rpm, at 4°C. We let it settle down (~15 min) and then we collected the flow-through corresponding to the TetR purified and with the HIS tag cleaved. Importantly, the only difference between the purified TetR protein and the TetR-GFP/TetR-Dendra2 construct for mammalian expression is in the linker region after the TetR cDNA (see Supplementary Table 3). The result of TetR purification has been first inspected by SDS gel separation. Line 4 in the gel shows the purified, cleaved TetR, which has a single clean band around 23 kDa, the M.W. of the monomeric TetR (Supplementary Fig. 8a).

Mammalian expression	Kozak – ATG – TetR cDNA – AA209 – **Arg-Ala-Asp-Pro-Pro-Val-Ala-Thr** – GFP/Dendra2
Bacterial expression	ATG – TetR cDNA – AA209 – **Asp-Glu-Leu-Pro-Arg** – TEV – HIS

Supplementary Table 3 – TetR-GFP/Dendra2 and TetR-Atto647N constructs comparison
The table shows the terminal sequence of the different TetR protein constructs used for endogenous expression or for purification and micro-injection.

TetR labeling and storing

After purification, a fraction of TetR has been dialyzed against the storage buffer (ZAP 1X with 50% glycerol) and stored at -20°C. The remaining purified protein has been dialyzed against PBS and then labeled with the organic dyes: Atto647N (AD 647N-41, Atto-Tec GmbH, Germany), Atto655 (Ad 655-41, Atto-Tec GmbH, Germany), and Cy5 (PA15131, GE Healthcare, USA). Protein (covalent) labeling has been achieved by direct reaction between the single cysteine of the TetR and a maleimide reactive group on the dyes. In particular, we first incubated the TetR with 0.5 mM TCEP (646547, Sigma-Aldrich, USA) for 180 minutes shacking at 450 rpm at R.T. in the dark. Then, we added 50 to 1 excess of dyes freshly diluted in freshly opened DMSO and we incubated for 180 minutes shacking at 450 rpm at R.T. in the dark. Finally, we added β-Mercaptoethanol (1.43 M final concentration) and incubated for 60 minutes at 450 rpm at R.T. in the dark.

After labeling, the uncoupled dyes have been removed from the labeled protein first with G-50 resin spin-down columns (Illustra MicroSpin G-50 Columns 27-5330-01, GE Healthcare, USA), then with repeated washing with 10kDa cutoff spin-down columns (VivaSpin500 VS0102, Sartorius, Germany). Once the flow through of the cutoff spin-down column did not presented any residual florescence in a NanoDrop assay, the purified and the protein have been dialyzed for 24 h against ZAP buffer 1X containing 50% glycerol and stored in mono-use aliquots at -20°C. Protein activity has shown no losses over about three years.

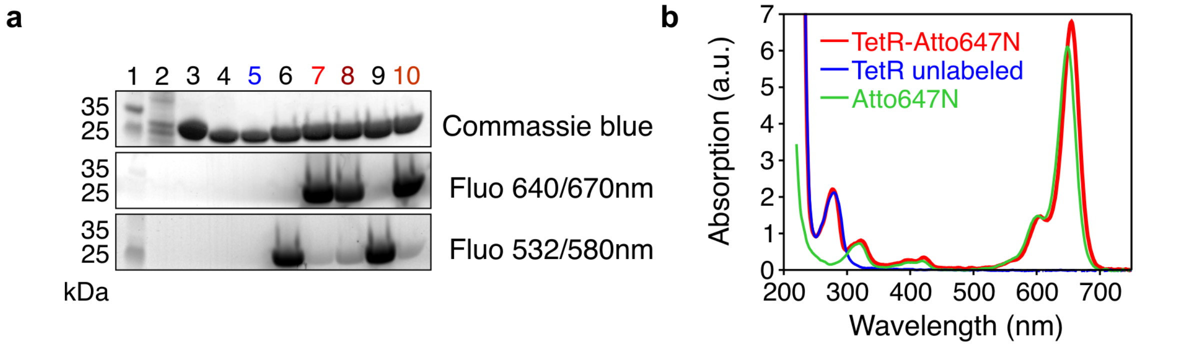

c

TetR	Dye structure	charge	ε (L·mol^{-1}·cm^{-1})	[Dye] (μM)	[TetR] (μM)	D.O.L.	Dyes per TetR dimer
Unlabeled			15940		273		
Atto647N	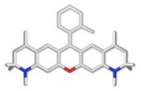	+	150000	60	80	0.75	
Atto655		N	125000	45	84	0.54	
Cy5	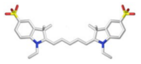	–	250000	42	67	0.63	

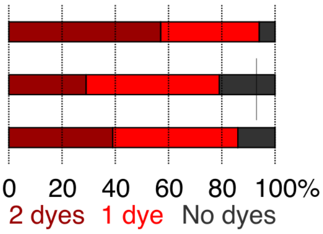

Supplementary Fig. 8 – TetR purification, labeling and characterization

(**a**) SDS 10%-Polyacrylamide gel showing: 1, Protein ladder – 2, Cell lysate – 3, TetR after Ni-NTA resin purification – 4, TetR after TEV cleavage – 5, TetR purified unlabeled – 6, TetR purified labeled with Atto532 – 7, TetR purified labeled with Atto647N – 8, TetR purified labeled with Atto655 – 9, TetR purified labeled with Cy3 – 10, TetR purified labeled with Cy5. First line: Coomassie Blue staining; second line: fluorescence scan (Fluorescent Image Analyzer FLA-3000, FujiFilm) excitation 640 nm - emission 670 nm; third line: fluorescence scan excitation 532 nm - emission 580 nm. (**b**) The figure shows the absorption spectra recorded with a NanoDrop for the labeled protein (TetR-Atto647N, red line), for the pure protein (TetR, blue line), and for the dye (Atto647N, green line). (**c**) The table summarizes and compares the properties of the different labeled TetR constructs. The first line refers to the unlabeled TetR. The second column shows the chemical structure and the charge, and the third the extinction coefficient of the different dyes used. The last four columns report the concentrations of protein and dye measured by NanoDrop inspection, the resulting Degree Of Labeling (D.O.L.), and the corresponding fraction of TetR dimers carrying two (dark red), one (red) or zero (dark grey) dye.

Buffers employed for TetR purification

Lysis-Binding Buffer (LBB) 1X

20 mM	Tris pH8.8
0.5 M	NaCl
5 mM	Imidazole
10%	Glycerol
10 mM	β-Merkaptoethanol
10 g per L	Betaine
	MilliQ H_20

Stored at 4°C

Elution Buffer 1X

20 mM	Tris pH8.8
0.5 M	NaCl
250 mM	Imidazole
10%	Glycerol
10 mM	β -Merkaptoethanol
10 g per L	Betaine
	MilliQ H_20

Stored at 4°C

TEV Cleavage Buffer 10X

10 mM	Tris pH8
150 mM	NaCl
0.5 mM	EDTA
0.1% v/v	NP40
1 mM	DTT
	MilliQ H_2O

Stored at 4°C

ZAP 10X – 200mL:

10 mM	Tris pH8
2 M	NaCl
	MilliQ H_2O

Filtered with 0.2μm syringe filters and stored at 4°C

TetR storage buffer:

20 ml	ZAP 10X
100 ml	Glycerol 100%

Sterilized by autoclaving. Stored at 4°C. Mix 2 volumes of proteins to 3 volumes of storage buffer.

TetR characterization

The purified (labeled and unlabeled) TetR proteins have been extensively characterized in standard *in vitro* biochemical assays. In particular, we determined the Degree Of Labeling (D.O.L.) of the proteins by absorbance measurements (Supplementary Fig. 8b) with NanoDrop (NanoDrop 2000, Thermo Scientific, USA). Dye concentrations have been directly obtained by measuring the absorbance and considering the extinction coefficients of the dyes reported in Supplementary Fig. 8c. Protein concentration has been obtained after correcting for the dye absorption at 280 nm and considering the TetR extinction coefficient as 15940 L·mol^{-1}·cm^{-1}. Then, the D.O.L. of the labeled proteins has been determined as the ratio between the (monomeric) TetR concentration and the dye concentration (Supplementary Fig. 8c).

Next, we performed diagnostic HPLC (1200 Series, Agilent Technologies, France) to check for the presence of free dye in the protein final preparations. We could detect no residual free dye in the TetR labeled samples: labeled TetR was exiting the column at about 16 minutes while the free dye exited from the column only at around 46 minutes and the TetR-Atto647N trace did not show any detectable presence of free dye (Supplementary Fig. 9).

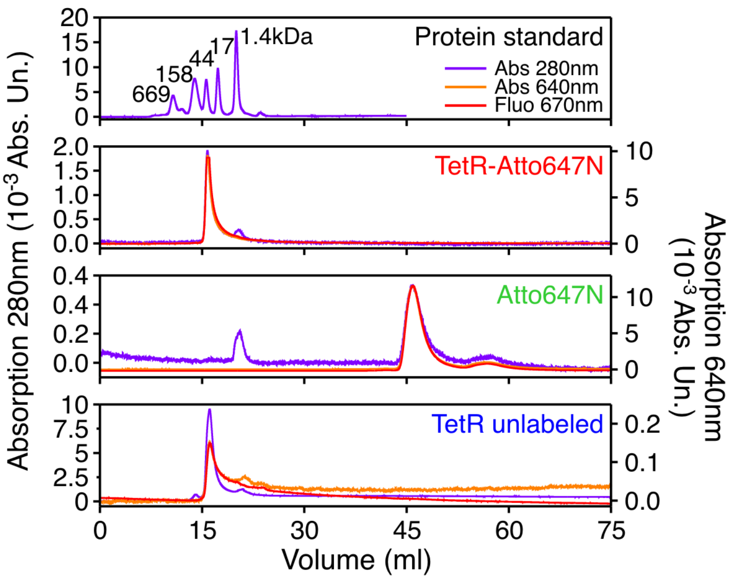

Supplementary Fig. 9 – HPLC analysis of TetR-Atto647N

The figure shows the measured absorption (at 280 nm purple lines and at 640 nm yellow lines) and the fluorescence emission (at 670 nm, red lines) in a HPLC experiment. The first time series corresponds to a recording of a protein standard composed by 5 proteins of different sizes (669 kDa, 158 kDa, 44 kDa, 17 kDa, and 1.4 kDa). The second panel reports the TetR-Atto647N labeled construct, the third panel the free dye (Atto647N), and the bottom panel the unlabeled TetR.

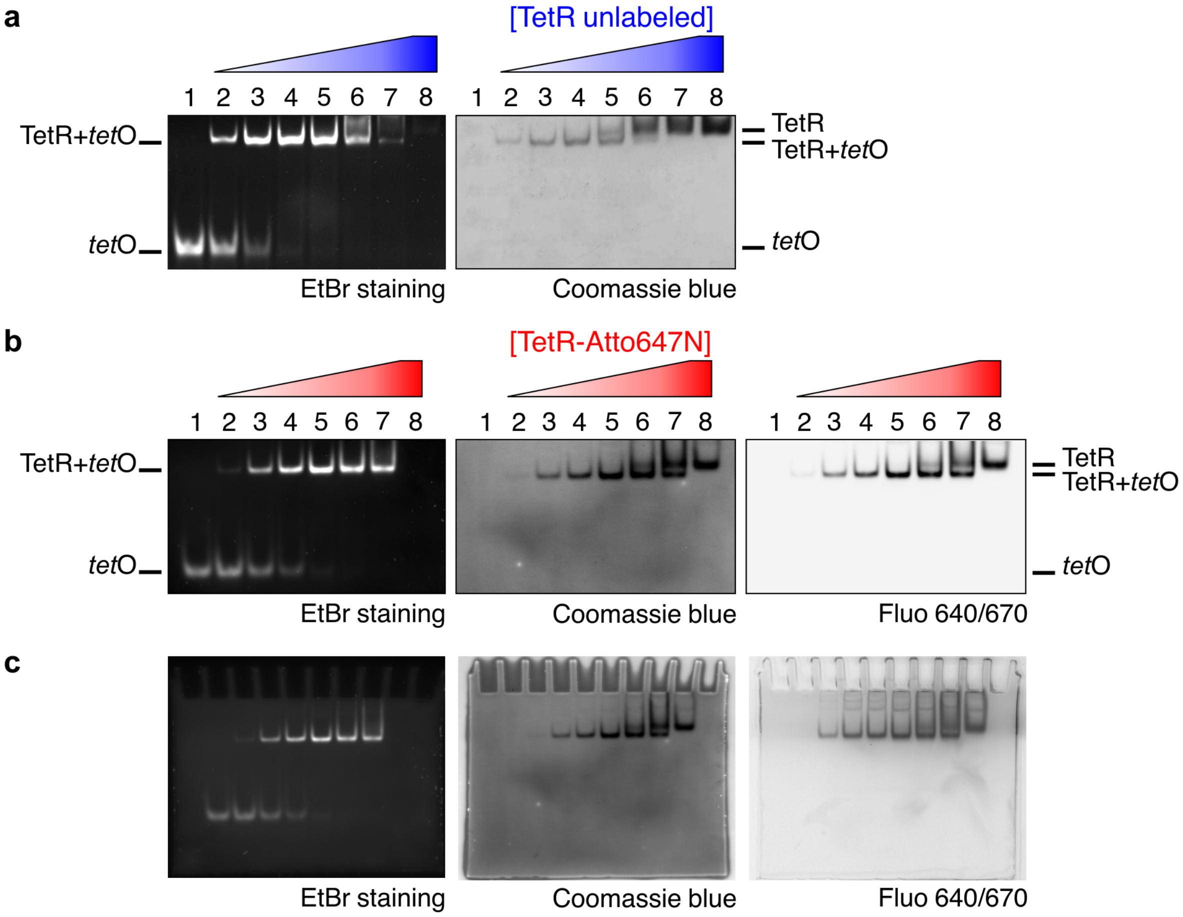

Supplementary Fig. 10 – Native gel-shift assay

The figure shows 10%-Polyacrylamide gels run in native conditions of the unlabeled TetR and of TetR-Atto647N. (**a**) Unlabeled TetR – we used 20 pmol of a 30bp-long dsDNA containing the canonical 19bp-long *tet*O site (lanes 1 to 7) and a gradient of unlabeled TetR (lane 2: 2.5 pmol; lane 3: 5 pmol; lane 4: 10 pmol; lane 5: 20 pmol; lane 6: 40 pmol; lane 7: 80 pmol; lane 8: 80 pmol). The first panel from left shows a picture of the gel using Ethidium Bromide staining to label the DNA, the second panel shows the unlabeled TetR protein obtained with Coomassie Blue staining of the gel. (**b**) TetR-Atto647N – we used 20 pmol of DNA (lanes 1 to 7) and a gradient of TetR-Atto647 (lane 2: 3.75 pmol; lane 3: 7.5 pmol; lane 4: 15 pmol; lane 5: 30 pmol; lane 6: 60 pmol; lane 7: 90 pmol; lane 8: 60 pmol). The left panel shows the staining of the gel with Ethidium Bromide, the central panel one with Coomassie Blue, and the right panel shows the fluorescence emission of the TetR-Atto647N proteins acquired with a fluorescence scan of the gel (Fluorescent Image Analyzer FLA-3000, FujiFilm) with 640 nm excitation and 670 nm emission. (**c**) Full-size images of the gel shown in Fig. 1b of the main text and in panel (b).

Next, in order to prove the protein activity *in vitro*, we ran electrophoretic gel-shift assay in native conditions. In particular, we incubated the unlabeled TetR and TetR-Atto647N proteins with dsDNA

oligos containing the 19 bp-long *tet*O sequence for 10 minutes at room temperature prior to loading and running the gel. As shown in Fig. 1b of the main text and in Supplementary Fig. 10, both the unlabeled protein and TetR-Atto647N bind to the *tet*O-containing oligos shifting their migration in the gel.

Finally, to probe that the labeling of TetR was not affecting the protein interaction kinetics with the DNA, we performed surface plasmon resonance experiments with a Biacore T200 (GE Healhcare, USA). In particular, we immobilized a 30 bp-long biotin-tagged dsDNA oligos containing the *tet*O to the surface of a streptavidin chip. Next, we sequentially flow the unlabeled TetR and the TetR-Atto647N at different concentrations (in the nano-molar range) monitoring the increase of the sensorgram signal *vs.* time (Supplementary Fig. 4a). The chip was regenerated with Dox (at 5 μg per ml) after each round of measure. *On* and *off* rates have been estimated with a single exponential fit of sensorgram signals and are plotted in Supplementary Fig. 4b. Notably, the association rate constant and the dissociation rate measured for the unlabeled TetR and for TetR-Atto647N are within the experimental error in good agreement.

TetR Micro-injection

TetR-Atto647N micro-injection experiments have been performed on an inverted microscope (IX71, Olympus, France) using a 60X phase objective (PlanApo 60X/1.40 Oil Phase 3, Olympus, France), a mercury lamp for fluorescence illumination and a, intensified CCD (CoolSNAP ES, Roper Scientific, Germany) for image collection, a micro-injector pump (Femtojet, Eppendorf, Germany) and a micro-manipulator (InjectMan NI2, Eppendorf, Germany) for semi-automatic injection. We used borosilicate glass capillaries (GC120TF-10, Harvard apparatus, USA) pulled with a Flaming/Brown micropipette puller (P-97, Sutter Instruments, USA) to have a conic apex with an aperture diameter ranging between 400 and 600 nm. Injection pressure ranged between 45 and 75 hPa, compensation pressure was set to 5 hPa and injection time to 0.2 s. Injection samples were diluted in filtered PBS and centrifugated at 16,300 *g* for 30 min at 4°C before experiments. The protein concentration in the injection needle used was ~1 μM for test purposes and about 50 nM for single-particle-tracking experiments.

Once injected at high concentrations (~1 μM in the injection tip), TetR-Atto647N recruited at a single locus that colocalized with the LacI locus (Fig. 1c of the main text, Supplementary Fig. 11 and Supplementary Movie 1) showing how the protein maintained its functionality also when injected in living cells. Moreover, to confirm the full functionality of the TetR-Atto647N construct, after injection we treated cell with Dox (at 2.5 μg per ml final concentration) and we observed a rapid dissociation of TetR-Atto647N from the target site (Fig. 1d of the main text, Supplementary Fig. 12 and Supplementary Movie 2) with release kinetics characterized by a decay constant of the order of 15 seconds, which well adjusts to that measured in the case of TetR-GFP (see Supplementary Note 3 and Supplementary Movie 2).

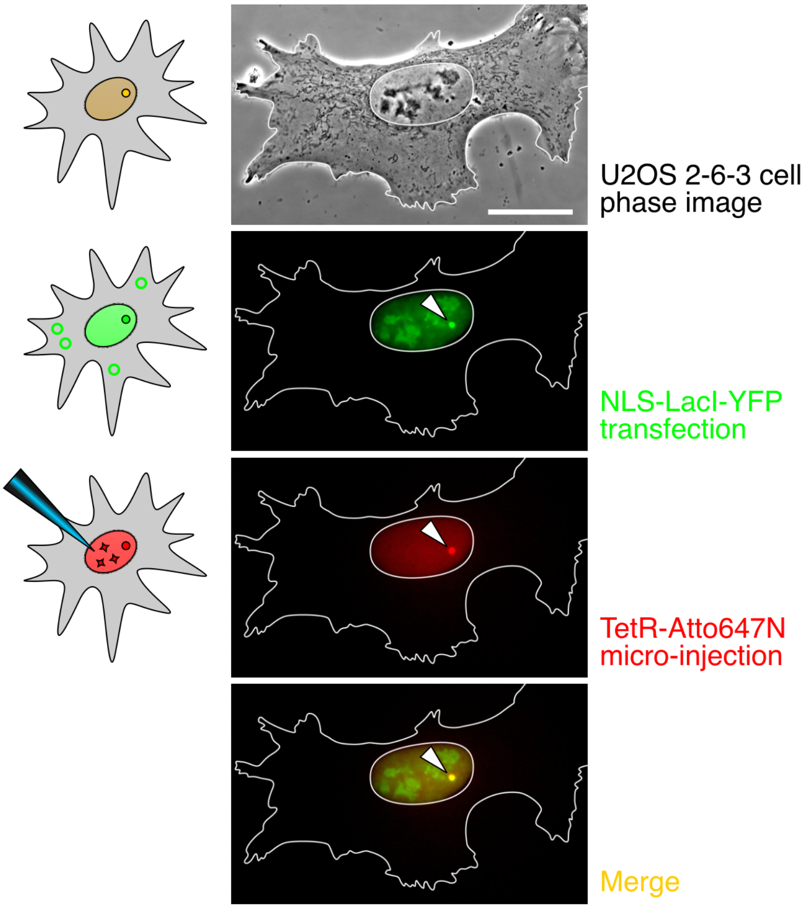

Supplementary Fig. 11 – TetR-Atto647N micro-injection and recruitment at the target locus
The figure shows the *in vivo* activity of the TetR-Atto647N construct. In order to probe our target search model assay for TetR-Atto647N, we selected a U2OS 2-6-3 cell (first panel) expressing the NLS-LacI-YFP construct, which accumulated at a single locus (white arrow, second panel), next we micro-injected the cell with 1 μM TetR-Atto647N in the injection needle (Supplementary Movie 1). As indicated by the white arrow (third panel), TetR-Atto647N also accumulated at a single locus that colocalizes with the LacI position (bottom panel), which shows how both proteins recognize and bind to their specific binding sites. Dark regions in the nucleus in the phase image (top) and bright green regions not pointed by the arrow in the YFP fluorescence channel (second panel) represent nucleoli, which occasionally present an accumulation of the LacI proteins. Scale bar 10 μm.

Finally, we tested the specificity of the TetR-Atto647N interaction at the target locus by pre-incubating the protein prior to inject with a 10 times molar excess of dsDNA oligos containing the *tet*O. In this case, once injected the TetR-Atto647N did not accumulated anymore at the target locus (Supplementary Fig. 13). Interestingly we observed the same effect when TetR-Atto647N was co-injected together with 1000-folds molar excess of unlabeled TetR proteins (Supplementary Fig. 14).

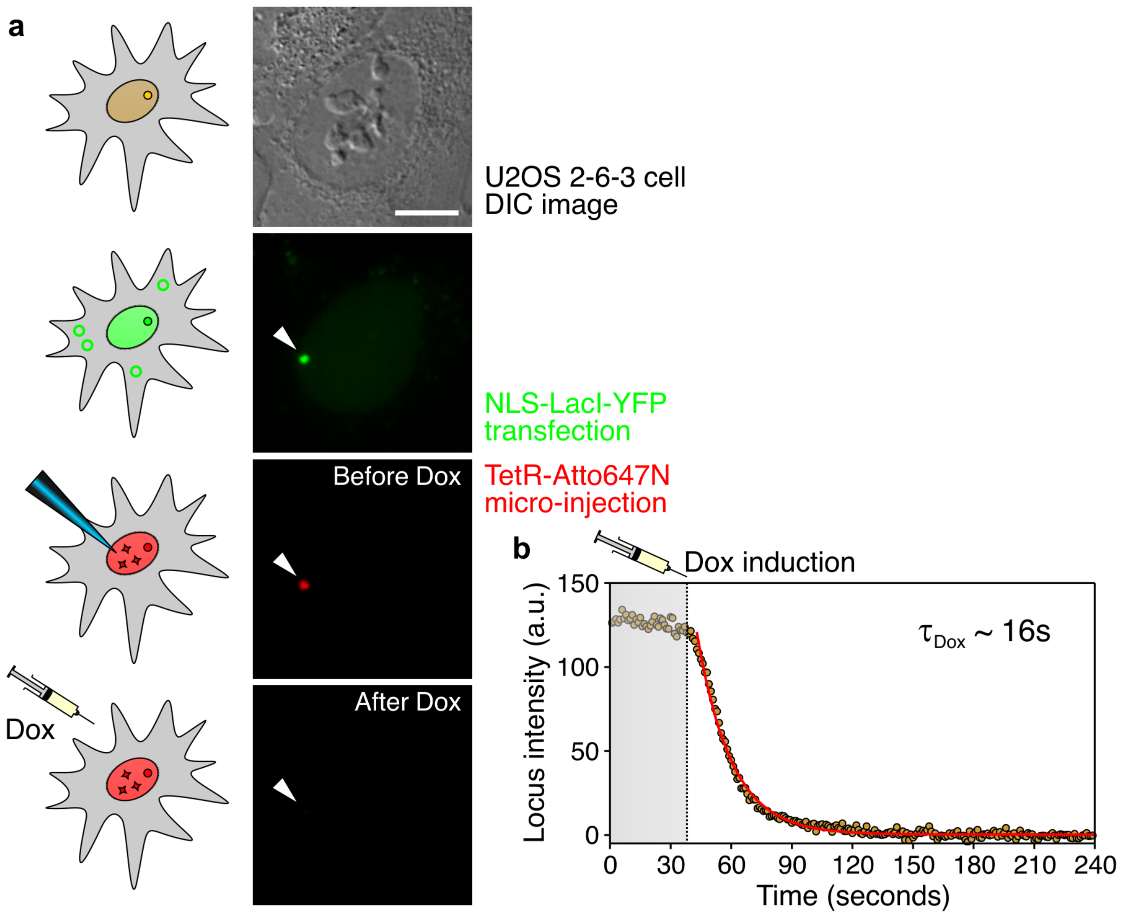

Supplementary Fig. 12 – Dox-induced TetR-Atto647N release from the binding site locus

(**a**) To probe TetR-Atto647N response to Dox treatment, we selected a U2OS 2-6-3 cell (top panel) expressing the NLS-LacI-YFP construct, which accumulated at a single locus (white arrow, second panel), and we micro-injected the cell with 1 μM TetR-Atto647N in the injection needle and it accumulated also at the LacI locus (third panel). Next, we rapidly added Dox to the cell medium and after few seconds of Dox treatment (final concentration 2.5 μg per ml) all TetR-Atto647N was released from the binding site (bottom panel). Scale bar 10 μm. (**b**) To estimate the Dox-induced TetR-Atto647N release kinetics, we monitored the intensity of the binding site locus versus time (brown symbols, see also Supplementary Movie 2) and we fitted it with a single exponential decay curve, obtaining a lifetime value of 16 ± 2 s for the example reported in the figure.

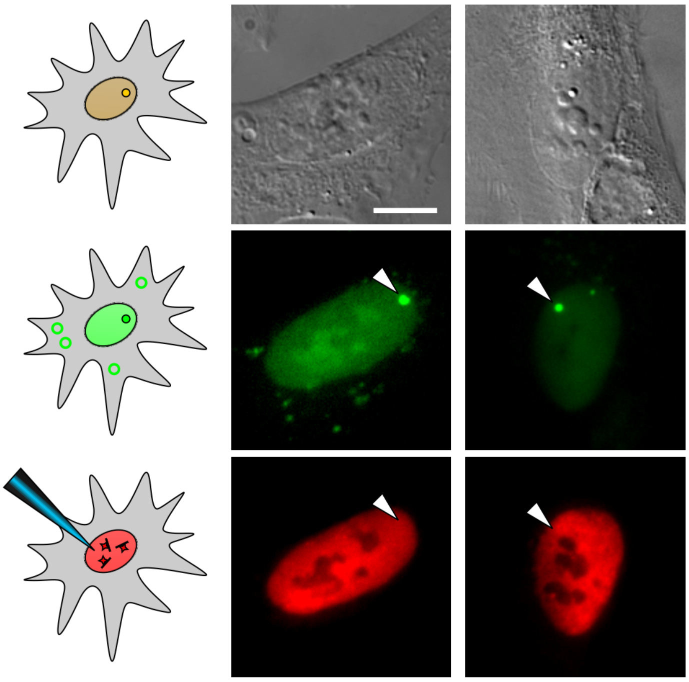

Supplementary Fig. 13 – Micro-injection of TetR-Atto647N pre-bound to *tet*O

The figure shows two U2OS 2-6-3 cells (top panels) transfected with NLS-LacI-YFP that accumulated at the binding site locus (white arrows, central panels) and micro-injected with 1 μM TetR-Atto647N pre-incubated for 20 minutes at 4°C (in the dark) with 10 times molar excess of *tet*O. As indicated by the white arrows (bottom panels), when the DNA binding domain of TetR-Atto647N is pre-occupied, the protein was no more capable of binding to DNA and did not accumulate at the binding sites locus in the cells. Dark regions in the nucleus (bottom panel) are nucleoli, which occasionally shown exclusion of the TetR protein. Scale bar 10 μm.

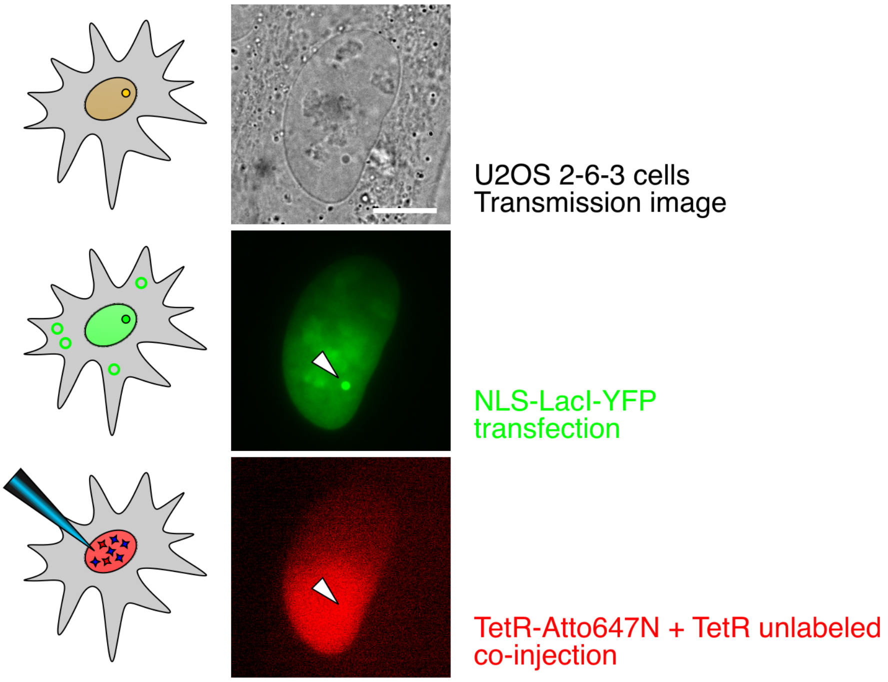

Supplementary Fig. 14 – Co-injection of TetR-Atto647N and unlabeled TetR

The figure shows a U2OS 2-6-3 cells (top panel) transfected with NLS-LacI-YFP that accumulated at the binding site locus (white arrows, middle panel) and micro-injected with ~200 nM TetR-Atto647N mixed with 1000 times molar excess of unlabeled TetR. As indicated by the white arrows (bottom panel), when a competitor protein (the unlabeled TetR) is present in large excess in the cell nucleus, TetR-Atto647N was no more recruited at the target locus. Scale bar 10 μm.

Supplementary Note 3

TetR-GFP assay

We tested our target search assay also with TetR-GFP. Notably, when TetR-GFP was endogenously expressed in U2OS 2-6-3 cells, we observed an accumulation of TetR-GFP at a single bright locus, which co-localized with the LacI signal (Supplementary Fig. 15a, top images). Next, we investigated the TetR-GFP response to Dox. Again, when rapidly adding Dox (final concentration 2.5 μg per ml) to the cells medium, we observed a rapid decrease of the TetR-GFP signal (Supplementary Fig. 15b and Supplementary Movie 2) and when we quantified the kinetics of the Dox-induced TetR dissociation form the binding site (Supplementary Fig. 15c), we found close agreement with the data obtained for TetR-Atto647N (see Supplementary Note 2).

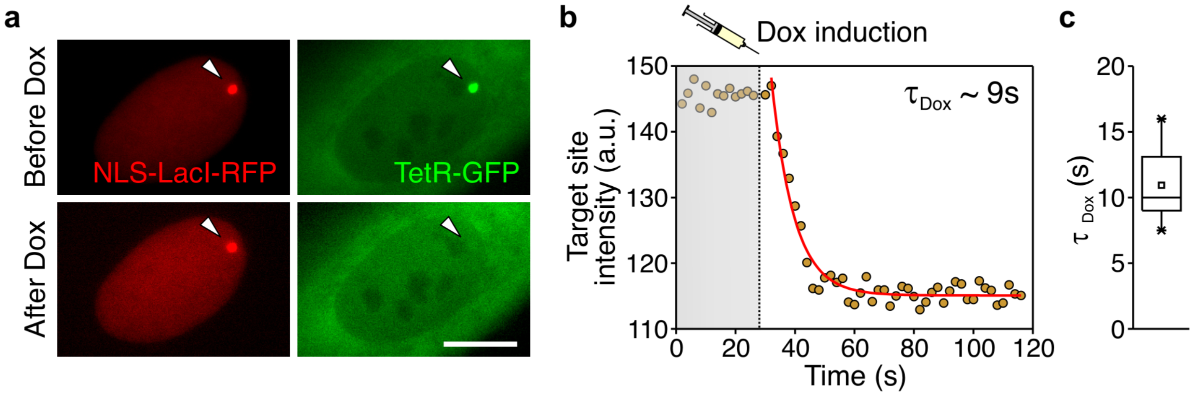

Supplementary Fig. 15 –TetR-GFP assay

(**a**) Fluorescence images of NLS-LacI-RFP and TetR-GFP before (top) and after (bottom) Dox treatment (Dox final concentration 2.5 μg per ml). The white arrows point to the binding site locus, which is completely depleted within few seconds of Dox treatment. Scale bar 10 μm. (**b**) TetR-GFP release kinetics. When rapidly adding Dox (final concentration 2.5 μg per ml) to the cell medium, after a lag time of the order of 3 to 5 seconds, the integrated intensity at the binding site locus rapidly decreases with time. The red curve represents the best fit to the data of a mono exponential function with decay constant of the order of 9 seconds. (**c**) Box plot of the Dox-induced decay time constants for different cells (N = 8 cells). The decay time constant measured is 11 ± 3 s (mean ± s.d.).

Supplementary Note 4

Single-Particle-Tracking experiments

Experimental conditions

Single-Particle-Tracking (SPT) experiments on TetR-Atto647N proteins have been conducted on an inverted microscope (IX71, Olympus, France) in wide-field configuration by focusing an illumination laser (Cube 640-100C, Coherent, USA) in the back focal plane of a 150X objective lens (UAPON 150XOTIRF, Olympus, France). A dichroic filter (FF635-Di01-25×36, Semrock, USA) and a long pass emission filter (BLP01-635R-25, Semrock, USA) have been used to couple the laser to the microscope and direct fluorescence light to a 128 × 128 pixels back-illuminated Electron-Multiplied Charge-Couple-Device (EM-CCD) (iXonEM DV860DCS-BV, Andor, Ireland), run in frame transfer mode (exposure time 5 ms) and controlling laser illumination via an acousto-optic tunable filter (AOTFnC-400-650-TN, A&A Optoelectronic, France). Laser density of energy on the sample was on the order of 0.1 kW per cm^2, the field of view 20.5 μm × 20.5 μm, and the pixel size 160 nm.

In particular, we injected TetR-Atto647N proteins at low concentrations (~50 nM in the pipette), in a regime where between 50 and 500 fluorescent molecules could be detected in the cell nucleus and we recorded (typically 50 s-long) movies with 5 ms exposure time and a frame rate of 197 frames per second. At the beginning of the recordings, we usually observed bright fluorescent spots at the target locus with a signal decreasing in a step-wise manner, likely corresponding to the sequential photo-bleaching of stably bound TetR-Atto647Ns (Supplementary Fig. 16a).

Trajectories analysis

In order to analyze TetR-Atto647N mobility, we generated single-molecule trajectories from our movies (Supplementary Movie 3). In particular, we used the MatLAB (MatLab 7.0, Mathworks Inc., USA) script SLIMfast, which is based on the Multiple-Target-Tracing (MTT) algorithm (16), to first detect and then reconnect the particle positions in all frames. The localization and tracking parameters used are reported in Supplementary Table 4. The localization precision achieved in the experiments was on the order of 25 nm and the longest traces recorded for TetR-Atto647N ranged up to few seconds (~1000 frames). The bleaching properties of TetR-Atto647N in the nucleus of living cell have been characterized by monitoring the integrated intensity of cell nuclei *vs.* time under the same illumination conditions used for single-molecule tracking and are shown in Supplementary Fig. 16b. The mean bleaching decay time constant measured was 2.9 ± 0.7 s or 580 ± 140 frames (mean ± s.d., N = 5 cells).

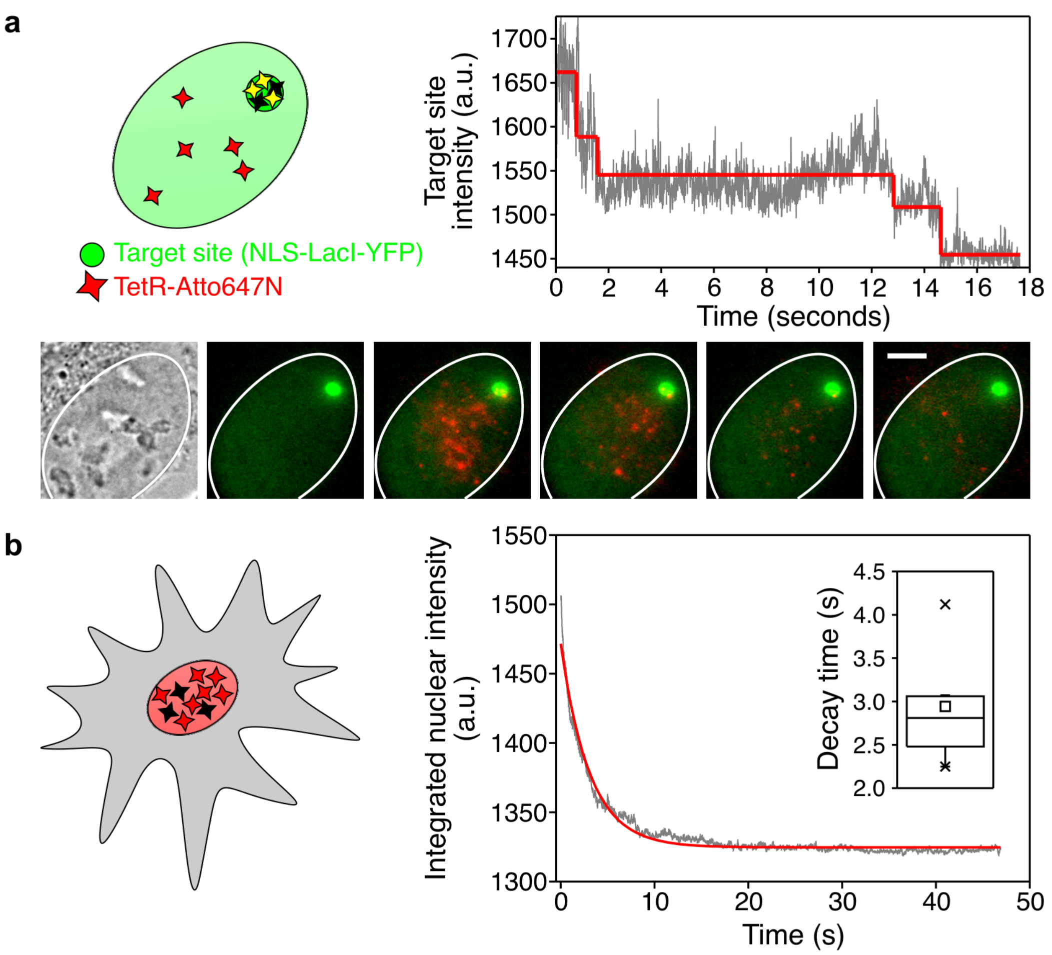

Fig. 16 – Injected TetR-Atto647N bleaching characteristics

(**a**) Sequential photobleaching of TetR-Atto647N proteins injected and bound at the target locus. Top left: Schematics drawing of the experiment: the cell nucleus is shown in green, the target site, visualized thanks to the accumulation of NLS-LacI-YFP proteins, is represented in dark green, and the individual TetR-Atto647N molecules as red stars. TetR-Atto647N proteins bound at the target locus are shown in yellow. Top right: Time-series (grey curve) corresponding to the intensity of the target locus *vs.* time for the cell shown in Fig. 2a of the main text. The red curve is a guide-to-eye curve evidencing the sequential individual photo-bleaching steps. Bottom: From left to right, transmission image of the nucleus of a cell, NLS-LacI-YFP signal (green) indicating the position of the target locus, subsequent snapshots of TetR-Atto647N (red spots overlaid to the green image) showing 4, 2, and 1 protein (yellow spots) bound at the target locus. Scale bar 5 μm. (**b**) Global bleaching properties of the injected TetR-Atto647N proteins. The graph shows the integrated nuclear intensity *vs.* time (grey curve) and its corresponding fit with a single exponential function (red curve) with time decay constant of 3.06 ± 0.01 s. The inset shows a box plot of the bleaching decay time constants measured whose mean value is $\tau_{Bleaching}$ = 2.9 ± 0.7 s (mean ± s.d., N = 5 cells).

Localization error	10^{-6}
Deflation loops	2
Max D ($\mu m^2 \cdot s^{-1}$)	10
Blinking (frames)	5
Max # competitors	3

Supplementary Table 4 – Localization and tracking parameters in SPT analysis
The table reports the values of the parameters used to localize and generate individual trajectories of TetR-Atto647N proteins from SPT experiments.

In order to analyze the diffusion properties of TetR-Atto647N, we calculated the Mean Square Displacement (MSD) (17) for each trajectory longer than 8 frames (see Fig. 2c of the main text and Supplementary Movies 3 and 4) using a custom written LabVIEW (LabVIEW 7.1, National Instruments, USA) routine (18). Subsequently, the instantaneous diffusion coefficients D_{Inst} have been obtained by unconstrained linear fitting of the MSD curves between time lag 2 and 5 (17) and D_{Inst} calculated as $MSD_{SLOPE}/4$. The resulting distributions of the logarithm of the instantaneous diffusion coefficients (Log D_{Inst}) obtained for the different conditions (reported in Fig. 2b of the main text) have been fitted with a triple Gaussian function (yellow curves in Fig. 2b of the main text). We used log-normal fits, not for theoretical reasons but in order to easily distinguish subpopulations. To our opinion, the results of the experiments shown in Fig. 2 of the main text support this approach and in particular enable the identification of a slow subpopulation as DNA-bound proteins and the estimate of the fraction of the TetR non-specifically interacting with DNA. The resulting values of the diffusion coefficients and relative abundance of the different populations for all the 4 conditions tested (basal condition, with Dox, with *tet*O, and with competitor unlabeled TetR proteins) are reported in Supplementary Table 5. Supplementary Fig. 17 shows the distribution of the trajectory length for the 4 different datasets considered in the analysis; mean trajectory lengths and datasets statistics are reported in Supplementary Table 5.

Of note, the lower diffusion coefficient ($D_1 \sim 3\ \mu m^2 \cdot s^{-1}$) of the fast population reported for the *tet*O co-injection experiments can be explained by different factors. On one side, the complex TetR-*tet*O-containing 30mer oligos is physically larger than the free protein and this will reduce the diffusion coefficient in the nucleoplasm (according to the Stokes law for a globular protein, D scales as $1/a$ where a is the radius of the protein). On the other side, we used different experimental conditions for those experiments. Specifically, the frame rate used for image acquisition in *tet*O co-injection experiments was 100 Hz, in comparison with all the other experiments conducted at a frame rate of 197 Hz. The slower acquisition rate indeed limits the maximum diffusion coefficient measurable.

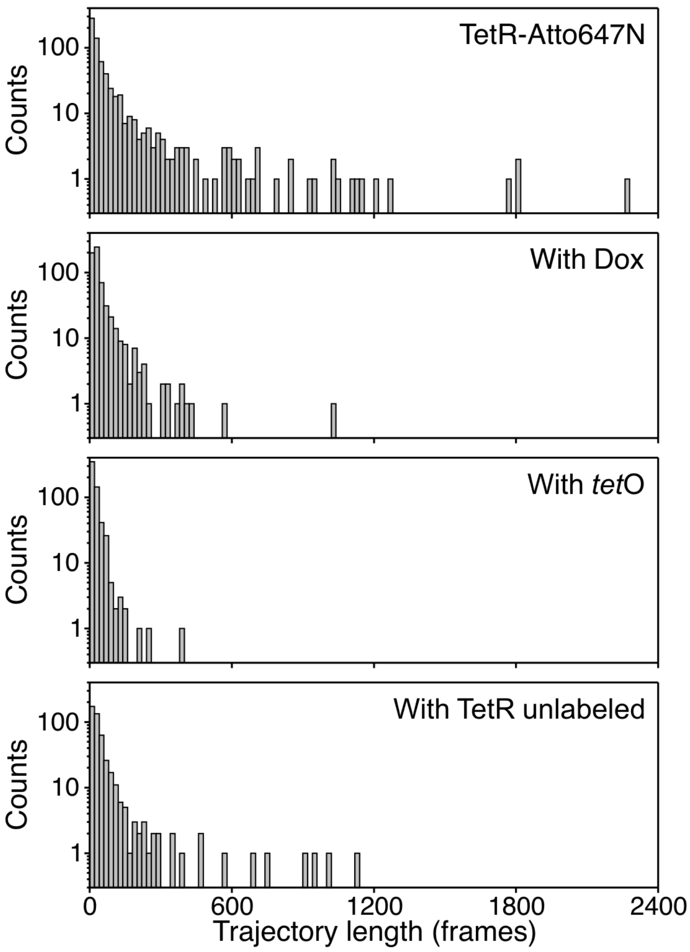

Supplementary Fig. 17 – Trajectory length distribution in the different experimental conditions
The figure shows the distribution of the trajectory length (expressed in number of frames) in each of the different experimental conditions tested. From top to bottom: in the absence of Dox - basal conditions; in the presence of Dox at 2.5 μg per ml final concentration; upon *tet*O (10X molar excess) co-injection; and in the case of 1000 folds excess of competitor unlabeled TetR proteins.

SPT experiments on TetR labeled with different organic dyes

To rule out the possible contribution of the organic dye used to label TetR, we tested three different dyes, all emitting in the far red but with different electrical charge and chemical structure (see Supplementary Fig. 8c). In particular, together with Atto647N (positively charged), we used Atto655 (neutral), and Cy5 (negatively charged). Despite the fact that for TetR-Atto655 and TetR-Cy5 trajectories were substantially shorter than in the case of TetR-Atto647N (Supplementary Fig. 18 and Supplementary Table 6), also in those cases we could recapitulate the same diffusion properties for TetR with a fast free-diffusing population, an intermediate one, and a third population of greatly reduced mobility and Dox-sensitive (Supplementary Fig. 19 and Supplementary Table 6).

	TetR-Atto647N			
	**Basal conditions**	**+ Dox**	**+ *tet*O**	**+ Unlabeled TetR**
D_1 ($\mu m^2 \cdot s^{-1}$)	7.8	5.9	2.6	5.8
f_1	0.33	0.23	0.73	0.33
D_2 ($\mu m^2 \cdot s^{-1}$)	1.1	1.7	0.7	0.6
f_2	0.43	0.76	0.23	0.48
D_3 ($\mu m^2 \cdot s^{-1}$)	0.11	0.09	0.08	0.09
f_3	0.24	0.01	0.04	0.19
Number of trajectories	682	623	572	460
Mean trajectory length (frames)	89	47	25	58
Number of cells	10	8	4	3

Supplementary Table 5 – SPT experiments of TetR-Atto647N in different conditions

The table shows the central values of the instantaneous diffusion coefficients (D_{Inst}) and the fraction f of the different populations (1: fast freely diffusing proteins; 2*:* intermediate population; 3: quasi-immobile proteins) observed in our SPT experiments with TetR-Atto647N in the absence of Dox (basal condition) and in the presence of Dox in the cell medium (2.5 µg per ml final concentration), and if TetR was pre-incubated with the *tet*O oligos (10X molar excess) or co-injected with 1000-folds excess of unlabeled proteins. The table reports also the statistics regarding the number of trajectories used in the analysis, the mean trajectory length, and number of cells observed in the different experimental conditions.

Given the superior quality of Atto647N in terms photo-stability (length of the trajectories recorded) and brightness (Supplementary Fig. 20), we decided to concentrate our experiments on the TetR-Atto647N construct. Of note, the poor optical properties of Atto655 (Supplementary Fig. 20) are such that the fast moving particles often generated blurred images, not easily detectable over the background, and thus difficult to localize and track. This results in the tendency of under detecting the fast population and under estimating its diffusion coefficient when tracking TetR-Atto655.

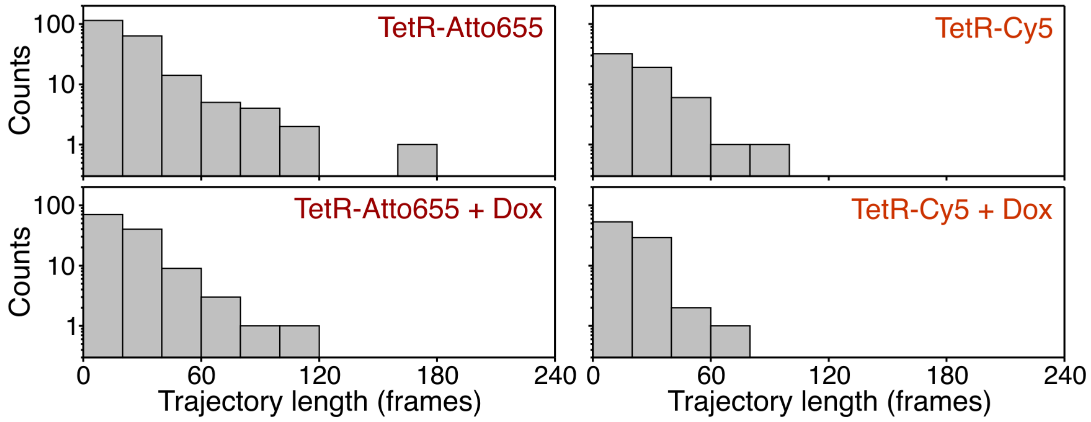

Supplementary Fig. 18 – Trajectory length distribution for TetR-Atto655 and TetR-Cy5

The figure shows the distribution of the trajectory length (expressed in number of frames) obtained for TetR-Atto655 (left) TetR-Cy5 (right) in the absence of Dox (top) and in the presence of Dox at 2.5 μg per ml final concentration (bottom).

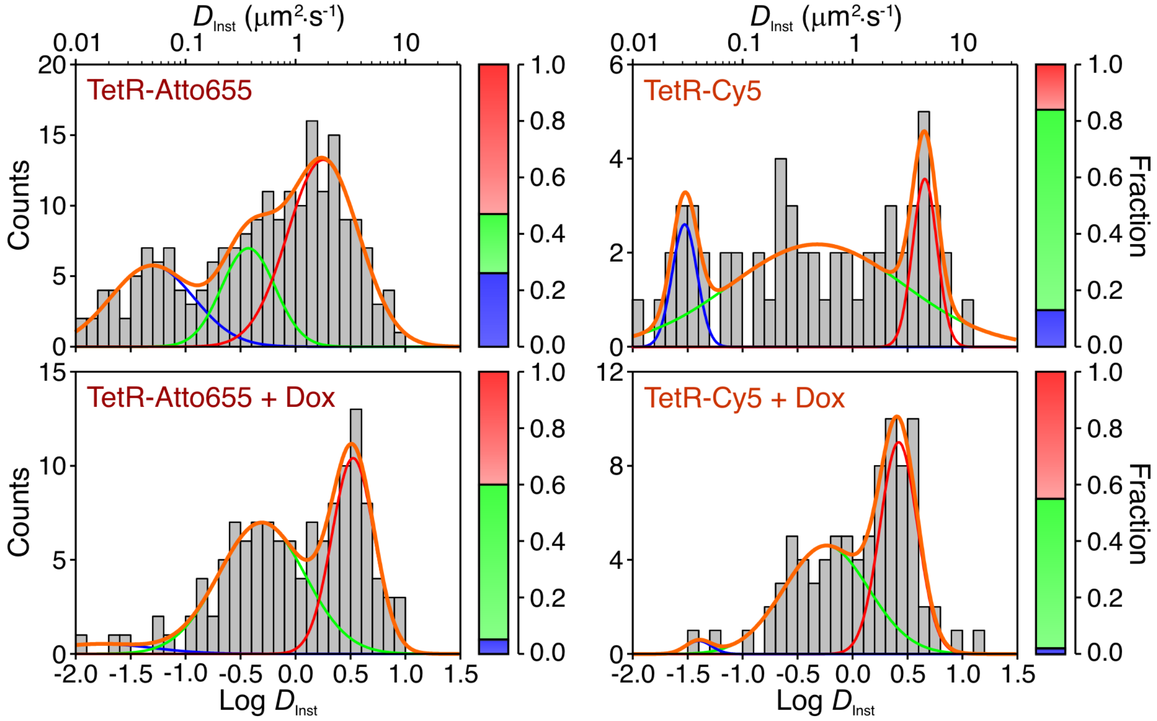

Supplementary Fig. 19 – SPT experiments of TetR labeled with different organic dyes

The figure shows the distribution of the instantaneous diffusion coefficients D_{Inst} obtained for TetR-Atto655 (left panels) and TetR-Cy5 (right panels) in the absence of Dox (top panels) and in the presence of Dox at 2.5 μg per ml (bottom panels). As for the TetR-Atto647N (see Fig. 2b of the main text), trajectories could be grouped into three different mobility categories in the absence of Dox, while in the presence of Dox, trajectories of greatly reduced mobility were almost completely abolished. The fit results are summarized in Supplementary Table 6.

	TetR-Atto655		TetR-Cy5	
	– Dox	+Dox	– Dox	+ Dox
D_1 (μm²·s⁻¹)	1.8	3.3	4.5	2.6
f_1	0.53	0.4	0.16	0.45
D_2 (μm²·s⁻¹)	0.4	0.5	0.5	0.6
f_2	0.21	0.55	0.71	0.53
D_3 (μm²·s⁻¹)	0.05	0.02	0.03	0.04
f_3	0.26	0.05	0.13	0.02
Number of trajectories	203	124	59	85
Mean trajectory length (frames)	24	23	24	19
Number of cells	5	4	3	3

Supplementary Table 6 – SPT experiments of TetR labeled with different organic dyes

The table shows the central values of the instantaneous diffusion coefficients (D_{Inst}) and the fraction *f* of the different populations (1: fast freely diffusing proteins; 2: intermediate population; 3: quasi-immobile proteins) observed in our SPT experiments with TetR labeled with a neutral (Atto655) and a negatively charged (Cy5) organics dye, in the presence (2.5 μg per ml final concentration) and in the absence of Dox in the cell medium. The table reports also the statistics regarding the number of trajectories used in the analysis, the mean trajectory length, and number of cells observed in the different experimental conditions.

Confinement analysis

Looking more into the details of individual trajectories, we observed that a subset of the intermediate population (green population in Fig. 2b,c of the main text) clearly showed the signature of confined diffusion (Supplementary Fig. 2a). We used equation here below for restricted diffusion (19) to fit individual MSD curves and extract the radius of confinement R_{Conf}:

$$y(t) = R_{\mathrm{Conf}}^2\left(1 - A_1 \cdot \exp\left(-4A_2Dt/R_{\mathrm{Conf}}^2\right)\right) , \quad (1)$$

with *D* the diffusion coefficient and considering the parameters A_1 = 0.99 and A_2 = 0.85, as for a circular domain (20,21).

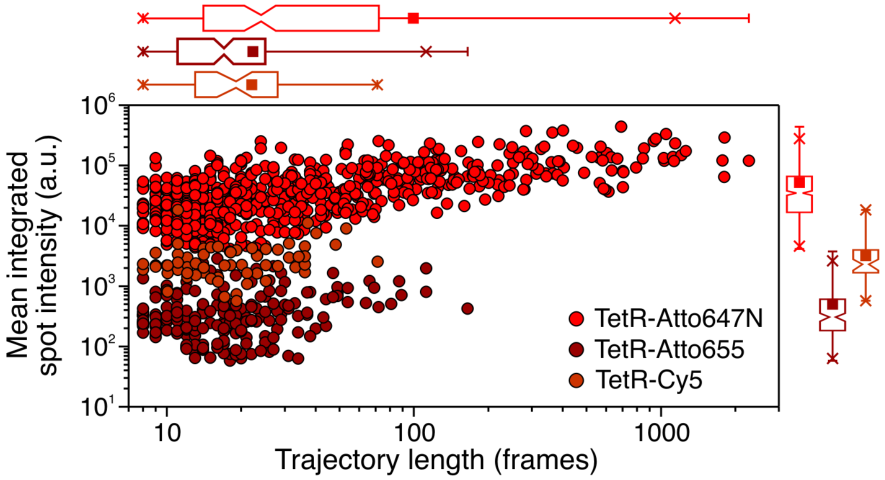

Supplementary Fig. 20 – Global performances of the different TetR constructs

The figure shows the correlation between the brightness (expressed as the mean of the integrated intensity of the spot localizations along an individual trajectory) and the length (in frames) of the trajectory for the different dyes used. Red circles represent TetR-Atto647N trajectories, brown circles TetR-Atto655, and light brown circles TetR-Cy5. Notched box plots on the top and right show the mean (squares), the 1/99 percentile (crosses), and the full distribution (ticks) for the different dyes.

The distribution of the R_{Conf} is reported in Supplementary Fig. 2c and is peaked at 0.77 ± 0.02 μm (mean ± s.d.). Next, we investigated if the presence of confinement of the intermediate population was still present when inhibiting TetR binding to DNA using Dox.

We found that also in this case a subset of the proteins showed a confined behavior (Supplementary Fig. 2d) with R_{Conf} = 0.76 ± 0.02 μm (mean ± s.d.).

Altogether our results about the confined behavior of proteins sustain the view that these transient confinement regions are somehow related to the nuclear environment regardless of the interaction of TetR with the DNA via its DNA-binding-domain.

Finally, contrarily to transient local confinement, over the time scale of our MSD analysis (a few hundreds of milliseconds), we did not observe any signature of overall confinement inside the nuclear envelope of the cells.

Supplementary Note 5

TetR/Rev-TetR mobility analysis by sptPALM

Photo-activation single-particle-tracking experiments (sptPALM) (22) were performed and analyzed as previously described (23). Briefly, we used an inverted microscope (Ti Eclipse, Nikon, France) equipped with a perfect focus system (Nikon, France), an *Intensilight* liquid guide (Nikon, France) in combination with a filter wheel (Lambda 10-3, Sutter Instruments, USA) for regular fluorescence imaging, and a TIRF arm (Nikon, France) to achieve wide field tilted laser illumination (24). For single-molecule imaging we employed a 561 nm imaging laser (Genesis MX 561-2000 MTM, Coherent, USA) and a 405 nm activation laser (Cube 405-100C, Coherent, USA) focused in the back focal plane of a 100X N.A. 1.49 oil immersion objective (CFI Apo TIRF 100X, Nikon, France) and a 512 × 512 EM-CCD (Ixon3 DU897, Andor, Ireland). All instrumentation was controlled via NIS Elements (Nikon, France).

sptPALM experiments have been conducted on stable cell lines, derived from U2OS 2-6-3 cells, and constitutively expressing TetR-Dendra2 or RevTetR-Dendra2 at low level as selected by FACS sorting (ARIA III, BD Bioscience, USA). Cells were first imaged in GFP in order to select a region of interest (ROI) in the nucleus, away from the binding sites locus. Time-lapse images of the ROI were then acquired with 10 ms exposure time under continuous illumination (561 nm laser, ~1 kW per cm^2) and pulsed activation (405 nm laser, typically one 10 ms-long activation pulse at 0.01 kW per cm^2 per second even though the frequency and the power on the activation laser were tuned in order to achieve a constant density of photo-converted proteins (typically less that one molecule per frame inside the ROI, see Supplementary Movie 5). The low brightness of Dendra2 in combination with the fast motion of proteins most often gave raise to “motion blurs” rather than diffraction limited spots in the image, preventing the use of 2D Gaussian fitting to detect and localize the proteins. We therefore used a graph theory-based algorithm to group pixel belonging to a “motion blur”, whose position was inferred by centroid calculation after background subtraction (23). After localization, the positions of the particles detected in consecutive frames were linked together in order to generate trajectories. While, on one side, the limited photo-stability of Dendra2 in combination with the fast motion of proteins resulted in very short trajectories (a few frames in our experimental conditions), on the other side, controlled photo-conversion of Dendra2 permitted to record thousands of trajectories in the same cell. Trajectories have been analyzed computing the distance traveled between consecutives frames (translocation) and generating translocations histograms (grey columns in Supplementary Figs. 21 and 22). Next, the distributions have been corrected (purple bars in Supplementary Figs. 21 and 22) for the over-representation of slowly diffusing proteins due to the rapid out-of-focus motion of fast diffusing molecules using simulations fed with the relevant experimental parameters (23).

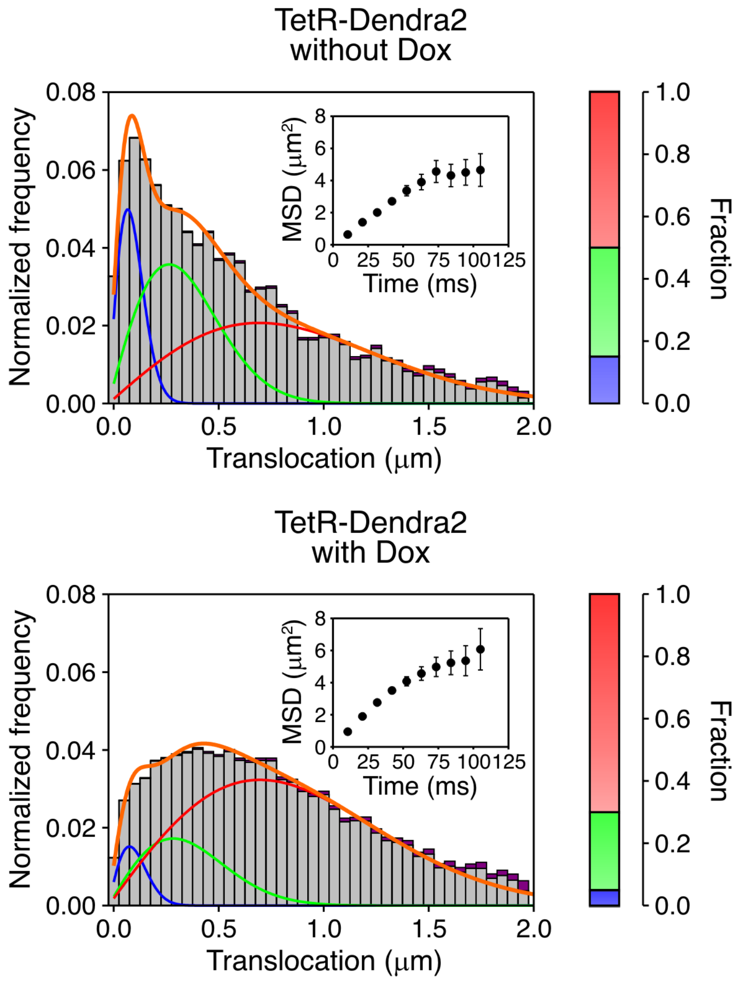

Supplementary Fig. 21 – TetR mobility by sptPALM

The figure shows the translocations distribution obtained at 1 time lag (10 ms) for TetR-Dendra2. The behavior of TetR-Dendra2 is very similar to that of TetR-Atto647N. Basically, it showed two mobile populations and a third one of greatly reduced mobility in the absence of Dox (top panel), while in the presence of Dox (2.5 μg per ml final concentration) it basically showed only the two mobile populations (bottom panel). Colored lines are the translocation distributions at 10 ms calculated assuming the diffusion coefficient D_i obtained by CPD fitting and normalized according to the relative abundance (color bars on the right) of the fast (red), intermediate (green) and quasi-immobile (blue) populations. Insets show the ensemble average MSD curves. Data obtained with the three components fit are summarized in Supplementary Table 7.

Once corrected, translocation histograms are used to calculate the Cumulative Probability Distribution (CPD or normalized frequency of occurrences) of the squared translocations at different ΔT, which are then fitted with a 3 components model using the formula reported here below (25):

$$\mathrm{CPD}(r^2, t) = 1 - \left[f_1 \cdot \exp\left(\frac{r^2}{r_1^2}\right) + f_2 \cdot \exp\left(\frac{r^2}{r_2^2}\right) + (1 - f_1 - f_2) \cdot \exp\left(\frac{r^2}{r_3^2}\right)\right]. \quad (2)$$

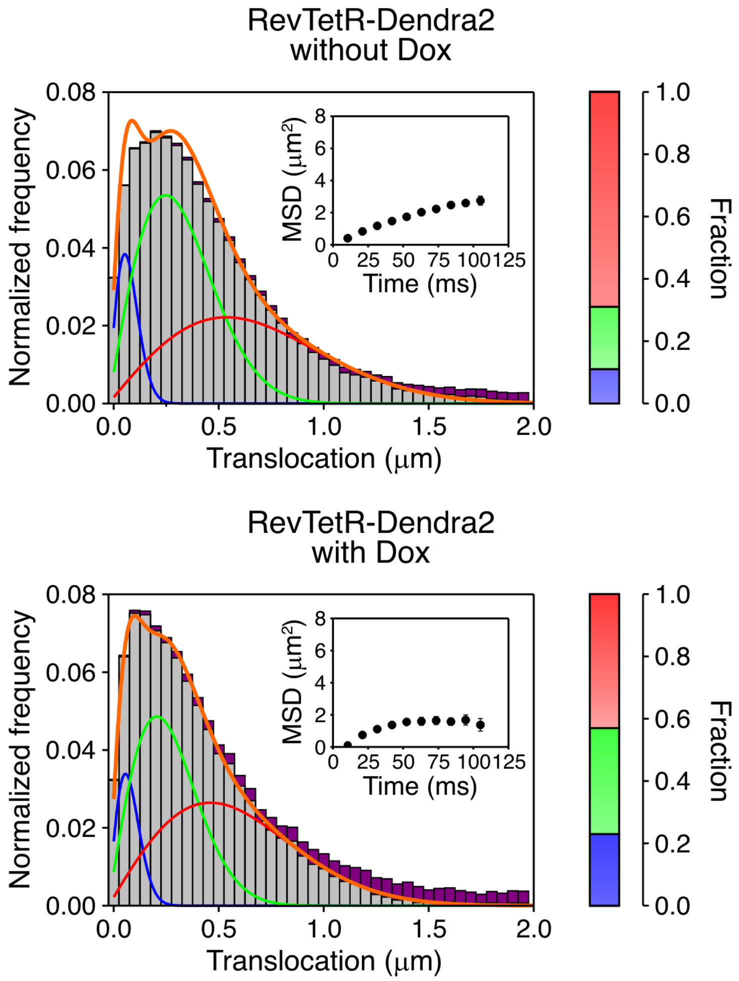

Supplementary Fig. 22 – RevTetR mobility by sptPALM

The figure shows the behavior of RevTetR-Dendra2 in sptPALM experiments. Notably, RevTetR-Dendra2 presents a behavior similar to that of TetR with inverse response to Dox treatment: in the absence of Dox (top panel), RevTetR-Dendra2 essentially showed two mobile populations (even though in this case the population of greatly reduced mobility was not completely abolished by Dox treatment) while in the presence of Dox (2.5 μg per ml final concentration) it showed also a pronounced third population of greatly reduced mobility (bottom panel). Colored lines are the translocation distributions at 10 ms calculated assuming the diffusion coefficient D_i obtained by CPD fitting and normalized according to the relative abundance (color bars on the right) of the fast (red), intermediate (green) and quasi-immobile (blue) populations. Insets show the ensemble average MSD curves. Data obtained with the three components fit are summarized in Supplementary Table 7.

The fractions f_i are the relative abundance of each population (right color bars in Supplementary Figs. 21 and 22) and the parameters r_i are related to the diffusivity of each population via the relation (25):

$$r_i^2(t) = 4D_i \cdot t. \tag{3}$$

The results of our analysis of TetR-Dendra2 and RevTetR-Dendra2 mobility in the presence and in the absence of Dox are reported in Supplementary Table 7. Overall, thanks to a rigorous analysis procedure, sptPALM experiments lead to an unbiased ensemble measurement of protein mobility (23). Notably, TetR-Dendra2 dynamics closely match what we obtained for TetR-Atto647N in terms of diffusion coefficients and partitioning between the three different populations.

	**TetR-Dendra2**		**Rev-TetR-Dendra2**	
	**Without Dox**	**With Dox**	**Without Dox***	**With Dox***
D_1 ($\mu m^2 \cdot s^{-1}$)	16	20	9.3	12.3
f_1	0.5	0.7	0.69	0.43
D_2 ($\mu m^2 \cdot s^{-1}$)	1.4	3.3	2.6	1.2
f_2	0.35	0.25	0.2	0.34
D_3 ($\mu m^2 \cdot s^{-1}$)	0.1	0.1	0.1	0.1
f_3	0.15	0.05	0.11	0.23
Number of trajectories	23341	18424	25821	15807
Number of cells	8	9	13	9

Supplementary Table 7 – sptPALM mobility experiments

The table shows the values of the diffusion coefficients *D* and the fraction *f* of the different populations (1: fast freely diffusing proteins; 2: intermediate population; 3: quasi-immobile proteins) observed in our sptPALM experiments with TetR-Dendra2 and RevTetR-Dendra2 in the presence (2.5 μg per ml final concentration) and in the absence of Dox in the cell medium.

* Data refers to the sixth time lag (6th ΔT).

Supplementary Note 6

TetR-GFP FRAP experiments

We also probed the mobility and test the possible non-specific interactions with DNA of TetR fused to GFP by means of Fluorescence Recovery After Photobleaching (FRAP) experiments in the nucleoplasm, away from the target locus. TetR-GFP FRAP experiments in U2OS 2-6-3 cells were conducted on a Nikon Ti2000 microscope, equipped with perfect focus system (PFS, Nikon, France), a Xenon light source (DG4, Sutter Instruments, USA) for epi-fluorescence imaging, two filter wheels (Lambda 10-3, Sutter Instrument, USA) to select excitation and emission wavelengths, and a 512 × 512 EM-CCD (QUANTEM, Roper Scientific, Germany) to grab images. Local photobleaching of TetR-GFP was achieved by focusing a 488 nm Argon laser through a 60X oil immersion objective (Nikon, France). We used an AOTF (A&A Optoelectronics, France) to obtain temporal control of the laser and a scanning module (FRAP head) based on galvanometric mirrors (iLAS, Roper Scientific, Germany) to spatially locate the bleaching point inside the cells. The microscope and the FRAP head were controlled via MetaMorph (Universal Imaging, USA).

Photobleaching was generated in 2 μm-radius region into the cell using 1,5 mW of laser power and 50 ms of total time of irradiation (Supplementary Fig. 23a and Supplementary Movie 6). The TetR-GFP mobility, *i.e.* its recovery, was monitored via time-lapse imaging upon blue light illumination every 100 ms and during 10 to 20 seconds after photobleaching. In order to quantify TetR-GFP mobility and obtain the FRAP curves, we determined the temporal evolution of the radial intensity profile $I_{\mathrm{r}}(t)$ in the FRAP region, first we subtracted the background in the images and corrected for GFP bleaching during the recording using a custom written MatLab (MatLab 7.0, Mathworks Inc., USA) routine (26). Once, normalized by the intensity profile of the last image before photo-bleaching, $I_{\mathrm{r}}(t)$ was fitted with a constant function with a Gaussian flank (26):

$$I_{\mathrm{r}}(t) = \begin{cases} A & , \ for\, r \leq r_{\mathrm{c}} \\ 1-(1-A)\exp\left(-\frac{(r-r_{\mathrm{c}})^2}{2\sigma^2}\right), & for\, r > r_{\mathrm{c}} \end{cases} , \quad (4)$$

where r_{c} is the radius of the bleaching region, σ *is* the width and *A* the amplitude of the Gaussian fit of the bleach profile. Individual intensity profiles $I_{\mathrm{r}}(t)$ were recorded in several cells, normalized between 0 and 1, and then averaged together to obtain the average FRAP curves shown in Supplementary Fig. 23b.

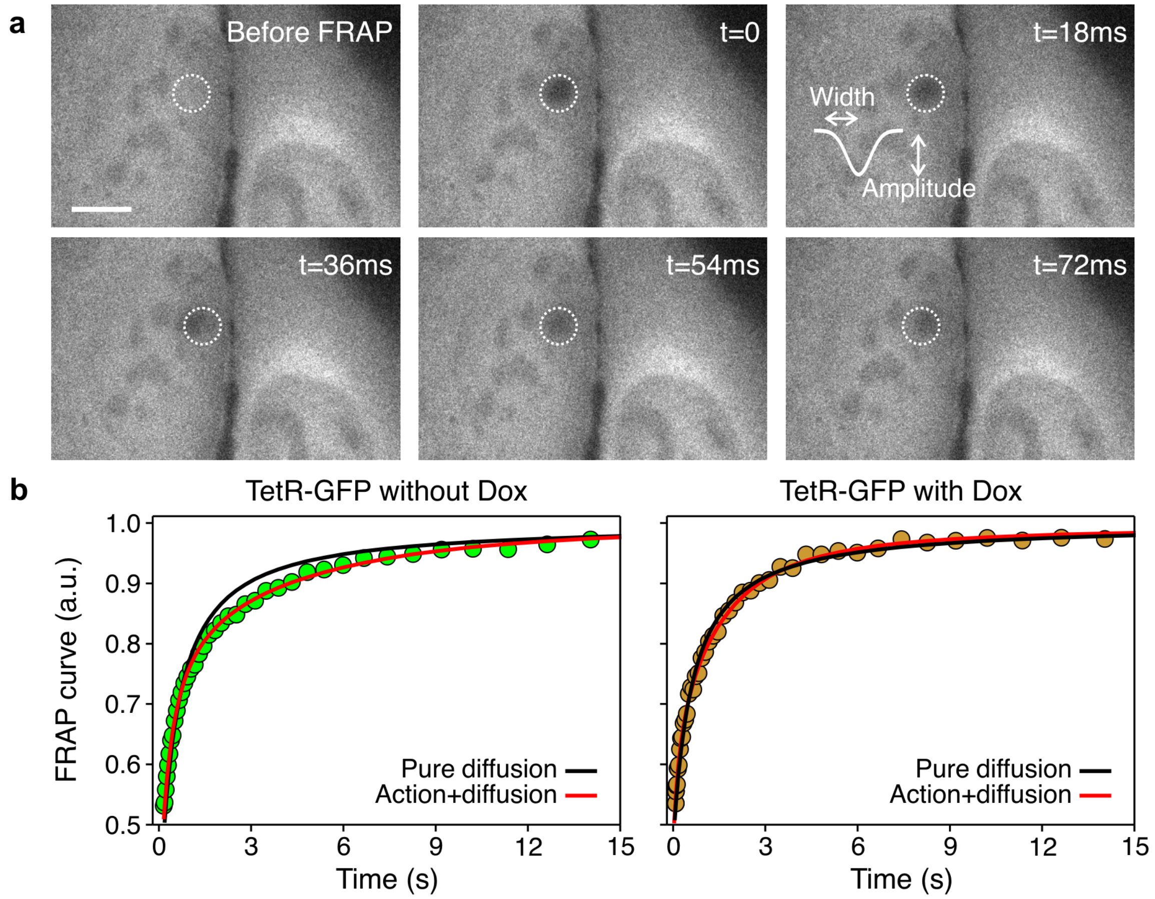

Supplementary Fig. 23 – TetR-GFP nuclear FRAP experiments

(**a**) The figure shows subsequent snap-shots of a U2OS 2-6-3 cells stably expressing TetR-GFP during a FRAP experiment. The dashed circle represent the FRAP region. Scale bar 10 μm. (**b**) TetR-GFP average FRAP recovery curve (circles, error bars fall within symbol dimensions) in the nucleoplasm without Dox (left, N = 15 cells) and with Dox (2.5 μg per ml, right, N = 12 cells). In the absence of Dox, data are best described by an action-diffusion model (red curve). On the contrary, in the presence of Dox, a diffusion model (black curve) is sufficient to describe the recovery data.

In order to extract quantitative information from FRAP data, it is necessary to assume a specific molecular model for protein mobility, *i.e.* pure diffusion, diffusion plus binding, or more elaborated models with several diffusive or interacting populations (27). A way to test if diffusion has to be taken into account or whether it could be neglected is to perform a gradient smoothing test (28), in other words, to look at the temporal evolution of the profile of the bleached region. If the width of the bleach profile (namely the width σ of the Gaussian fit) changes with time, diffusion has to be taken into account, on the contrary if σ doesn't change with time, diffusion can be neglected because occurring on a time scale much faster than that of binding (27,29,30).

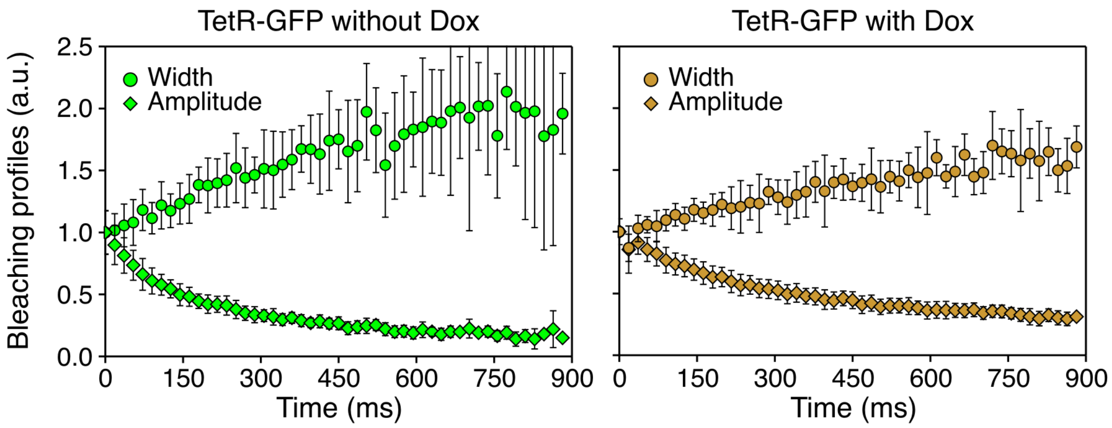

Supplementary Fig. 24 – Bleaching profiles temporal evolution

The graphs show the temporal evolution of the width (circles) and of the amplitude (diamonds) of the bleached region in the absence (left) and in the presence (right) of Dox (2.5 μg per ml final concentration). In both cases, the width of the bleached region increased with time indicating that diffusion plays a role during the recovery process. Error bars represent s.d. and when not visible fall within symbol dimensions.

As shown in Supplementary Figure 24, in our case, the width of the bleaching profile broadens *vs.* time, which implies that a diffusive component is present in both cases, with and without Dox in the cell medium. Thus, to quantify our FRAP data we considered both diffusion and binding. Fitting the TetR-GFP FRAP curve to an action-diffusion model (27) yielded a 20% bound fraction, with an average residence/binding time of 5 ± 3 s (mean ± s.d., N = 15 cells). Furthermore, Dox treatment eliminated the contribution of binding to recovery (Supplementary Fig. 23b) but had no effect on cytoplasmic TetR mobility (Supplementary Fig. 25). The results of the quantification of our FRAP experiments are summarized in Supplementary Table 8. Equations describing the diffusion plus binding model and the pure diffusive model used to fit our FRAP data have been retrieved from (26,31). Altogether, FRAP measurements supported the notion of a search process alternating between transient non-specific interactions with DNA and 3D diffusion.

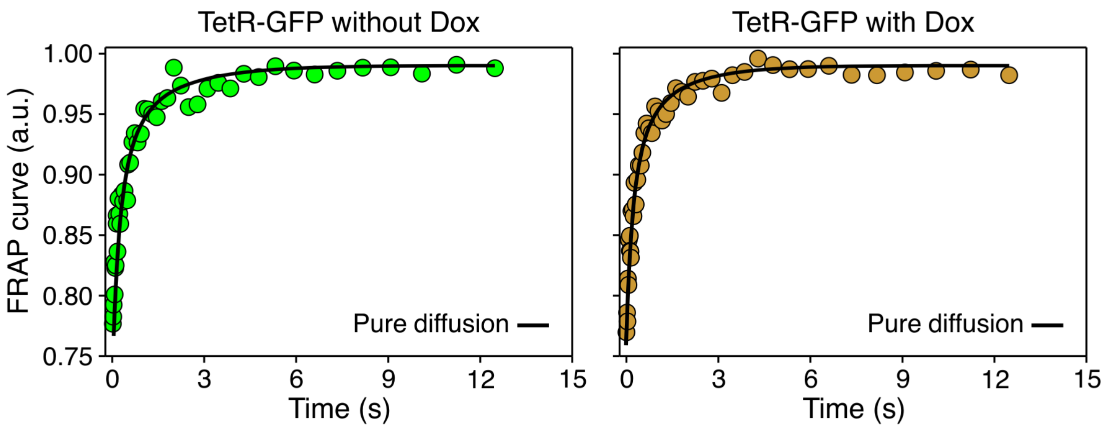

Supplementary Fig. 25 – FRAP experiments in the cytoplasm

FRAP recovery curves in the cytoplasm of cells without Dox (left) and with Dox (right) at 2.5 μg per ml. In both cases, regardless of Dox presence in the cell medium, the recovery curve is well described by a pure diffusive model. Error bars fall within symbol dimensions.

	Condition	Models used	D ($\mu m^2 \cdot s^{-1}$)	Bound fraction	Residence time (s)	Number of cells
Nucleus	Without Dox	Diffusion+ Binding	15 ± 3	0.18	5 ± 3	15
	With Dox	Diffusion	17 ± 6	–	–	12
Cytoplasm	Without Dox	Diffusion	25 ± 2	–	–	12
	With Dox	Diffusion	21 ± 5	–	–	10

Supplementary Table 8 – TetR-GFP FRAP experiments fitting parameters

The table shows the set of parameters obtained by the analysis of the FRAP experiments both in the cell nucleus and the cytoplasm and in the presence (2.5 μg per ml final concentration) and absence of Dox. The bound fraction has been calculated as $k_{\text{off}}/(k_{\text{on}} + k_{\text{off}})$, and the residence time as $(1/k_{\text{off}})$.

Supplementary Note 7

Transient interactions with nonspecific DNA sequences

Running window analysis

In order to discriminate fast transient immobilization events, we used a threshold analysis (32) of the instantaneous diffusion coefficient of individual TetR-Atto647N trajectories. The instantaneous diffusion coefficient D_{Inst} is calculated within a running window of a given number of frames. This, given the intrinsic noise of Brownian diffusion, implies that the smaller the window, the higher the temporal resolution, but the greater the apparent dispersion of the instantaneous diffusion coefficient. To characterize the analysis method and to define the proper value of the threshold, we simulated Brownian trajectories with a known diffusion coefficient and a length of 1000 frames (Supplementary Fig. 26a). Then, we analyzed the simulated traces with a running window (Supplementary Fig. 26b) in order to obtain the variability of D_{Inst} introduced by the short sampling of the trajectory (Supplementary Fig. 26c). The distribution of the instantaneous diffusion coefficient for different sizes of the running window is reported in Supplementary Figure 27a. As expected, the smaller the window the larger the distribution of D_{Inst}.

In order to select the threshold level, we computed the cumulative probability distributions of D_{Inst} for the different window sizes (Supplementary Fig. 27b) and we extracted the D_{Inst} values corresponding to 1, 2.5, 5, and 10% of the C.P.D. Such values are reported in Supplementary Fig. 27c and provide the threshold to use in order to obtain 90 to 99% confidence that events below the threshold really represent deviation from the diffusive behavior and are the benchmark for our transient interaction analysis. Notably the threshold value scales linearly with the Log of the diffusion coefficient D_{Inst} (as obtained comparing simulation for $D = 10\ \mu m^2 \cdot s^{-1}$ and $D = 1\ \mu m^2 \cdot s^{-1}$, see Supplementary Fig. 27b right panels, and Supplementary Fig. 28). This, in turn, gives the possibility to readily obtain the threshold value for every diffusion coefficient and window size (Supplementary Table 9).

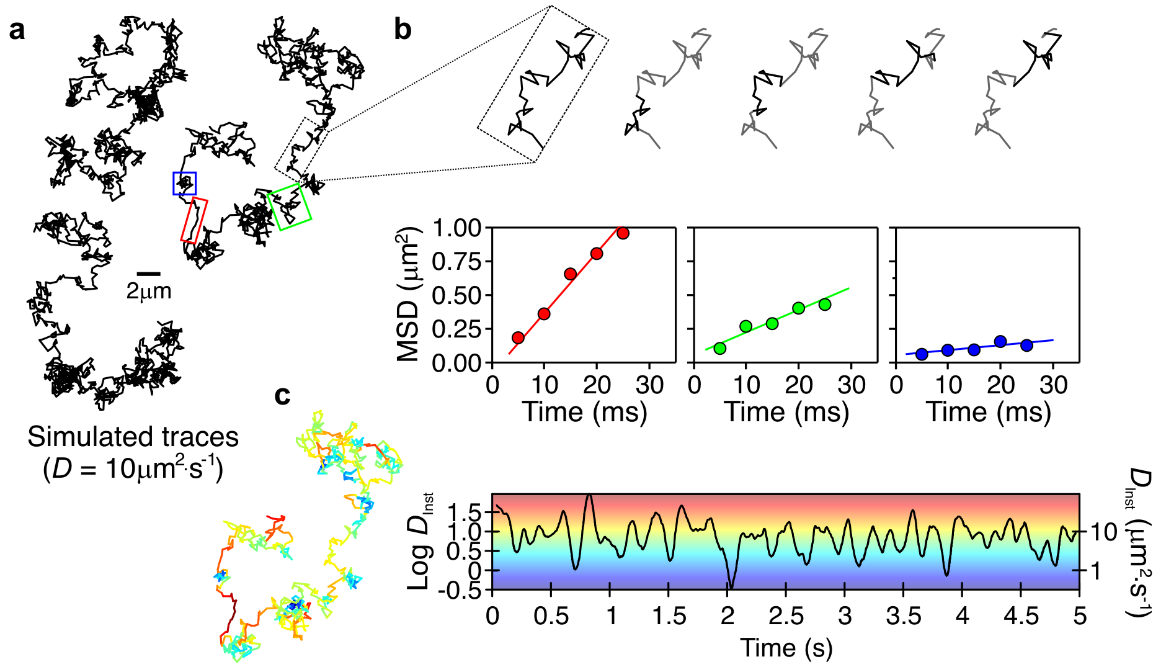

Supplementary Fig. 26 – Running window analysis

(**a**) Example of 1000 frames-long simulated Brownian trajectories in 2D with a diffusion coefficient $D = 10\ \mu m^2 \cdot s^{-1}$. (**b**) Even such traces characterized by a single well-defined diffusion coefficient, when processed with a running window analysis present intrinsic heterogeneity which is reflected by fluctuations of the *D* value. (**c**) Example of running window analysis (16 frames-wide) of one of the trajectories in (a). Color code corresponds to different values of D_{Inst}. The plot on the right shows the value of D_{Inst} along the trajectory.

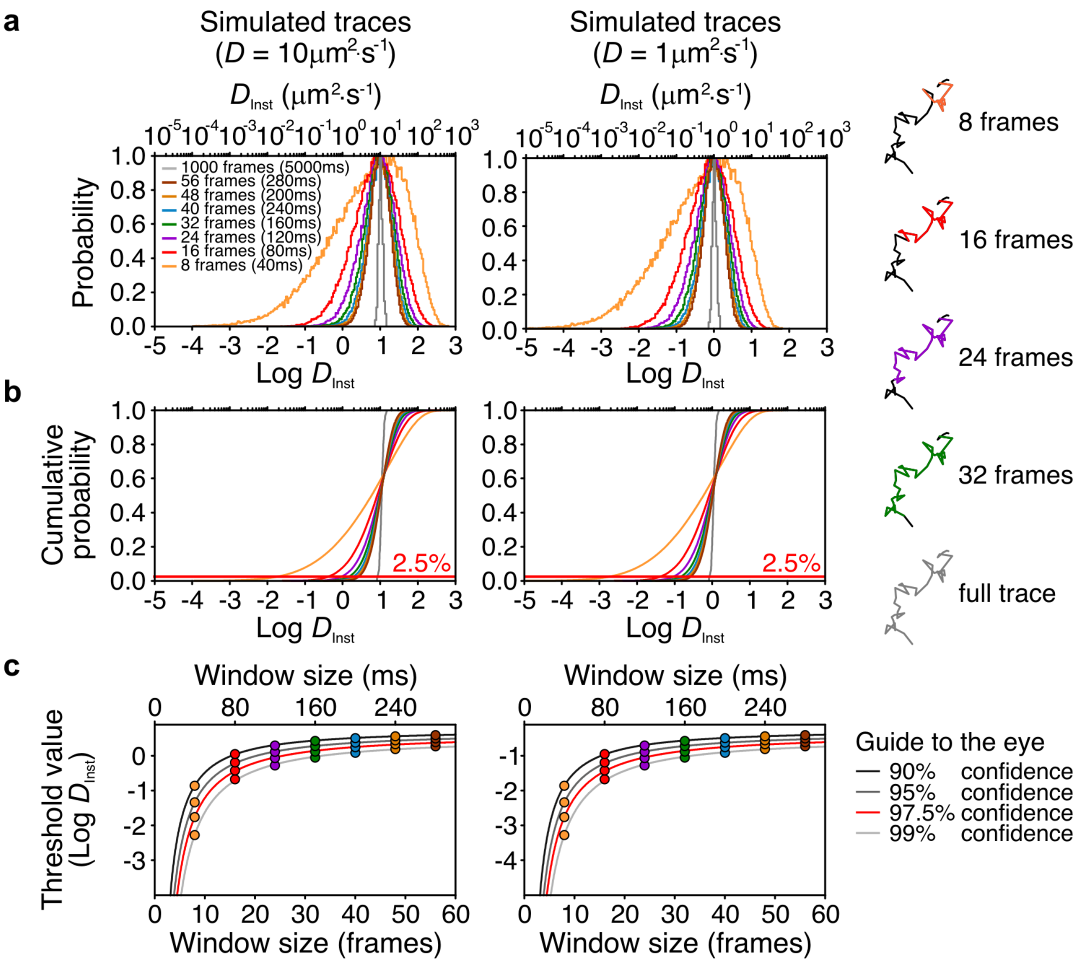

Supplementary Fig. 27 – Threshold determination

(**a**) Probability distribution of D_{Inst} for different sizes of the running window for D = 10 $\mu m^2 \cdot s^{-1}$ (left panels) and D = 1 $\mu m^2 \cdot s^{-1}$ (right panels). (**b**) Cumulative probability of the distributions in (a). (**c**) Thresholds values (Log D_{Inst}) for the different window sizes and different levels of confidence (see also Supplementary Table 9).

Ideally, small window sizes will lead to the best temporal resolution, with a dead time slightly larger than the window size (32). Unfortunately, in our case, for short windows false transitions (33) out of the bound state dominated our analysis resulting always in very short events. We found the best compromise between temporal resolution and robustness of the analysis for a window size of 16 frames, *i.e.* 80 ms in our recordings, in combination with a confidence level of 97.5%.

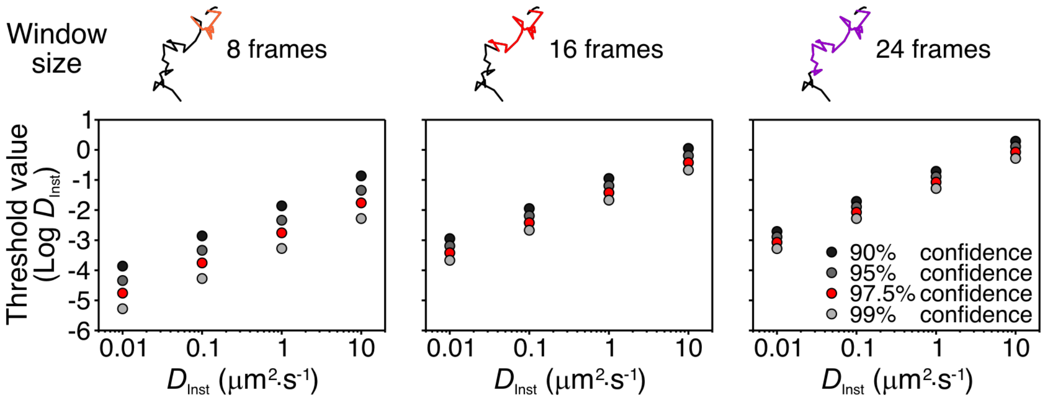

Supplementary Fig. 28 – Threshold values for different window sizes and diffusion coefficients

The figure shows the threshold value for different window sizes (8, 16, 24 frames, corresponding to 40, 80, and 120 ms) and different values for the diffusion coefficient (0.01, 0.1, 1, and 10 $\mu m^2 \cdot s^{-1}$). Dark grey circles correspond to 90% confidence level on the choice of the threshold, grey circles to 95%, red circles to 97.5% (which is the confidence level used in the analysis shown in the main text), and light grey circles to 99% confidence (see also Supplementary Table 9).

D_{Inst} ($\mu m^2 \cdot s^{-1}$)	1			5			10		
Window size (frames)	8	16	24	8	16	24	8	16	24
90% confidence	-1.86	-0.95	-0.71	-1.15	-0.27	-0.02	-0.86	0.05	0.29
95% confidence	-2.34	-1.19	-0.89	-1.65	-0.47	-0.17	-1.34	-0.19	0.11
97.5% confidence	-2.76	-1.42	-1.07	-2.07	-0.72	-0.37	-1.76	-0.42	-0.07
99% confidence	-3.28	-1.67	-1.28	-2.57	-0.96	-0.57	-2.28	-0.67	-0.28

Supplementary Table 9 – Threshold values for running window analysis

The table summarizes the values of the threshold for different diffusion coefficients, window sizes, and confidence levels.

Time-lapse experiments

After quantifying the very transient interactions thanks to the running window analysis of the continuous imaging traces (nonspecific binding time constant 150 ms), in order to estimate the long-residence time of nonspecific binding events, we performed time-lapse experiments acquiring snapshots (5 ms integration time) of the cell with different inter-frame time (τ_{TL}) in order to extend our observation windows to longer times preventing the bleaching to mask the long events.

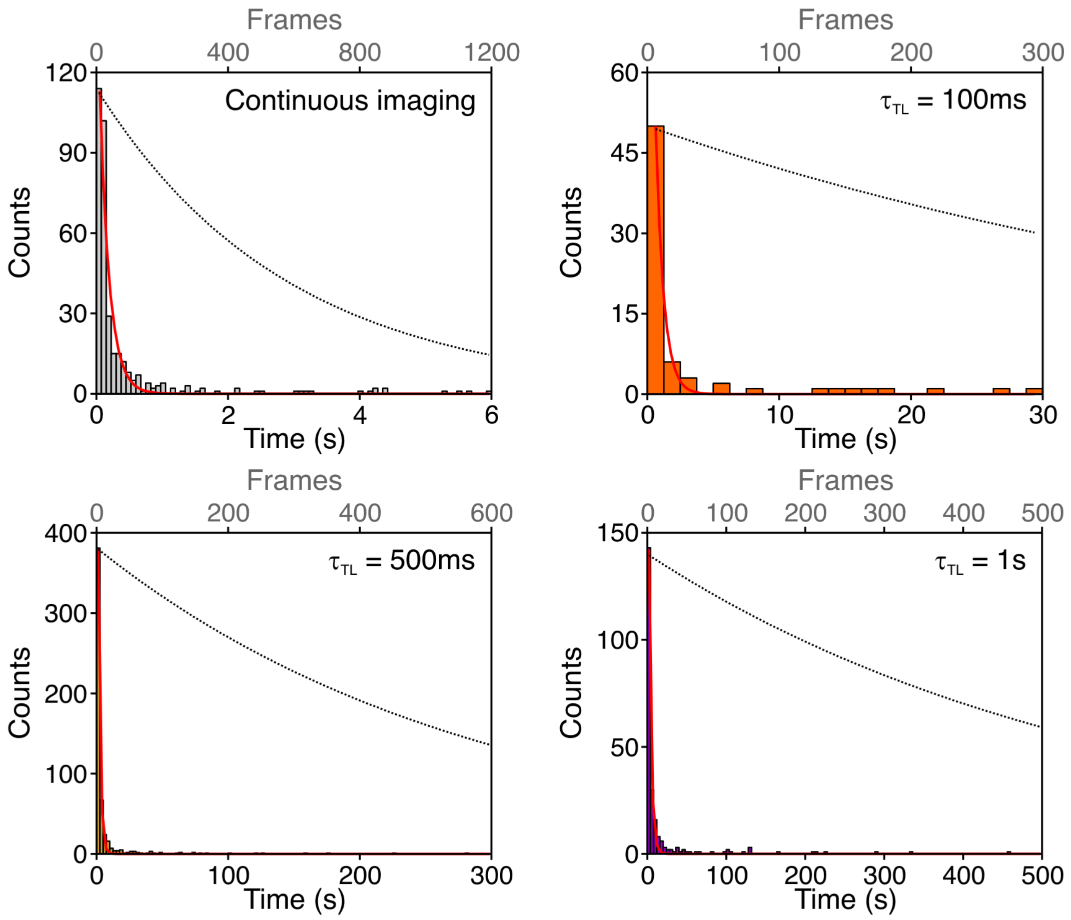

Supplementary Fig. 29 – Duration of the nonspecific binding events

The figure shows the histograms of the duration of the nonspecific binding events observed in the different experimental conditions (τ_{TL} = 5 ms, 100 ms, 500 ms, and 1 s). Red curves are single exponential fits to the experimental distributions and the black dotted lines represent the bleaching properties observed for TetR-Atto647N ($\tau_{Bleaching}$ = 2.9 s or 580 frames under continuous imaging in our experimental conditions, see Supplementary Note 4 and Supplementary Fig. 16b). Fitting parameters are reported in Supplementary Table 10.

In particular, we sample the position of TetR-Atto647N protein in the nucleus 10, 2 and 1 time per second. We localized particles as for the continuous imaging (parameters reported in Supplementary Table 4, but we used a very low expected diffusion coefficient to specifically track immobile molecules and a blinking value of 8). We considered as immobile a protein that remained within our pointing accuracy for at least 2 frames ($1\Delta T$). The distributions of the duration of the nonspecific binding events are reported in Supplementary Figure 29.

	Continuous imaging	100 ms	500 ms	1 s
τ (s)	0.148 ± 0.008	0.62 ± 0.04	1.51 ± 0.02	2.86 ± 0.07
N events	353	70	541	237
N cells	5	1	2	2
Scaling factor	1	0.275	0.195	0.0975
Average bound fraction	0.25	0.18	0.22	0.27

Supplementary Table 10 – Fitting parameters of the distribution of the duration of the nonspecific binding events.

The table shows the values of the decay constants τ (± the s.d.) obtained with a single exponential fitting of the distribution of the non-specific binding times (shown in Supplementary Fig. 29) for the different experimental conditions (continuous imaging and time-lapse experiments). *N* events and *N* cells report on the data set used for the analysis; the scaling factor is the value used to scale the SP (Fig. 3c of the main text and Supplementary Fig. 30). The average bound fraction represents the mean value of proteins bound to non-specific sites per cell nucleus (Supplementary Fig. 31).

Global distribution of non-specific interaction times

Next, in order to obtain the global distribution of the residence time on nonspecific DNA sites, we calculated the survival probability (SP), *i.e.* the probability of staying bound longer than a time τ (or in other words the inverse cumulative probability of binding times). The survival probabilities for the different τ_{TL} are shown in Supplementary Fig. 30a. First, they were rescaled (*i.e.* multiplied by a scaling factor, see Supplementary Table 10 for the re-scaling values) In order to trace the global nonspecific residence time survival probability shown in Fig. 3c of the main text, The value of the scaling factors was determined by superimposing the different curves at $t = 1$ s. Next, we calculated the integral value of the global nonspecific residence time survival probability in order to estimate the mean nonspecific residence time. The dataset used for the calculation is shown in Supplementary Fig. 30b and the integral value obtained is τ_{1D} = 2 s. Finally, we calculated the exponent of the power law which best describes the behavior of the global nonspecific residence time survival probability by fitting all the data points corresponding to the power law regime (dataset shown in Supplementary Fig. 30c). The value γ obtained for the power law exponent is -0.69 ± 0.01.

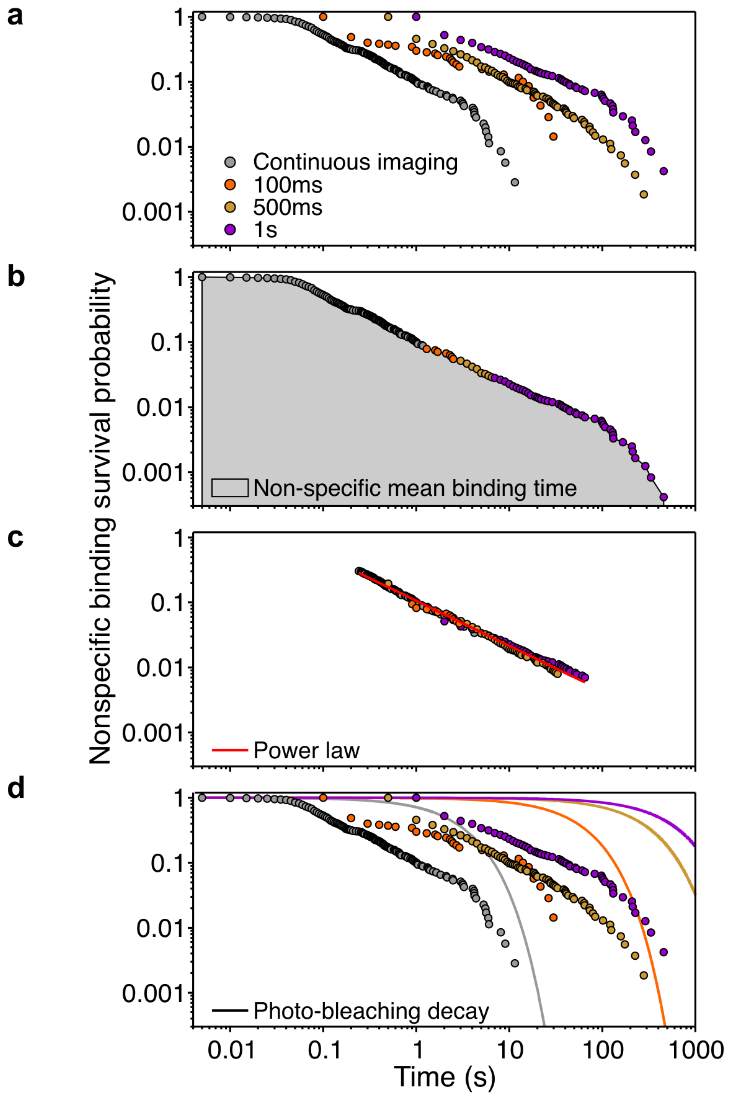

Supplementary Fig. 30 – Nonspecific binding events survival probability analysis

(**a**) Survival probability calculated for the different data sets. Grey circles: continuous imaging, orange circles, τ_{TL} = 100 ms; brown circles, τ_{TL} = 500 ms; purple circles, τ_{TL} = 1 s. (**b**) Global survival probability (black line) after rescaling the different τ_{TL} data sets (colored circles), the grey area indicates the mean nonspecific binding time, which is estimated to be 2 s. (**c**) Data set from the different τ_{TL} experiments used to calculate the power law behavior (red line), which extends over three logs. The value of the exponent γ obtained is -0.69 ± 0.01. (**d**) The graph shows the photobleaching limit (continuous lines), calculated considering $\tau_{Bleaching}$ = 580 frames (see Supplementary Fig. 16) and the frame rate used in the different imaging conditions, and overlapped to the experimental data (circles). Grey: continuous imaging, orange: τ_{TL} = 100 ms, brown: τ_{TL} = 500 ms, purple: τ_{TL} = 1 s.

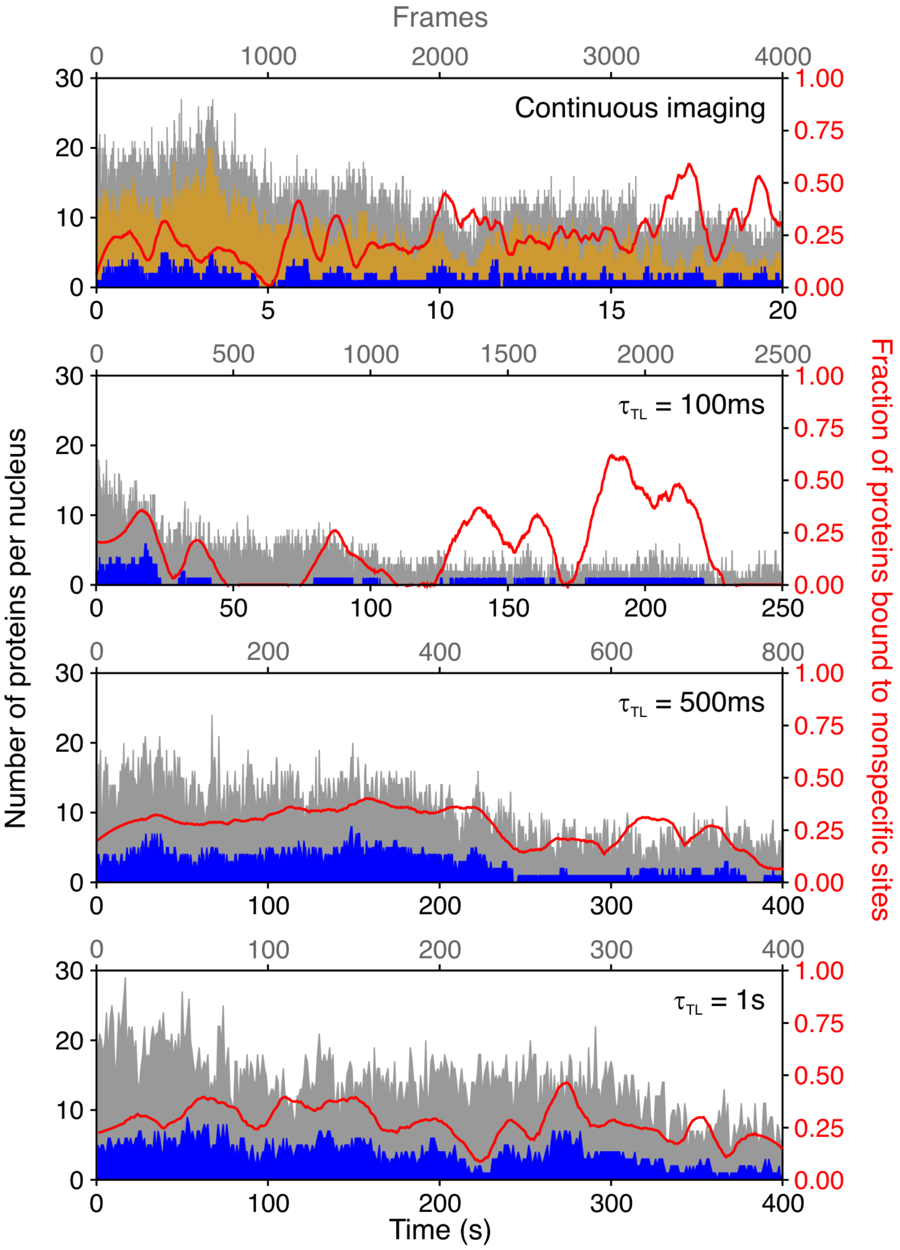

Supplementary Fig. 31 – Time-course of nonspecific binding events

The figure shows the time course of the number of nonspecific binding events for individual cells for the continuous imaging (top panel) and for the different time-lapse experiments (from second to fourth panel, τ_{TL} = 0.1, 0.5, and 1 s). Grey lines represent the total number of individual proteins detected per nucleus; the blue lines the number of TetR-Atto647N proteins bound to nonspecific sites. The red lines represent the ratio between the number of proteins bound to nonspecific sites and the total number of detected proteins; despite some fluctuations, the fraction of nonspecifically bound proteins remains in the range between 20 and 25% (Supplementary Table 10). The brown line in the top panel represents the number of proteins that have been reconstructed in individual trajectories. The ratio between the number of trajectories reconstructed and the total number of detected proteins is about 80%.

As visible in Supplementary Fig. 30d, with the exception of continuous tracking, in all the other imaging conditions (time-lapse experiments) we are not limited by photobleaching. The deviation of the last points and the truncation of the power-law behavior at very long non-specific binding time should be interpreted, to our view, as the upper reachable limit of the DNA-binding time given by supposedly maximal TetR affinity for the DNA (*i.e.* for the *tet*O sequence, yellow line in Fig. 3c of the main text), which acts as a cut-off in the power-law distribution.
To estimate the confidence interval on our estimation of τ_{1D}, we have numerically computed the difference in the area under the power law distribution (within the experimentally observed temporal range) in the case of the maximal and minimal power law exponents estimated from the data. This gave a 2.5% error on the computation of the mean bound time τ_{1D}.

Microscopic hopping cannot explain the power law distribution of nonspecific binding times

Given the finite spatio-temporal resolution (~25 nm and 10 ms) of our imaging system, it is not possible to rule out microscopic events in which the protein detaches and rebinds at a nearby site (over even at the same site). Notably, such a problem is also present in *in vitro* protein-DNA interaction studies and has been discussed by several authors (see Ref. 34, for instance). In our case, we specifically examine if microscopic hopping (with a single fast unbinding rate) might explain the power-law distribution of binding times, meaning that the distribution has a geometric origin rather than a biochemical one.

First, we modeled the dynamics of a random walker and assumed that when a binding event is observed, microscopic excursions within an observation sphere of radius *R* (typically *R* ~ 50 nm) cannot be resolved. The observed binding time is thus defined as the first passage time of the protein to the sphere boundary (often call exit time from the sphere). We assumed that *N* identical binding sites (of radius *a*) are located within the observation sphere, each inducing a microscopic mean binding time denoted τ_{MB}. The protein diffuses within the array with diffusion coefficient *D*, and can bind and unbind to several binding sites before eventually exiting the sphere. In fact, since the observation domain is bounded, general mathematical arguments show that the distribution of the exit time from the sphere has an exponential tail and cannot be power law distributed (provided that the microscopic binding events are not power law distributed). Importantly, this holds for any distribution of binding sites within the sphere, including the case of a linear array. This is more easily seen in the regime $N \gg 1$. If one assumes for simplicity a homogeneous distribution of binding sites within the sphere (as stated above other distributions would not change the asymptotic behavior of the survival probability), the motion of the protein inside the sphere can be approximated as a usual Brownian motion of effective diffusion coefficient:

$$D_{\mathrm{Eff}} = \frac{\tau_{\mathrm{F}}}{\tau_{\mathrm{F}}+\tau_{\mathrm{MB}}} \ , \tag{5}$$

where the mean time between successive binding events is given by $\tau_{\mathrm{F}} = R^3/(3NaD)$. Denoting by $S_{\mathrm{t}}(r)$ the probability that the protein, which started from *r*, has not left the sphere until time *t*, the dynamics can be written under such hypothesis (35):

$$\begin{array}{c} \partial_{\mathrm{t}} S_{\mathrm{t}}(r) = D_{\mathrm{Eff}}\, \Delta S_{\mathrm{t}}(r), \quad r < R \\ S_{\mathrm{t}}(R) = 0 \end{array} \ . \tag{6}$$

The problem can therefore be simply rephrased as the determination of the exit time distribution from a sphere for a Brownian particle, which is well known to be exponentially distributed at long times (35).

From an experimental point of view, we observed that the non-specific association rate is low, such that it does not favor (but does not rule out) a scenario where proteins locally undergo multiple unbinding/rebinding events.

Overall, the theoretical arguments above lead us to rule out a purely geometrical explanation for the power-law distribution of binding times. That being said, our interpretation in terms of variability of the binding sequences is only a plausible scenario and is not fully demonstrated in the paper.

Supplementary Note 8

Measurement of the association rate constant *in situ*

Limits of the TetR assay for measuring the association rate constant

There are several technical reasons that make the binding kinetics of TetR at the target site difficult to observe and quantify.

In the case of injection at high concentration (see Fig. 1c of the main text), the recruitment at the locus is relatively rapid but it is difficult to give a quantitative estimate of the search kinetics. First, we do not have a very precise control of the concentration of proteins (and, thus, of the abundance of searchers) injected in each nucleus, also because of the variability of the nuclear volume from cell to cell. Second, upon injection, there is a significant time lag (~1 minute) before the fluorescence signal of the injected proteins could be collected. This is due to the fact that after injection we needed to (i) change the optical path and the imaging filters of the microscope; (ii) check the focus of the microscope and, eventually, readjust it; and (iii) retract the injection needle from the proximity of the cell and wait for the molecules released outside the cell to diffuse away. In fact, the injection tip is always kept under a (low) "compensation" pressure (in order to avoid the clogging of the aperture and the entering of the cell medium inside the injection needle), and, thus, it releases continuously a little amount of fluorescent proteins in the medium surrounding the cell.

In the case of experiments in the single-molecule regime, normally, after injection, we observed a few molecules bound at the target locus (see top and bottom right images in Fig. 2a of the main text and Supplementary Fig. 16a), which sequentially bleached with a stepwise signal (Supplementary Fig. 16a and Supplementary Fig. 32). Occasionally, we observed single TetR proteins associating to the target site (green box in Supplementary Fig. 32), however binding events were very rare (for instance, in the ~15 minutes-long experimental recording of the TetR-Atto647N fluorescence intensity at the specific target locus reported in Supplementary Fig. 32, we could detect only one binding event). Also, we observed more proteins diffusing through the array of binding sites without binding (blue events in Supplementary Fig. 32) than proteins stably associating to the array (green event in Supplementary Fig. 32). All these lines of evidence are qualitatively consistent with the low *in vivo* binding efficiency discussed in the manuscript, nevertheless, the overall exiguity of the events recordable in suitable conditions for single-molecule detection in mammalian cells prevents a statistically significant quantification of binding *vs.* non-binding proteins as well as of the TetR association rate constant.

For all these reasons, we reverted to the inducible protein RevTetR that allowed a very accurate measurement of the association rate constant. We would like to emphasize the importance of the experiments on the association kinetics of RevTetR. To our knowledge, this is one of the first times that reaction kinetics and concentration of reactants have been correlated at the single cell level.

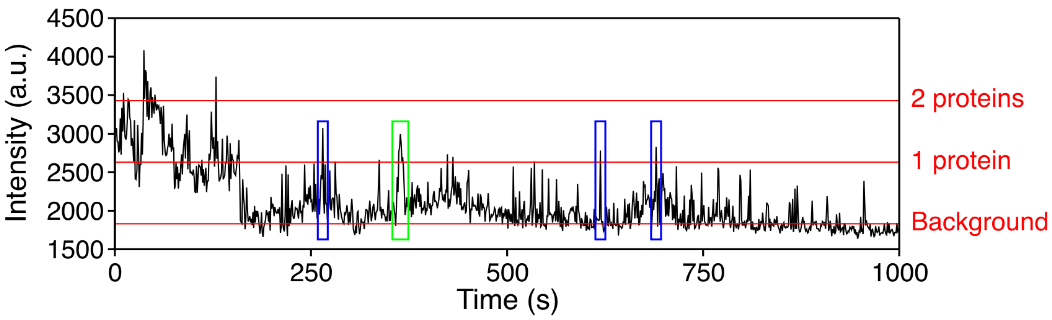

Supplementary Fig. 32 – Time course of TetR-Atto647N proteins at the target locus

Fluorescence intensity of TetR-Atto647N at the target locus recorded in a time-lapse mode (one 5 ms-exposure image per second). Initially, there are two TetR-Atto647N proteins bound at the target locus and their signal photobleaches in a stepwise manner. Subsequently (after ~200 s), we observe fluctuations of the signal at the locus. Very rapid spikes in the signal (events marked in blue) are attributed to proteins diffusing through to the target locus but not stably associating to the binding sites, thus visible in the recording only in one frame before diffusing away. Sporadically, a protein associates to the target locus and its signal persists for a few seconds, limited by photobleaching or dissociation (event marked in green).

We think that our approach opens new prospects for *in situ* biochemical measurements and it should be valuable in many other contexts by bridging the gap between our understanding of the kinetics of biochemical reactions *in vitro* and *in vivo*.

Two-photon FCS measurements

In order to directly measure the association rate constant of RevTetR-GFP to the target locus, we measured both the concentration *c* of RevTetR-GFP in each cell and the observed association rate k_{Obs}. In particular, to measure protein concentration in the cell nuclei we used two-photon FCS. Experiments have been performed on a previously described experimental set-up (36,37). Briefly, we used an inverted Olympus IX81 microscope and we created an observation volume of ~0.5 fL (Supplementary Fig. 33) within the cell nuclei by focusing a tunable, mode-locked Ti:Sa laser (Chameleon Ultra II, Coherent, USA), operated at 940 nm, with a 60X N.A. 1.2 NIR water immersion objective (UplanSApo 60XW, Olympus, France). Fluorescence signal was detected with a fiber-coupled (100μm-core, multi-mode fiber, AFS105/125Y, Thorlabs, USA) avalanche photo-diode (SPCM-AQRH-14-FC, Perkin-Elmer, Canada) and fed to an external digital correlator (Flex03LQ-01, Correlator.com, USA). The signal autocorrelation $G(\tau)$ was fitted with purely diffusive model to determine the mean number of molecules in the excitation volume as $\langle N_{Mol} \rangle = 1/G(\tau \rightarrow 0)$ (38). Three FCS measurements (each composed of three 30 s-long acquisitions) were performed in three different nuclear locations to estimate intra-nuclear heterogeneity in protein concentration (*x* error bars in Fig. 4c of the main text) avoiding to point on the binding site locus and on nucleoli.

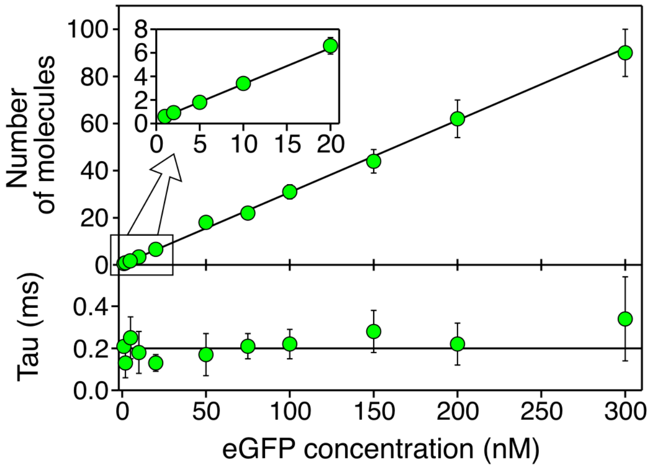

Supplementary Fig. 33 – Two-photon FCS calibration with eGFP

In order to calibrate the observation volume of our two-photon FCS system, we used different dilutions of known concentration of eGFP in CHES buffer (50 mM, pH 9.5, 100 mM NaCl) with 0.1 mg per ml BSA (29130 Thermo-Scientific). We recorded FCS curves, which were fitted as previously described, in order to extract the average number of proteins in the detection volume (green circles), which is plotted as a function of the eGFP concentration. From a linear fit of the data (black line) we derived our two-photon confocal volume to be about 0.5 fL. The inset in the graph shows a zoom in of the calibration curves at low eGFP concentrations. The bottom graph shows that, as expected, the average residence time Tau of eGFP in the observation volume does not vary as a function of the concentration. When not visible, error bars fall within symbol dimensions.

Importantly, RevTetR concentration was measured prior to induce protein association by Dox. In this condition, our sptPALM data on RevTetR-Dendra2 in the absence of Dox show that the percentage of moving proteins (thus accounted in our FCS measurement) is about 90% (Supplementary Note 5 and Supplementary Fig. 22 top panel). Furthermore, RevTetR-GFP concentration was measured acquiring and averaging three 30 s-long FCS traces in 3 different nuclear locations. Assuming that the distribution of nonspecific binding times of RevTetR does not differ much from that of TetR (39) shown in Fig. 3c of the main text, only 2% of the proteins undergoing to nonspecific interactions (which are 10% of the total) would appear as immobile on the time-scale of our FCS measurements.

RevTetR-GFP association experiments

Association of RevTetR-GFP upon Dox induction was monitored acquiring 3D stacks (usually 5 planes with 1 μm separation) every 5 or 10 seconds. Wide-field images have been recorder with an intensified CCD (HQ^2 CoolSNAP, Roper Scientific, Germany) with 100 ms pulsed blue LED (M470L2, Thorlabs, USA) illumination. Binding site intensities have been quantified using a MatLab 3D localization and

intensity measuring routine (FISHquant, Ref. 40) and fitted with single exponential curves to extract the observed association rate constant k_{Obs}.

We tested several different concentration of Dox in our recruitment experiments to rule out the possible contribution due to Dox permeation inside the cell. In particular we varied the concentration of Dox from 2,5 μg per ml up to 125 μg per ml without reporting any change in the recruitment kinetics (Supplementary Fig. 5).

Site-specific chromatin de-compaction with VP16 domain

To prove the de-compaction and opening of the target locus under the action of the trans-activator domain VP16 (4), we checked for the transcription of the reporter gene (CFP-SKL) of the artificial gene array. As shown in Supplementary Fig. 6, after overnight transfection of U2OS 2-6-3 cells (stably expressing RevTetR-GFP) with the NLS-LacI-mCherry-VP16 plasmid, we could clearly detect fluorescent spots (Supplementary Fig. 6, bottom right panel) in the cytoplasm of cells and corresponding to induced expression of the CFP-SKL reporter gene.

Steady-state occupation of the target locus

In order to estimate the steady-state occupation of the binding locus in the U2OS 2-6-3 and the U2OS 4A cell lines, we measured with an epi-fluorescence microscope the fluorescence intensity of RevTetR-GFP at the target site locus (Supplementary Fig. 34) long after Dox induction (30 minutes of Dox treatment at 2.5 μg per ml final concentration).

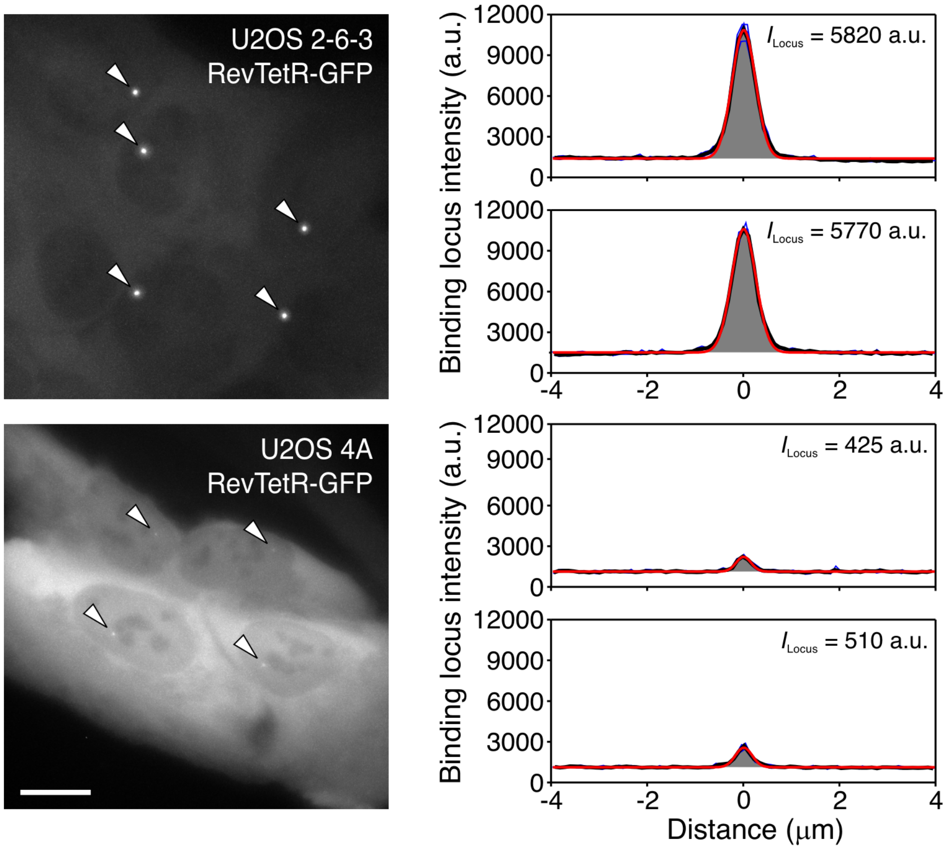

Supplementary Fig. 34 – Binding site locus intensity analysis

Left panel: The picture on the top shows a representative field of view of U2OS 2-6-3 cells and the one on the bottom of U2OS 4A cells, white arrows indicate the target loci. Images are maximal intensity projection of 17 different vertical slices taken every 250 nm with 50 ms exposure time for the U2OS 2-6-3 cell line and 400 ms exposure time for the U2OS 4A cell line, under the same illumination conditions. Scale bar represents 10 μm. Right panels: Two representative line intensity profiles for the U2OS 2-6-3 cell line are shown on the top, while two representative line intensity profiles for the U2OS 4A cell line are shown on the bottom. The locus intensity has been estimated by plotting 4 different line intensity profiles per each locus (blue curves) and fitting the average profile (black curve) with a Gaussian function (red curve) to estimate the total intensity of the binding locus (*i.e.,* the area of the Gaussian represented in grey). Given the different exposure times, to compare the data, the integrated intensity for the U2OS 4A cell line has been later divided by 8 to normalize it at the same exposure time than for the U2OS 2-6-3 cell line.

Supplementary Note 9

Super-resolution imaging of the target site

Super-resolution images of the different binding site loci have been performed using a PALM microscope equipped with an adaptive optical system (MicAO™, Imagine Optics, France) (41). In particular, we used a deformable mirror (MirAO 52-e, Imagine Optics, France) to shape the point spread function (PSF) of our imaging system (42) in order to correct for aberration (43), achieve a higher signal to noise ratio (and, thus, a higher localization accuracy) (44) and to induce a controlled degree of astigmatism to the PSF. Astigmatism is used to break vertical symmetry of the imaging system and obtain information on the 3D position of molecules within the focal depth of the microscope (~600 nm) (45,46).

Briefly, we used an inverted microscope (Ti Eclipse, Nikon, France) equipped with a perfect focus system (Nikon, France), a metal-halide lamp (Nikon, France) in combination with a filter wheel (Lambda 10-3, Sutter Instruments, USA) for regular fluorescence imaging. For single-molecule imaging we employed a 561 nm imaging laser (Genesis MX 561-2000 MTM, Coherent, USA) and a 405 nm activation laser (Cube 405-100C, Coherent, USA) focused in the back focal plane of a 100X N.A. 1.49 oil immersion objective (CFI Apo TIRF 100X, Nikon, France) and a 512 × 512 EM-CCD (Ixon3 DU897, Andor, Ireland) to acquire images. All instrumentation was controlled via NIS Elements (Nikon, France). The adaptive optical system is placed in the detection pathway of the microscope. Besides the deformable mirror, it includes a wave-front analyzer and an extra 1.5X lens. The resulting pixel size on the CCD is 106 nm.

First, we optimize the shape of the mirror in order to correct for aberration and obtain the best possible collection efficiency using as point sources 200 nm fluorescent polystyrene beads (Tetraspeck™ T7280, Molecular Probes, Invitrogen). Next, we added 0.06 of astigmatism to the PSF and we acquired several calibration curves on different beads in a range of 1μm around the focal position with steps of 12 nm using a piezo stage (Nano-Z100, Mad City Labs Inc., USA, driven by a closed loop controller, Nano-Drive, Mad City Labs Inc., USA). The acquired images have then been fitted with a two-dimensional Gaussian function with independent widths (σ_X and σ_Y) using a MatLab routine derived from MTT (16). The resulting calibration curves are averaged and fitted with a polynomial function of the third order to extrapolate the relation between the difference of the widths of the Gaussian fit and the vertical position (normally, achieving a depth of focus of the order of 600 nm).

After shaping the PSF and calibrating the vertical detection, we acquired PALM images of several cells. Cells were plated on plasma-cleaned coverslips overnight, transfected for 24 hours and then fixed with 4% para-formaldehyde for 15 min at room temperature before imaging. 200 nm fluorescent polystyrene beads (Tetraspeck™ T7280, Molecular Probes, Invitrogen, USA) are added on top of the

cells at 1:100 dilution during 3 to 5 minutes are and used as fiduciary markers to correct for drift during imaging. 3D super-resolution images were generated using between 80,000 and 200,000 frames acquired with an exposure time of 25 ms, the gain of the EM CCD set to 300, and under continuous illumination with a density of energy of ~4.5 kW per cm^2 for the 561 nm imaging laser and between 0 up to ~10^2 kW per cm^2 for the 405 nm activation laser. Super-resolution Images have been visualized, analyzed, rendered, and animated (Fig. 4d of the main text and Supplementary Movie 9) with the software ViSP (47).

Supplementary Note 10
Modeling of the search kinetics

Diffusion *vs.* reaction limited kinetics

In this section we compute the association rate constant of a protein to a specific target site, and take into account the specific 3D organization of the targets.

We assume for the sake of simplicity that the N target sites (of radius a) are uniformly distributed within the locus, modeled as a sphere of radius r_t. The locus is assumed to be centered in a spherical nucleus of volume V, and the protein position is denoted by r in usual spherical coordinates. Outside the locus ($r > r_t$), we take into account effectively the non specific binding events, and consider that the protein performs a $3D$ diffusion with diffusion coefficient $D^* = D_1 \cdot \tau_{3D}/(\tau_{1D} + \tau_{3D})$. In the relevant regime $N \gg 1$, one can assume for $r < r_t$ that the protein binds to a specific target site with probability per unit time κ, which will be calculated below. Denoting by $S_t(r)$ the probability that the protein, which started from r, has not bound to a target site until time t, the dynamics can be written under such hypothesis (35):

$$\partial_t S_t(r) = D^* \Delta S_t(r), \quad r > r_t \tag{7}$$

$$\partial_t S_t(r) = D^* \Delta S_t(r) - \kappa S_t(r), \quad r < r_t. \tag{8}$$

The mean binding time $T(r)$ to any of the target sites is then classically given by $T(r) = \int_0^\infty S_t(r)dt$, which satisfies:

$$-1 = D^* \Delta T(r), \quad r > r_t \tag{9}$$

$$-1 = D^* \Delta T(r) - \kappa T(r), \quad r < r_t. \tag{10}$$

The exact solution of this linear ordinary differential equation is obtained by standard tools, and reads for $r > r_t$ in the limit of large volume V:

$$T(r) \simeq \frac{V}{4\pi D^* r_t} \left(\left(1 - \frac{\tanh(r_t\sqrt{\kappa/D^*})}{r_t\sqrt{\kappa/D^*}} \right)^{-1} - \frac{r_t}{r} \right), \tag{11}$$

which yields the following association rate:

$$k_a \simeq 4\pi D^* r_t \left(1 - \frac{\tanh(r_t\sqrt{\kappa/D^*})}{r_t\sqrt{\kappa/D^*}}\right). \tag{12}$$

We now determine κ. In the regime $N \gg 1$, it is easily seen that $\kappa \simeq \theta^{-1}$, where θ is the mean binding time to a single target of radius a for a protein diffusing in a unit volume $v = 4\pi r_t^3/(3N)$. In order to take into account the binding efficiency to a specific target site, we introduce λ, which is the probability per unit time that binding occurs if the distance between the protein and the target is smaller than a. The regime $\lambda \to \infty$ then corresponds to perfect reaction (and $\lambda \to 0$ to low binding efficiency). Under these hypothesis, it is clear that θ solves the system (equations 7 and 8), with the substitution $V \to v, r_t \to a, \kappa \to \lambda$. One then gets:

$$\kappa \simeq \frac{4\pi D^* a}{v}\left(1 - \frac{\tanh(a\sqrt{\lambda/D^*})}{a\sqrt{\lambda/D^*}}\right). \tag{13}$$

Eqs. (12,13) then explicitly determine the reaction rate.

Diffusion limited regime

We first assume that binding is efficient, *i.e.* $\lambda \gg D^*/a^2$. In this regime, one has $\kappa \simeq 4\pi D^* a/v$. Using that $r_t\sqrt{\kappa/D^*} = (3aN/r_t)^{1/2} \gg 1$ for the experimentally relevant parameters (see below), we find that the reaction rate reduces to $k_a \simeq 4\pi D^* r_t$. This shows that the density of target sites inside the target locus is so high that, if the specific binding is efficient, the target locus can be considered as an absorbing sphere of radius r_t. Using the experimental parameters, this estimate yields $k_a \simeq 10^9\ \mathrm{M}^{-1}\cdot\mathrm{s}^{-1}$, which is far higher than the measured rate. This shows that the TS cannot be mediated by 3D diffusion only with a perfect association : the limiting step of the reaction is not the diffusive transport in the nucleoplasm.

Reaction limited regime

We now assume that binding efficiency is low, *i.e.* $\lambda \ll D^*/a^2$. In this regime, one finds $\kappa = \lambda N a^3/r_t^3$, as seen from Eq. (13). Noting that $r_t\sqrt{\kappa/D^*} \ll 1$, one obtains from Eq.(12) that $k_a \simeq \lambda N(4/3)\pi a^3$. Note that in this so-called reaction-limited regime, the association rate is independent for the diffusion coefficient D^*, and merely proportional to the local equilibrium concentration of proteins at the target sites. In particular it is proportional to N irrespectively of the 3D organization of target sites, provided that the reactivity of each target site is independent of the other target sites. Yet, the comparison of the

rates obtained with U2OS 2-6-3 and U2OS 4A cell lines rules out this hypothesis. While the equilibrium concentration is indeed found to scale linearly with N, this is clearly not the case for the association constant k_a. The observed effective interactions between neighboring targets calls for an alternative mechanism.

Facilitated diffusion hypothesis

Motivated by the observation of non specific binding to DNA, we finally suggest that TS involves not only $3D$ diffusion, but also sliding. In fact, taking into account the estimated DNA concentration c_{DNA} indicates that the non-specific binding rate $1/\tau_{3D}$ is very low (much smaller than expected for perfect association). This shows that non-specific binding is not limited by 3D diffusion, but by binding. We will assume here that non-specific binding to DNA is the limiting step of the reaction, which is consistent with the analysis above. Following the classical picture of facilitated diffusion, we assume that, once non-specifically bound to DNA, the protein can perform 1D diffusion along the DNA strand and explore the neighboring sequences. We denote by l_{SL} the mean number of bp scanned during a sliding event (antenna effect). In the relevant regime where l_{SL} is larger than the distance between targets within an insert ($23\ \mathrm{bp}$), all targets of an insert cannot be considered as independent. In fact, we will assume that l_{SL} is large enough, so that each insert should be considered as an individual target. We last denote by p the probability that binding to a specific target occurs if the sliding excursion overlaps with an insert. Following and generalizing classical facilitated diffusion models (48), the association rate constant can then be written:

$$k_a \simeq \frac{l_{\mathrm{SL}}}{c_{\mathrm{DNA}}} \frac{N_i}{\tau_{1D} + \tau_{3D}} \cdot p. \tag{14}$$

Using the experimental values (see below), the observed value of k_a is recovered provided that $p \simeq 1$. In addition, this scenario of facilitated diffusion is also compatible with the observed dependence on the target radius r_t, number of target sites N and inserts N_i.

Definition of parameters and experimental observations

- U2OS 2-6-3 cell line: $N_i = 200$ inserts for a total of $N = 19200$ target sites. The effective size of a target site is denoted by $a \simeq 5\ \mathrm{nm}$. The locus containing all target sites will be considered as a sphere of radius $r_t \simeq 0.35\ \mu\mathrm{m}$ or $r_t \simeq 1.5\ \mu\mathrm{m}$ (upon transfection with LacI-VP16).
- U2OS 4A cell line: $N_i = 30$ inserts for a total of $N = 210$ target sites and a locus radius $r_t = 0.1\ \mu\mathrm{m}$.
- Effective diffusion coefficient $D^* = D_1 \cdot \tau_{3D}/(\tau_{1D} + \tau_{3D}) \simeq 6\ \mu\mathrm{m}^2 \cdot \mathrm{s}^{-1}$.

- Sliding length $l_{\mathrm{SL}} = 2\sqrt{D_{\mathrm{SL}} \cdot \tau_{\mathrm{RS}}} \simeq 250 - 750\ \mathrm{bp}$

  considering $\tau_{\mathrm{RS}} = 158\ \mathrm{ms}$ and assuming for TetR a 1D diffusion coefficient D_{SL} of the order of $10^5 - 10^6\ \mathrm{bp}^2 \cdot \mathrm{s}^{-1}$, *i.e.* similar to the values reported for several different DNA-binding proteins diffusing along naked B-DNA (49,50) and also along a chromatin lattice (51).

- Measured values of the association rate constant: $k_a = (9.2 \pm 0.2) \cdot 10^4\ \mathrm{M}^{-1} \cdot \mathrm{s}^{-1}$ for U2OS 2-6-3 cells and $k_a = (2.2 \pm 0.1) \cdot 10^4\ \mathrm{M}^{-1} \cdot \mathrm{s}^{-1}$ for U2OS 4A cells.

- Target locus occupancy at equilibrium scales linearly with N.

- Mean non-specific association time $\tau_{1D} \simeq 2\ \mathrm{s}$.

- Mean duration of a 3D excursion $\tau_{3D} \simeq 6\ \mathrm{s}$.

- DNA concentration in the nucleus $c_{\mathrm{DNA}} \simeq 10^{-2}\ \mathrm{M} \cdot \mathrm{bp}$

  considering a genome length of $3 \cdot 10^9\ \mathrm{bp}$, a nuclear volume of $500\ \mu\mathrm{m}^3$, and considering that U2OS cells are mostly triploid, we obtain:

$$
\begin{aligned}
c_{\mathrm{DNA}} &= \frac{3 \cdot 3 \cdot 10^9\ \mathrm{bp}}{500\ \mu\mathrm{m}^3 \cdot N_A} = \frac{9 \cdot 10^9\ \mathrm{bp}}{500 \cdot 10^{-15}\ \mathrm{L} \cdot 6.022 \cdot 10^{23}\ \mathrm{mol}^{-1}} = \\
&= \frac{9 \cdot 10^{24}\ \mathrm{bp} \cdot \mathrm{mol}}{500 \cdot 6.022 \cdot 10^{23}\ \mathrm{L}} = \frac{9 \cdot 10\ \mathrm{bp}}{500 \cdot 6.022}\mathrm{M} \simeq 3 \cdot 10^{-2}\ \mathrm{M} \cdot \mathrm{bp}.
\end{aligned}
$$

Computation of the association rate constant

Here below we compute the association rate constant k_a (Equation 14) considering the parameter values reported in the previous section.

$$
\begin{aligned}
k_a &\simeq \frac{l_{\mathrm{SL}}}{c_{\mathrm{DNA}}} \frac{N_i}{\tau_{1D} + \tau_{3D}} \cdot p = \\
&= \frac{300\ \mathrm{bp}}{3 \cdot 10^{-2}\ \mathrm{M} \cdot \mathrm{bp}} \frac{N_i}{2\ \mathrm{s} + 6\ \mathrm{s}} \cdot p = \\
&= 1 \cdot 10^4\ \mathrm{M}^{-1} \cdot 0.125\ \mathrm{s}^{-1} \cdot N_i \cdot p = \\
&= 2.5 \cdot 10^5\ \mathrm{M}^{-1} \cdot \mathrm{s}^{-1} \cdot p \qquad \text{for } N_i = 200 - \text{U2OS 2–6–3 cells} \\
&= 3.75 \cdot 10^4\ \mathrm{M}^{-1} \cdot \mathrm{s}^{-1} \cdot p \qquad \text{for } N_i = 30 - \text{U2OS 4A cells.}
\end{aligned}
$$

The calculated values for the association rate constant closely match the experimentally measured ones provided that $p \simeq 1$.

Supplementary Note 11

LacI behavior in mammalian cells

To test the generality of the target search mechanisms in human cells, we probed also the mobility of the LacI. In particular, we are interested in quantifying the faction of molecules undergoing to non-specific interaction *vs.* those diffusing in the solution. In fact, on one side single-molecule experiments on eukaryotic transcription factors (TFs), in eukaryotic cells, have show that the time spent in the solution (τ_{3D}) is in general longer (or of the same order) than τ_{1D}, *i.e.* the time spent while engaged in non-specific interactions (Supplementary Table 11), and this the case also for the bacterial protein TetR in mammalian cells. On the contrary, in *E. coli* cells 87% of LacI molecules are bound to non-cognate and only 13% are diffusing in the cell cytosol and the duration of the nonspecific interactions is smaller than 5 ms (52). In the purpose of investigating whether the eukaryotic nuclear environment has an influence *per se* in protein dynamics and interaction with the DNA, we studied the behavior of the LacI in U2OS cells by means of Fluorescence Recovery After Photobleaching (FRAP) and single-particle-tracking experiments (Supplementary Movie 10).

Transcription factor	Organism or cell line	τ_{1D}	τ_{3D}	Ref.
LacI	*E. coli*	< 5 ms	–	(52)
Mbp1	Yeast	0.8 s	1.1 s	(53)
p53	H1299	1.7 s	1.8 s	(54)
GR	MCF / U2OS	1.5 s	–	(55)
Sox2	Mouse ES	0.8 s	3.3 s	(56)

Supplementary Table 11 – Bound and unbound times for different transcription factors
The table lists the different TFs, the cell type/organism employed in the studies, the reported values for the time spent by TFs in the solution (τ_{3D}) or engaged in non-specific DNA-interactions (τ_{1D},), and the corresponding references.

FRAP experiments were conducted on the construct NLS-LacI-GFP in U2OS 2-6-3 cells selecting a 800 nm-radius bleaching region in the nucleoplasm, away from the target locus (Supplementary Fig. 35a).

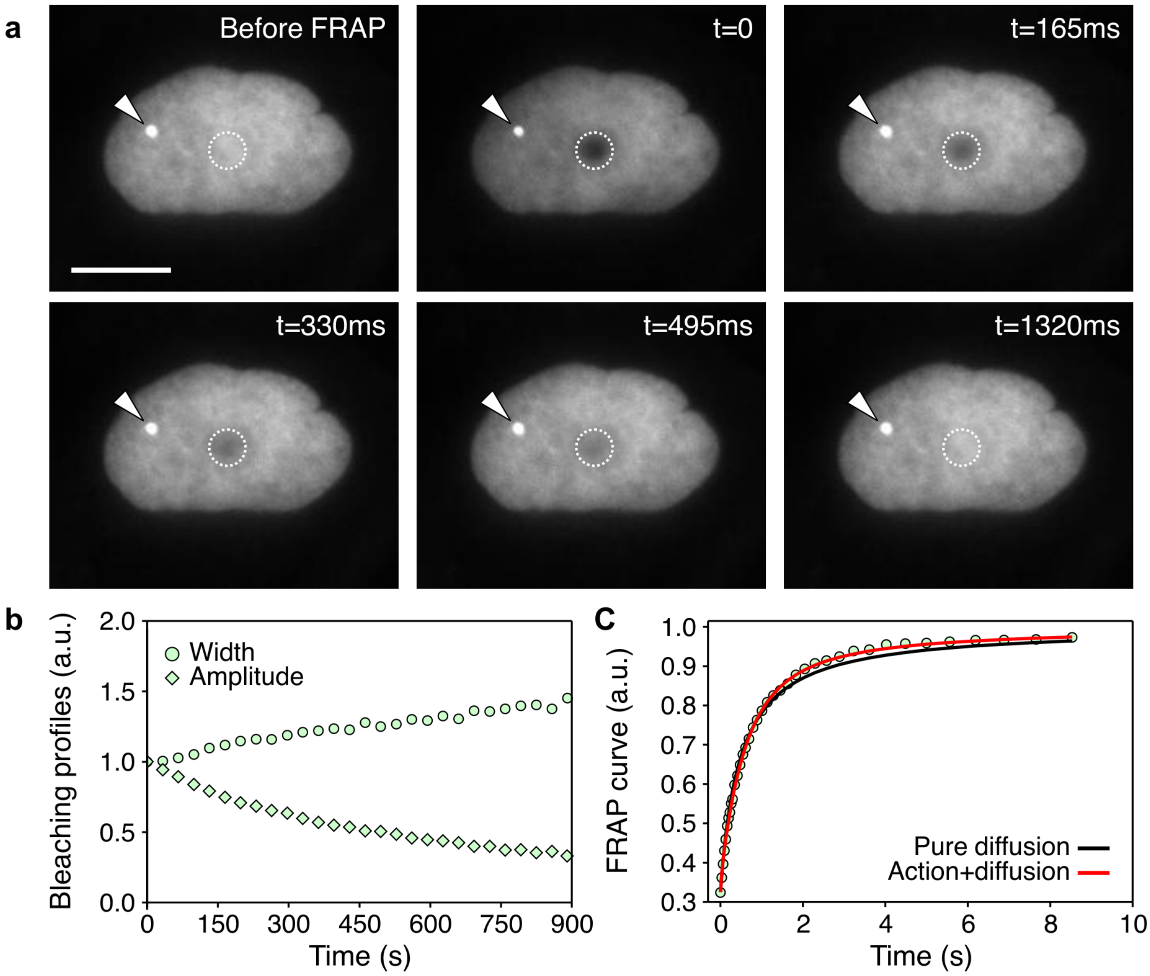

Supplementary Fig. 35 – NLS-LacI-GFP FRAP experiments

(**a**) The figure shows subsequent snap-shots of a U2OS 2-6-3 cells expressing NLS-LacI-GFP during a FRAP experiment. The white arrow points the binding site and the dashed circular line the bleaching region. Scale bar 10 μm. (**b**) The graph shows the temporal evolution of the width (circles) and of the amplitude (diamonds) of the bleached region (dashed circular line) for the cell shown in (a), as the width of the bleaching profile varies with time, diffusion plays a role at the time scale of the recovery process. (**c**) NLS-LacI-GFP FRAP curve for the cell shown in (a). Data are best described by an action-diffusion model (red curve), with mostly diffusing molecules (D = 14.6 ± 4 $\mu m^2 \cdot s^{-1}$) and about 20% of the proteins undergoing to non-specific interactions with an average binding time of 210 ± 10 ms, rather than by a purely diffusive model (black curve), which only furnishes a sort of effective diffusion coefficient (D_{eff} = 1.5 ± 0.3 $\mu m^2 \cdot s^{-1}$) slowed down cause of non-specific DNA interactions.

In particular, we used an inverted Olympus IX71 microscope, using a 100X oil immersion objective (TIRF PLANAPO 100X 1.45 NA, Olympus), a 491 laser (Cobolt Calipso 100 mW, Cobolt, Sweden) both for GFP imaging and targeted bleaching inside the cells, which was achieved with the iLAS[2]

illuminator module (Roper Scientific, Germany), and a 512 × 512 EM-CCD (EVOLVE 512, Photometrics, USA) for image acquisition. Both image acquisition and FRAP were piloted with MetaMorph (Universal Imaging, USA). Photobleaching was generated using 1,5 mW of laser power and 120 ms of total time of irradiation). The LacI mobility, *i.e.* its recovery, was monitored via time-lapse imaging with 30 ms integration time during 10 seconds after photobleaching. Data have been analyzed with the routine reported in (26) as previously described (Supplementary Note 6). Experiments have been performed at 37°C in 5% CO_2 in complete DMEM medium and cells transiently transfected overnight as described in Supplementary Note 1.

Overall, FRAP experiments showed a rapid full recovery with no stably bound molecules. As shown in Supplementary Fig. 35b, also in the case of the LacI the width of the bleaching profile broadens *vs.* time, which implies that a diffusive component is present. Thus, to quantify our FRAP data we considered both diffusion and binding. Fitting the FRAP curves to an action-diffusion model (27) yielded a diffusion coefficient D = 14.6 ± 4 $\mu m^2 \cdot s^{-1}$, a ~20% transiently interacting fraction with an average binding time of 210 ± 10 ms (Supplementary Fig. 35c). The results of the quantification of our FRAP experiments are summarized in Supplementary Table 12 and they are compatible with a scenario where most of the proteins are diffusing in solution and just a small fraction is undergoing to transient non-specific interactions with the DNA.

To directly confirm different behavior of the LacI in mammalian cells than in bacteria, we performed also single-particle-tracking experiments on the NLS-LacI-HaloTag construct.

Single-particle-tracking experiments on NLS-LacI-HaloTag proteins have been conducted on an inverted microscope (Ti Eclipse, Nikon) in wide-field configuration by focusing a 561 nm imaging laser (Cobolt Jive 200mW, Cobolt, Sweden) in the back focal plane of a 100X oil-immersion objective lens (CFI Apo TIRF 100X 1.49 NA, Nikon) and using a 512 × 512 pixels back-illuminated EM-CCD (QUANTEM, Roper Scientific, Germany) for image acquisition. Laser density of energy on the sample was on the order of 1 kW per cm^2 and laser illumination was controlled via an acousto-optic tunable filter (AOTFnC-400-650-TN, A&A Optoelectronic, France).

Experiments have been performed at 37°C in complete L15 medium and cells transiently transfected overnight as described in Supplementary Note 1. The NLS-LacI-HaloTag construct was generated subcloning the NLS-LacI-GFP plasmid. Prior to experiments cells have stained with HaloTag® TMR Ligand (G8252, Promega, USA). Specifically, cells were first washed once with 2 ml PBS, then supplemented with 2 ml of complete DMEM with a final concentration of 10nM (for bulk labeling, Supplementary Fig. 36a) and of 100 pM (for single-molecule experiments, Supplementary Fig. 36b) of HaloTag® TMR Ligand and incubated for 30 minutes at 37°C with 5% CO_2. Next, cells were rinsed twice with 2 ml of PBS, incubated with plain complete DMEM for other 30 minutes at 37°C with 5% CO_2, and finally rinsed twice with 2 ml of PBS before adding 2 ml of L15 medium (56). All reagents were used at 37°C.

	Location	Models used	D ($\mu m^2 \cdot s^{-1}$)	Bound fraction	Residence time (ms)	Number of cells
NLS-LacI-GFP	Nucleus	Diffusion+ Binding	14.6 ± 4	0.21 ± 0.1	210 ± 100	11

Supplementary Table 12 – NLS-LacI-GFP FRAP experiments fitting parameters

The table shows the values of the parameters obtained fitting the FRAP curves of 11 distinct cells with the action-diffusion model. The bound fraction has been calculated as $k_{\mathrm{off}}/(k_{\mathrm{on}} + k_{\mathrm{off}})$, and the residence time as $(1/k_{\mathrm{off}})$. Error represent s.d.

Data were acquired with 5 or 3 ms integration time under continuous illumination and analyzed as previously described (Supplementary Note 4). Single-particle tracking analysis on the NLS-LacI-HaloTag showed predominantly diffusing proteins and a small fraction (18%) of proteins transiently bound to nonspecific DNA sites (Supplementary Fig. 36b,c Supplementary Table 13). Again, non-specific interaction sites were scattered all around the nucleus (Supplementary Fig. 36b, second and forth image) and despite the limited observation time (up to few hundred of frames for the longest traces, Supplementary Fig. 36d) we could observed nonspecific binding events as long as a couple of seconds (Supplementary Fig. 36e).

Nonspecific binding times decreased with a characteristic time of about 180 ± 10 ms and again presented a long tail extending to very long residence times and deviating from a single exponential decay (Supplementary Fig. 36e inset). Interestingly, the LacI showed to some extent a higher activity than TetR at the binding site (Supplementary Fig. 36f), probably due to the higher number of LacI binding sites (51,200 *lac*O *vs.* 19,200 *tet*O sites).

The fact that also the LacI in U2OS cells behaves similarly to what reported for other DBPs (including TetR) in eukaryotic cells strengthens the view that the physical properties and the organization of the mammalian cell nuclei are very important in the nuclear biochemical reaction. Altogether, we think that our results on TetR recapitulate many common properties of the behavior of DBPs in human cells.

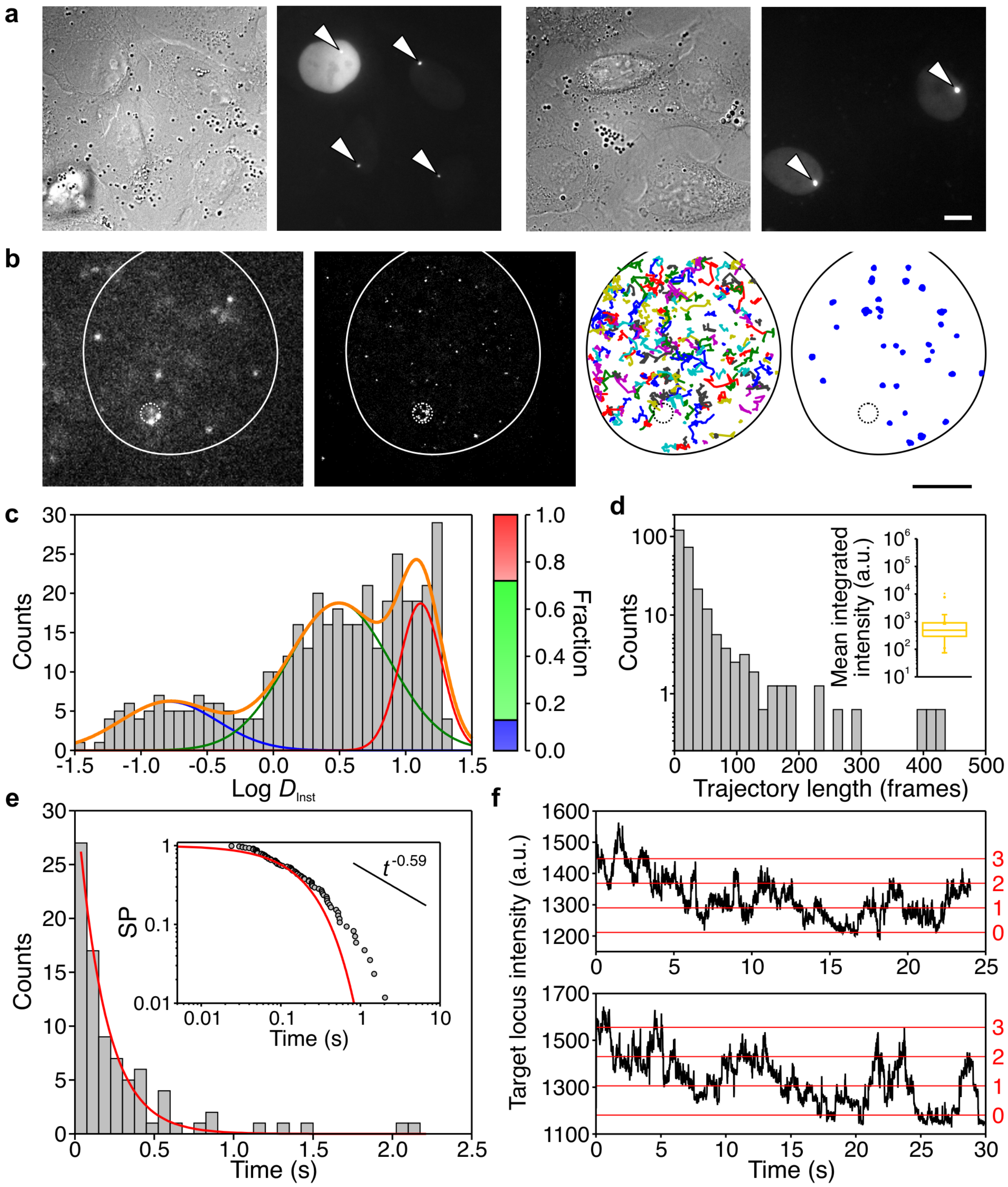

Supplementary Fig. 36 – NLS-LacI-HaloTag single-particle-tracking experiments

(**a**) Two fields of view showing each a DIC image (on the left) of U2OS 2-6-3 cells and a fluorescent image (on the right) of NLS-LacI-HaloTag protein labeled with HaloTag® TRM Ligand (10nM labeling concentration). White arrows point to the target site loci. Scale bar 10 μm. (**b**) From left to right: a 5 ms integration snapshot of the nucleus (white envelope) of a U2OS 2-6-3 cell expressing NLS-LacI-HaloTag protein and labeled with HaloTag® TRM Ligand (100 pM labeling concentration); a maximum intensity projection of all the individual detections in a 2400 frames long movie (brighter spots are the

accumulation of several distinct detections at the same location); reconstructed trajectories; and spatial distribution of nonspecifically bound proteins. The dashed white circle indicates the binding site locus. Scale bar 5 μm. (**c**) Distribution of the instantaneous diffusion coefficients D_{Inst} showing three populations with different mobility; central values of the instantaneous diffusion coefficients and population abundances are summarized in Supplementary Table 13. (**d**) The histogram report the distribution of the trajectory length (expressed in number of frames) obtained for the NLS-LacI-HaloTag protein labeled with HaloTag® TMR Ligand, the inset shows the distribution of the mean integrated intensity of the spot localizations along an individual trajectory. (**e**) Distribution of the duration of the non-specific binding events, the red line corresponds to a mono-exponential fit with a characterizing decay time of 180 ± 10 ms. The inset shows the SP of the nonspecific binding events, the red line represents the fitted exponential decay, and the black line is a guide-to-eye corresponding to a power-law t^{γ} with γ = -0.59 ± 0.01. (**f**) The two traces report the binding site locus intensity as a function of the time for two distinct cells. The red scale on the left is an indication of the number of proteins bound at the target site.

**NLS-LacI-HaloTag**	
D_1 ($\mu m^2 \cdot s^{-1}$)	12.9
f_1	0.24
D_2 ($\mu m^2 \cdot s^{-1}$)	3.1
f_2	0.58
D_3 ($\mu m^2 \cdot s^{-1}$)	0.15
f_3	0.18
Number of trajectories	401
Mean trajectory length (frames)	30
Number of cells	3

Supplementary Table 13 – NLS-LacI-HaloTag single-particle-tracking experiments

The table shows the central values of the instantaneous diffusion coefficients (D_{Inst}) and the fraction *f* of the different populations (1: fast freely diffusing proteins; 2: intermediate population; 3: quasi-immobile proteins) observed in our SPT experiments on NLS-LacI-HaloTag. The table reports also the statistics regarding the number of cells observed and the number of trajectories used in the analysis, as well as the mean trajectory length.

Supplementary References

1. Janicki, S. M. *et al.* From silencing to gene expression: Real-time analysis in single cells. *Cell* **116,** 683–698 (2004).
2. Causse, S. Study of the activation dynamics of gene transcrption by RNA Polymerase 2. *Ph.D. Thesis* - Université Pierre et Marie Curie, Paris (2011).
3. Cisse, I. I. *et al.* Real-time dynamics of RNA polymerase II clustering in live human cells. *Science* **341,** 664–667 (2013).
4. Tumbar, T., Sudlow, G. & Belmont, A. S. Large-scale chromatin unfolding and remodeling induced by VP16 acidic activation domain. *J. Cell Biol.* **145,**1341–1354 (1999).
5. Mendez, B., Tachibana, C. & Levy, S. B. Heterogeneity of tetracycline resistance determinants. *Plasmid* **3,** 99–108 (1980).
6. Saenger, W., Orth, P., Kisker, C., Hillen, W. & Hinrichs, W. The Tetracycline repressor: A paradigm for a biological switch. *Angew. Chem. Int. Ed.* **39,** 2042–2052 (2000).
7. Krueger, C., Pfleiderer, K., Hillen, W. & Berens, C. Tetracycline derivatives: alternative effectors for Tet transregulators. *BioTechniques* **37,** 546–550 (2004).
8. Kleinschmidt, C., Tovar, K., Hillen, W. & Porschke, D. Dynamics of repressor-operator recognition: Tn10-encoded tetracycline resistance control. *Biochemistry* **27,** 1094–1104 (1988).
9. Hinrichs, W. *et al.* Structure of the Tet repressor-tetracycline complex and regulation of antibiotic resistance. *Science* **264,** 418–420 (1994).
10. Gossen, M. *et al.* Transcriptional activation by tetracyclines in mammalian cells. *Science* **268,** 1766–1769 (1995).
11. Urlinger, S. *et al.* Exploring the sequence space for tetracycline-dependent transcriptional activators: Novel mutations yield expanded range and sensitivity. *Proc. Natl Acad. Sci. USA* **97,** 7963–7968 (2000).
12. Gurskaya, N. G. *et al.* Engineering of a monomeric green-to-red photoactivatable fluorescent protein induced by blue light. *Nat. Biotech.* **24,** 461–465 (2006).
13. Darzacq, X. *et al.* In vivo dynamics of RNA polymerase II transcription. *Nat. Struct. Mol. Biol.* **14,** 796–806 (2007).
14. HT1080 Cell Line and pTRE2 Vector. Clontechniques **XIV,** 23 (1999).
15. Tsukamoto, T. *et al.* Visualization of gene activity in living cells. *Nat. Cell Biol.* **2,** 871–878 (2000).
16. Serge, A., Bertaux, N., Rigneault, H. & Marguet, D. Dynamic multiple-target tracing to probe spatiotemporal cartography of cell membranes. *Nat. Methods* **5,** 687–694 (2008).
17. Qian, H., Sheetz, M. P. & Elson, E. L. Single particle tracking. Analysis of diffusion and flow in two-dimensional systems. *Biophys. J.* **60,** 910–921 (1991).
18. Diez-Ahedo, R. *et al.* Dynamic re-organization of individual adhesion nanoclusters in living cells by ligand-patterned surfaces. *Small* **5,** 1258–1263 (2009).

19. Pinaud, F. *et al.* Dynamic partitioning of a glycosyl-phosphatidylinositol-anchored protein in glycosphingolipid-rich microdomains Imaged by single-quantum dot tracking. *Traffic* **10,** 691–712 (2009).
20. Saxton, M. J. Lateral diffusion in an archipelago. Single-particle diffusion. *Biophys. J.* **64,** 1766–1780 (1993).
21. Saxton, M. J. & Jacobson, K. Single-Particle Tracking: Applications to membrane dynamics. *Annu. Rev. Biophys. Biomol. Struct.* **26,** 373–399 (1997).
22. Manley, S. *et al.* High-density mapping of single-molecule trajectories with photoactivated localization microscopy. *Nat. Methods* **5,** 155–157 (2008).
23. Izeddin, I. *et al.* Single-molecule tracking in live cells reveals distinct target-search strategies of transcription factors in the nucleus. *eLife* **3,** e02230(27) (2014) – doi: 10.7554/eLife.02230.
24. Tokunaga, M., Imamoto, N. & Sakata-Sogawa, K. Highly inclined thin illumination enables clear single-molecule imaging in cells. *Nat. Methods* **5,** 159–161 (2008).
25. Schütz, G. J., Schindler, H. & Schmidt, T. Single-molecule microscopy on model membranes reveals anomalous diffusion. *Biophys. J.* **73,** 1073–1080 (1997).
26. Mueller, F., Wach, P. & McNally, J. G. Evidence for a common mode of transcription factor interaction with chromatin as revealed by improved quantitative Fluorescence Recovery After Photobleaching. *Biophys. J.* **94,** 3323–3339 (2008).
27. Mueller, F., Mazza, D., Stasevich, T. J. & McNally, J. G. FRAP and kinetic modeling in the analysis of nuclear protein dynamics: what do we really know? *Curr. Opin. Cell Biol.* **22,** 403–411 (2011).
28. Beaudouin, J., Mora-Bermúdez, F., Klee, T., Daigle, N. & Ellenberg, J. Dissecting the contribution of diffusion and interactions to the mobility of nuclear proteins. *Biophys. J.* **90,** 1878–1894 (2006).
29. Phair, R. D., Gorski, S. A. & Misteli, T. Measurement of dynamic protein binding to chromatin in vivo, using photobleaching microscopy. In *Methods in Enzymology*. Allis, C. D. & Carl, W. Editors. Academic Press, New York. pp. 393–414 (2004).
30. Sprague, B. L., Pego, R. L., Stavreva, D. A. & McNally, J. G. Analysis of binding reactions by Fluorescence Recovery After Photobleaching. *Biophys. J.* **86,** 3473–3495 (2004).
31. Mueller, F., Karpova, T. S., Mazza, D. & McNally J. G. Monitoring dynamic binding of chromatin proteins in vivo by Fluorescence Recovery After Photobleaching. In *Chromatin Remodeling. Methods and Protocols.* Morse, R. H. Editor. Humana Press. *Methods in Molecular Biology* **833,** 153–176 (2012).
32. Colquhoun, D. & Sigworth, F. J. Fitting and statistical analysis of single-channel records. In *Single-Channel Recording*. Sakmann, B. & Neher, E. Editors. Plenum Press, New York. pp. 191–263 (1983).
33. Vanzi, F., Sacconi, L. & Pavone, F. S. Analysis of kinetics in noisy systems: Application to single molecule tethered particle motion. *Biophys. J.* **93,** 21–36 (2007).

34. Loverdo, C. *et al.* Quantifying Hopping and Jumping in Facilitated Diffusion of DNA-Binding Proteins. *Phys. Rev. Lett.* **102,**188101 (2009).
35. Redner, A Guide to First-Passage Processes, *Cambridge University Press*, Cambridge - England, (2001).
36. Mütze, J. *et al.* Excitation spectra and brightness optimization of two-photon excited probes. *Biophys. J.* **102,** 934–944 (2012).
37. Akerboom, J. *et al.* Optimization of a GCaMP calcium indicator for neural activity imaging. *J. Neurosci.* **32,** 13819–13840 (2012).
38. Schwille, P., Haupts, U., Maiti, S. & Webb, W.W. Molecular dynamics in living cells observed by Fluorescence Correlation Spectroscopy with one- and two-photon excitation. *Biophys. J.* **77,** 2251–2265 (1999).
39. Kamionka, A., Bogdanska-Urbaniak, J., Scholz, O. & Hillen, W. Two mutations in the tetracycline repressor change the inducer anhydrotetracycline to a corepressor. *Nucleic Acids Res.* **32,** 842–847 (2004).
40. Mueller, F. *et al.* FISH-quant: automatic counting of transcripts in 3D FISH images. *Nat. Methods* **10,** 277–278 (2013).
41. Izeddin, I. *et al.* PSF shaping using adaptive optics for three-dimensional single-molecule super-resolution imaging and tracking. *Opt. Express* **20,** 4957–4967 (2012), http://dx.doi.org/10.1364/OE.20.004957.
42. Azucena, O. *et al.* Adaptive optics wide-field microscopy using direct wavefront sensing. *Opt. Lett.* **36,** 825–827 (2010).
43. Kner, P., Sedat, J. W., Agard, D. A. & Kam, Z. High-resolution wide-field microscopy with adaptive optics for spherical aberration correction and motionless focusing. *J. Microsc.* **237,** 136–147 (2010).
44. Clouvel, G. *et al.* Dual-color 3D PALM/dSTORM imaging of centrosomal proteins using MicAO 3DSR. *Proc. SPIE* **8590**, Single Molecule Spectroscopy and Superresolution Imaging VI, 85900Z (2013) – doi: 10-1117/12.2001986.
45. Kao, H. P. & Verkman, A. S. Tracking of single fluorescent particles in three dimensions: use of cylindrical optics to encode particle position. *Biophys. J.* **67,** 1291–1300 (1994).
46. Huang, B., Wang, W., Bates, M. & Zhuang, X. Three-dimensional super-resolution imaging by stochastic optical reconstruction microscopy. *Science* **319,** 810–813 (2008).
47. El Beheiry, M. & Dahan, M. ViSP: representing single-particle localizations in three dimensions. *Nat. Methods* **10,** 689–690 (2013).
48. Sheinman, M., Bénichou, O., Kafri, Y. & Voituriez, R. Classes of fast and specific search mechanisms for proteins on DNA. *Rep. Prog. Phys.* **75,** 026601 (2012).
49. Bonnet, I. *et al.* Sliding and jumping of single EcoRV restriction enzymes on non-cognate DNA. *Nucleic Acids Res.* **36,** 4118–4127 (2008).

50. Blainey, P. C. *et al.* Nonspecifically bound proteins spin while diffusing along DNA. *Nat. Struct. Mol. Biol.* **16,** 1224–1229 (2009).
51. Gorman, J., Plys, A. J., Visnapuu, M.-L., Alani, E. & Greene, E. C. Visualizing one dimensional diffusion of eukaryotic DNA repair factors along a chromatin lattice. *Nat. Struct. Mol. Biol.* **17,** 932–938 (2010).
52. Elf, J., Li, G.-W. & Xie, X. S. Probing transcription factor dynamics at the single-molecule level in a living cell. *Science* **316,** 1191–1194 (2007).
53. Larson, D. R., Zenklusen, D., Wu, B., Chao, J. A. & Singer R. H. Real-time observation of transcription initiation and elongation on an endogenous yeast gene. *Science* **332,** 475-478 (2011).
54. Mazza, D., Abernathy, A., Golob, N., Morisaki, T. & McNally, J. G. A benchmark for chromatin binding measurements in live cells. *Nucleic Acids Res.* **40,** *e119* (2012).
55. Gebhardt, J. C. *et al.* Single-molecule imaging of transcription factor binding to DNA in live mammalian cells. *Nat. Methods* **10,** 421–426 (2013).
56. Chen, J. *et al.* Single-molecule dynamics of enhanceosome assembly in embryonic stem cells. *Cell* **156,** 1274–1285 (2014).
